# The effectiveness and safety of physical activity and exercise on women with endometriosis: A systematic review and meta-analysis

**DOI:** 10.1371/journal.pone.0317820

**Published:** 2025-02-13

**Authors:** Min Xie, Xuemei Qing, Hailong Huang, Linyun Zhang, Qin Tu, Hongying Guo, Jing Zhang

**Affiliations:** 1 Department of Obstetrics and Gynecology, Chengdu Qingbaijiang District People’s Hospital, Chengdu, China; 2 Department of Obstetrics and Gynecology, West China Second University Hospital, Sichuan University, Chengdu, China; 3 Key Laboratory of Birth Defects and Related Diseases of Women and Children (Sichuan University), Ministry of Education, Chengdu, China; 4 Reproductive Endocrinology and Regulation Laboratory, West China Second University Hospital, Sichuan University, Chengdu, China; 5 The Joint Laboratory for Reproductive Medicine of Sichuan University, The Chinese University of Hong Kong, Chengdu, China; Dipartimento di Scienze Mediche e Chirugiche (DIMEC), Orsola Hospital, ITALY

## Abstract

**Background:**

Endometriosis is a debilitating, chronic disease that affects approximately 10% of women of reproductive age worldwide. The most common symptom is chronic pelvic pain, which leads to a reduced quality of life and requires lifelong treatment. The current standard of care for endometriosis is pain management, which consists mainly of medical and surgical treatment. Appropriate physical activity (PA) and exercise can help manage both physical and psychological symptoms of chronic conditions. Consequently, this systematic review and meta-analysis was designed to assess the effectiveness and safety of PA and exercise in women with endometriosis.

**Methods:**

We searched the published literature in Pubmed, Medline, Embase, The Cochrane Library, and Web of Science. Randomized controlled trials (RCTs) were obtained to assess the effects of physical activity and exercise on women with endometriosis. The random or fixed effects model was used to analyze the data in meta-analysis. The results were expressed as weighted mean differences (WMD) and their corresponding 95% confidence intervals (CIs).

**Results:**

Six RCTs were identified in our systematic review, involving 251 patients. The results indicated that physical activity and exercise have a beneficial impact on quality of life, pain intensity, mental health, pelvic floor dysfunction, and bone density. However, due to the heterogeneity of the outcome measures and the incomplete reporting of the results in the studies included in this review, only a simple meta-analysis of two studies could be performed. The meta-analysis demonstrated that physical activity and exercise have a significant impact on the improvement of quality of life, particularly in the context of pain (P <0.0001), control and powerlessness (P <0.00001), and emotional well-being (P = 0.006).

**Conclusion:**

The present review indicates that physical activity and exercise have beneficial effects on the treatment of symptoms associated with endometriosis, particularly in terms of improving quality of life and providing pain relief. Due to the limitation in the quality of involved studies and the short duration of treatment, more RCTs with high-quality, long-term duration are needed for further validation.

**Trial registration:**

**Systematic review registration:** Registration number: CRD 42024547551.

## Introduction

Endometriosis is a benign, chronic, inflammatory, and estrogen-dependent disease affecting approximately 10% of reproductive-age women globally [1]. The disease is defined by the presence of endometrial-like tissue in locations external to the uterus, with the potential for involvement of the gastrointestinal, urinary, and musculoskeletal systems [2]. Endometriosis is currently regarded as a systemic disease, rather than a condition that is primarily confined to the pelvis [3]. The clinical presentation is diverse, and in addition to the symptoms of endometriosis itself, there are organ-specific symptoms associated with the endometriotic lesions [4]. The most prevalent clinical manifestations are infertility and pain symptoms such as dysmenorrhea, dyspareunia, and chronic pelvic pain [5]. Endometriosis significantly affects patients’ quality of life, affecting numerous aspects of their daily lives, including activities of daily living, sexual function, and personal relationships. Furthermore, the disease is associated with an increased risk of depression, fatigue, and reduced work productivity [6]. Endometriosis has a significant impact on public health, resulting in considerable healthcare expenditures [7]. The economic burden exceeds US$22 billion in the USA alone and £12.5bn in the UK [8].

The etiology of endometriosis remains unclear. A number of pathogenetic mechanisms have been proposed to explain the etiology of endometriosis, including the stem cell theory, the coelomic metaplastic theory, the Sampson theory, the Müllerian remnant theory, and the vascular and lymphatic metastasis theory [9]. The retrograde menstruation theory proposed by Sampson is widely accepted. Notably, endometriosis is primarily associated with chronic pelvic pain that results from the activation of macrophages and mast cells, thereby contributing to a persistent cycle of inflammation, oxidative stress, and pain [10]. Nevertheless, the etiology of endometriosis remains poorly understood. The diagnosis is frequently delayed due to inadequate knowledge of the disease and the absence of reliable non-invasive biomarkers, with the interval between initial symptoms and diagnosis ranging from four to 11 years. It has been documented that 65% of women are initially misdiagnosed [11].

Endometriosis is a chronic disease with no known cure and requires lifelong management. The current standard of care for endometriosis is pain management, which may include medication, surgery, or a combination of both [12]. Nevertheless, studies have demonstrated that these treatments are relatively efficacious in reducing pain and that the disease and its symptoms have a high rate of recurrence [13]. The importance of complementary therapies is increasingly being emphasized. The main types of complementary therapies are acupuncture, physical activity, exercise, and dietary interventions [14–16]. The international clinical guidelines recommend that the role of physical activity (PA) and exercise be given particular attention in the management of endometriosis-related symptoms [17].

The efficacy of physical activity and exercise as an aid to pain management in endometriosis has been the subject of recent research. A systematic review suggests that exercise has a beneficial effect on pain in women with endometriosis. However, it was not possible to determine the effectiveness of PA and exercise in treating endometriosis-related symptoms [18]. Another two systematic reviews could not determine the effect of PA and exercise on endometriosis-associated symptoms[19, 20]. However, a narrative review found that body awareness exercises such as Hatha yoga, progressive muscle relaxation, and the Jacobson method reduced pain and stress and improved the quality of life associated with endometriosis [21]. The studies included in these reviews employ a variety of research methods and designs, including cross-sectional surveys, cohort studies, observational studies, and randomized controlled trials. Moreover, randomized controlled trials are currently scarce. Furthermore, the quality of evidence derived from these studies is relatively low. The aim of this systematic review and meta-analysis is to systematically review the available randomized controlled trials to assess the effectiveness and safety of physical activity and exercise on endometriosis.

## Methods

This systematic review and meta-analysis was designed and conducted following a predetermined protocol according to the Cochrane Handbook’s recommendations [22]. We reported the results using the Preferred Reporting Items for Systematic Reviews and Meta-Analyses (PRISMA), a systematic review guideline [23]. Before data extraction, the systematic review was registered in the International Prospective Register of Systematic Reviews (PROSPERO) database (CRD 42024547551).

### Search strategies

The literature search was performed in Pubmed, Embase, The Cochrane Library, Medline, and Web of Science from inception until May 2024. No limits were applied for language and publication date. Additionally, the reference lists of included articles, relevant reviews, and grey literature on the topic were manually searched for additional studies. Two review authors assessed the eligibility of documents. Any disagreements were resolved by discussion with the corresponding author. The electronic search strategy for this systematic review and meta-analysis is presented in Table S1 in **S1 File**.

### Eligibility criteria

Studies were included in the systematic review if they met the following criteria: the participants had to be women who had been diagnosed with any degree of endometriosis; the intervention of studies involving any type of physical activity and exercise; the control group engaged in activities of daily living and/or conventional treatment; and only randomized controlled trials (RCTs) were included. The primary outcome indicator was quality of life, but all outcomes were accepted for consideration.

The exclusion criteria were as follows: Conference abstracts, animal studies, cohort studies, retrospective studies, non-randomized controlled trials, and non-English literature. No attempt was made to identify unpublished studies.

### Study selection criteria

We conducted the initial search, removed duplicate records, and screened titles and abstracts for relevance. Records were then identified as included, excluded, or uncertain. In cases of uncertainty, the full text was obtained to determine eligibility. Identified documents were independently assessed by two authors. In case of disagreement, a third author was consulted to make a final judgment.

### Data extraction

Data extraction was performed independently by two authors, who thoroughly reviewed each included document. Data were cross-checked to minimize potential errors, and disagreements were resolved by discussion with the corresponding author. Cochrane guidelines were used to extract the characteristics of the included studies. The following information was extracted from the included studies: author name, country of origin, year of publication, study period, sample size, description of intervention, description of control, duration, primary and secondary outcomes, and dropout rate. For continuous variables, the data were extracted as the mean and standard deviation (SD) for the post-intervention values. In the absence of a specified mean difference (MD), it was derived from either the standard error, interquartile range, or the 95% confidence interval. We strove to maintain the integrity of the data, and any missing data were obtained through email or telephone communication with the authors. If no response is received, the study will be excluded.

### Data synthesis and analysis

All related statistical analysis was conducted by using the software Review Manager 5.4. The pooled effect sizes were considered as weighted mean differences (WMDs) with 95% confidence intervals (95% CI). If multiple time points were reported for outcomes, the data from the final time point was used for analysis. Due to the lack of data, the minimum number of studies for the meta-analysis was decreased to two. Heterogeneity between studies was estimated using the Cochrane Q static with its P value and the I-squared (I^2^) statistic (degree of heterogeneity). In each analysis, heterogeneity was presented as low (I^2^< 40%), moderate (40% < I^2^ ≤70%), or high (I^2^ > 70%). The fixed-effects model was applied when I^2^ <50%. Otherwise, the random-effects model was conducted. p < 0.05 represented statistical significance.

### Assessment of risk of bias

Two independent researchers conducted the quality assessment by using the Cochrane Collaboration’s risk of bias tool for RCTs. The analysis was carefully reviewed, and any discrepancies were resolved through consultation with a third researcher. The Cochrane Risk Assessment Tool has been used to assess quality at the study level. Factors related to the tool included random sequence generation, allocation concealment, application of blinding method, incomplete outcome data, selective outcome reporting, and other sources of bias. Every domain was assessed and given a verdict of "yes," "no," or "unclear" based on The Cochrane Collaboration’s standards. We assigned three categories—unclear, low, and high risk of bias—for our risk-of-bias judgments.

## Results

### Studies selection and the flow chart

**Fig 1:**
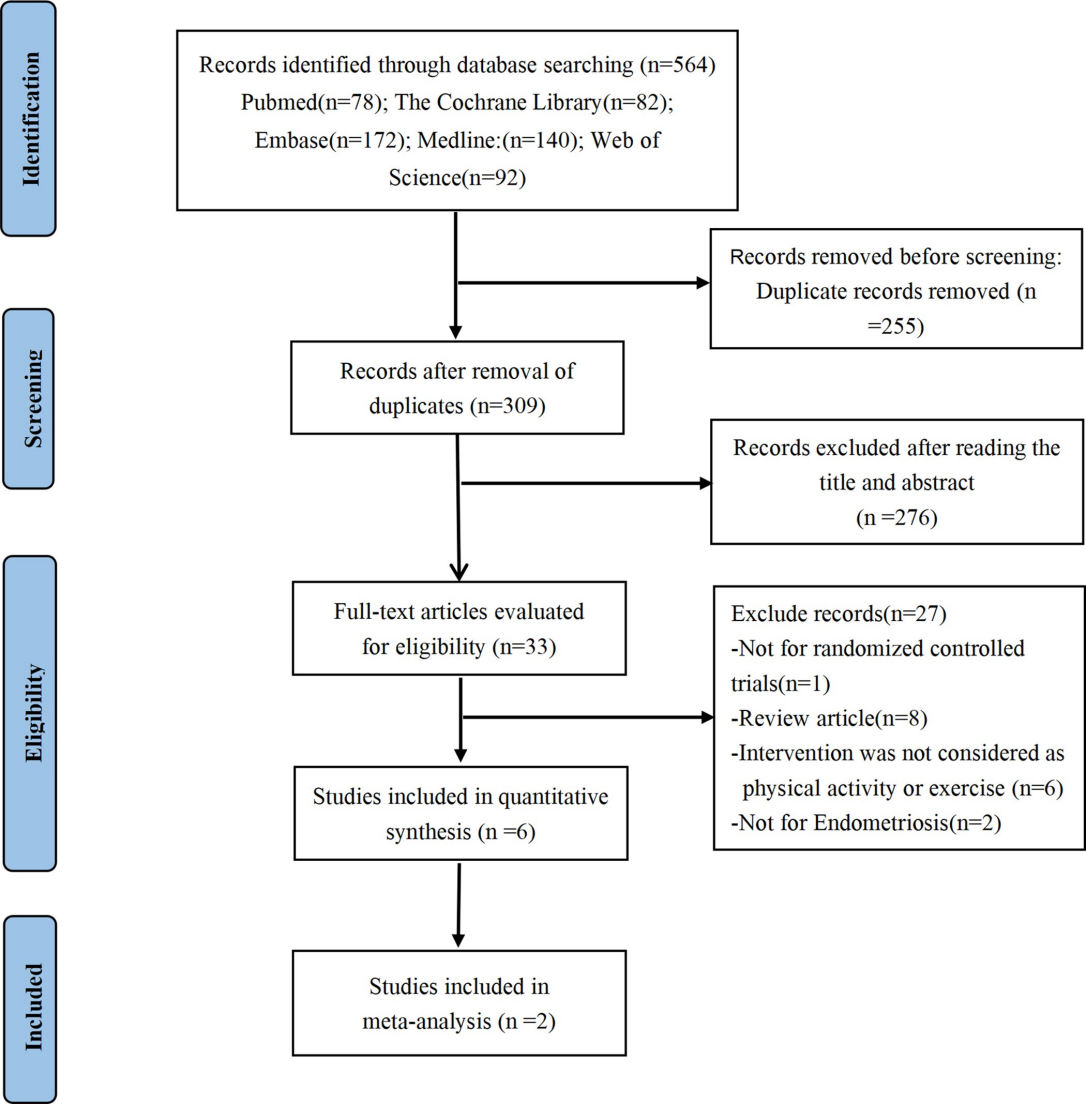
PRISMA flow diagram of the study process. PRISMA, Preferred Reporting Items for Systematic Review and Meta-analysis.

The preliminary search identified 564 documents, including 78 from PubMed, 172 from Embase, 82 from Cochrane Library, 140 from Medline, and 92 from Web of Science. After screening by Endnote, removing 255 duplicate articles, 309 documents were assessed by screening titles and abstracts. Among them, 276 were excluded due to apparent ineligibility. 33 documents were selected for full-text evaluation, and 27 of these were excluded for the following reasons: non-randomized controlled trials (n = 1); review article(n = 8); intervention was not considered as physical activity or exercise (n = 6); not for endometriosis(n = 2); no available data (n = 4); uncompleted studies(n = 6). Finally, this systematic review fully synthesized six RCTs (251 participants) [24–29]. Further, due to homogeneity, two RCTs [25, 29] were included for meta-analysis. Details of the selection process have been shown in the PRISMA flow diagram (**Fig 1**).

### Characteristics of included studies

The summarized characteristics of the six RCTs included in the review are shown in **Table 1**. One study was conducted in Spain [24], one in Brazil [25], one in Australia [29], one in Sweden [27], one in China [26], and one in the United States of America [28]. All of the included studies were randomized controlled trials conducted between 1995 and 2023. The total study sample consisted of 251 women with endometriosis. The study women were aged between 16 and 51 years, two of the studies did not report participant ages, only the mean value [24, 28]. Three studies included women with surgically confirmed endometriosis [26–28], while three studies did not specify the diagnostic criteria used for endometriosis [24, 25, 29]. The specific stages of endometriosis were not delineated in any of the studies. All women in one study also exhibited chronic pelvic pain [25]. All women included in the two studies did not engage in any form of exercise or yoga in the past [25, 28]. All women in one study demonstrated endometriosis-related discomfort (i.e., painful intercourse, dysmenorrhea, or chronic pelvic pain) [26].

**Table 1:** Characteristics of the included studies.

Study	Country	Publish year	Recruitment	Number	Study population	Intervention description	Control group	Duration	primary outcomes	Secondary outcome	Dropouts
**Artacho-Cordón. et al. [24]**	**Spain**	**2023**	**2020–2021**	**31 (16 interventions vs 15 controls)**	**endometriosis** **unresponsive to conventional therapy**	**1-week lumbopelvic stabilization learning phase 8-week phase of** **stretching, aerobic, and resistance exercises focused on the lumbopelvic sequentially instructed and supervised by a trained physiotherapist**	**usual treatments**	**9 weeks and 1-year follow-up**	**QOL(EHP-30)**	**PPTs, pain intensity, and pain-related catastrophizing thoughts, isometric resistance of abdominal and lumbar muscles, lumbopelvic stability, and muscle architecture of the abdominal wall and lumbar multifidus**	**4(12.9%),3 in the intervention group, 1 in the control group**
**Gonçalves. et al. [25]**	**Brazil**	**2017**	**2013.08–2014.12**	**40 (28 interventions vs 12** **controls)**	**Endometriosis** **and CPP, prior** **hormonal and surgical therapy, age34.88 ± 6.70 years, no regular exercise**	**Supervised;120 min of Hatha yoga, posture** **(60 min) + conversation** **(30 min) + relaxation, breathing exercises, meditation (30 min) Medical therapy was continued**	**Continuing medical** **therapy or** **physiotherapy** **once per week**	**Twice weekly** **for 8 weeks**	**QOL (EHP-30)**	**Pain (VAS,0–10), menstrual pattern measured daily (amount of bleeding scored from 0 to 5)**	**12 (30%), only** **in intervention** **group**
**Lutfi et al. [29]**	**Australia**	**2023**	**2021.01–2022.09**	**22 (8 telehealth-delivered exercises; 8 VR-delivered exercises; 6 controls.)**	**Endometriosis patients without contraindications to exercise and visual impairment, age 18-45years**	**attend one supervised exercise or telehealth-delivered exercise training session**	**activities of daily living**	**48 hours**	**Pain (VAS,0–10)**	**NR**	**3(13.6%),1 in the telehealth-delivered exercise group, 2 in the control group**
**Carpenter et al. [28]**	**USA**	**1995**	**NR**	**39 (18 interventions vs 18** **controls)**	**Endometriosis** **with no other hormonal treatment** **during previous** **12 months, no** **regular exercise**	**Unsupervised; 40 min of individualized cardio fitness at 50–70% of max heart rate + flexibility exercises + danazol**	**Danazol treatment only**	**Four times** **weekly for** **24 weeks**	**Number of** **side effects** **of danazol** **(direct inquiry)**	**Fitness (VO2max),** **general muscle strength** **(KINCOM), sex hormone** **levels, pelvic symptoms**	**3 (7.69%)**
**Bergström et al. [27]**	**Sweden**	**2005**	**NR**	**19(8 interventions vs 11** **controls)**	**endometriosis,** **no GnRH analogs, no other diseases or medications that** **could interfere with the BMD. age 23–38 years**	**three 30-minute fast walks and two 1-h aerobic training sessions a week + GnRH treatment**	**activities of daily living + GnRH treatment**	**Every six months for 12 months**	**BMD**	**NR**	**3(15.7%),2 in the intervention group, 1 in the control group**
**Zhao et al. [26]**	**China**	**2012**	**2010.01–2010.11**	**100(50 interventions vs 50** **Controls)**	**Chinese endometriosis, age 18–48 years**	**twenty-four 40-min group PMR practice sessions over 12 weeks, twice per week. +depot leuprolide**	**activities of daily living +depot leuprolide**	**12weeks**	**QOL(SF-36)**	**anxiety, depression**	**13(13%), 8 in the intervention group, 5 in the control group**

**QOL** quality of life, **PPTs** pressure pain thresholds, **CPP** chronic pelvic pain, **VAS** visual analog scale, **KINCOM** Kinetic Communicator Exercise System, **BMD** bone mineral density, **SF-36** 36-item Short-Form Health Survey, **EHP-30** Endometriosis Health Profile-30, **PMR** progressive muscular relaxation, **GnRH** gonadotrophin-releasing hormone, **VR** virtual reality, **NR** not reported.

The performed interventions are listed in **Table 1**, which included stretching, aerobic, and resistance exercises that were focused on the lumbar spine; hatha yoga; virtual reality-delivered exercises; telehealth-delivered exercises; 40 minutes of personalized aerobic fitness at 50–70% of maximal heart rate + flexibility exercises; fast walks and aerobic training; and progressive muscle relaxation exercises. All studies included pre- and post-treatment examinations. Three studies were evaluated during treatment [25, 27, 28], and two studies had long-term follow-up [24, 28].

**Table 1** presents a summary of the primary and secondary outcomes for all studies included in the systematic review. The primary outcomes of the studies included quality of life, pain, reduction of adverse effects as described in the package insert of the drug, and bone mineral density. However, the outcome reports were incomplete for all studies. The primary indicator value for quality of life was assessed in three studies [24, 25]. Two outcomes were evaluated using the Endometriosis Health Profile-30(EHP-30), while one study employed the 36-item Short-Form Health Survey (SF-36). The assessment of pelvic pain was conducted using the visual analog scale (VAS) in two studies.

### Risk bias in included studies

**Fig 2:**
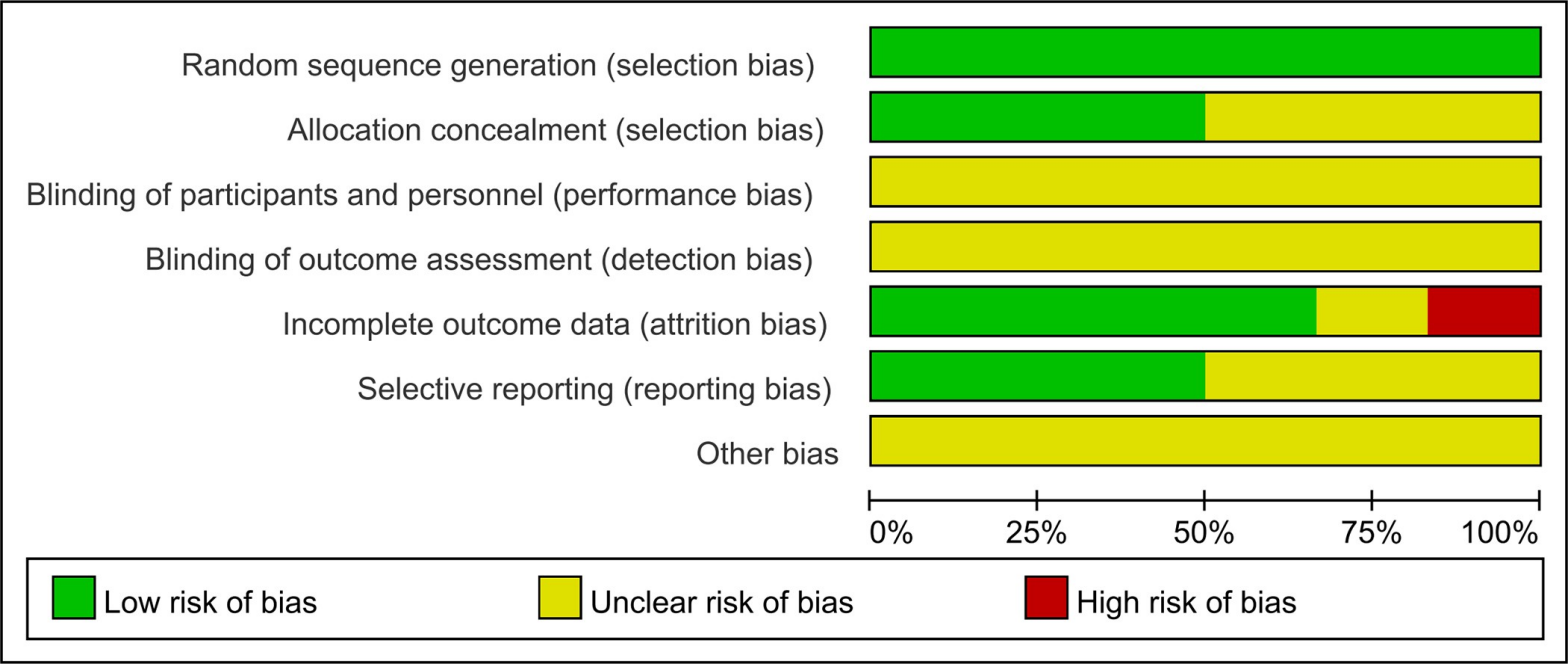
Overall risk of bias assessment.

**Fig 3:**
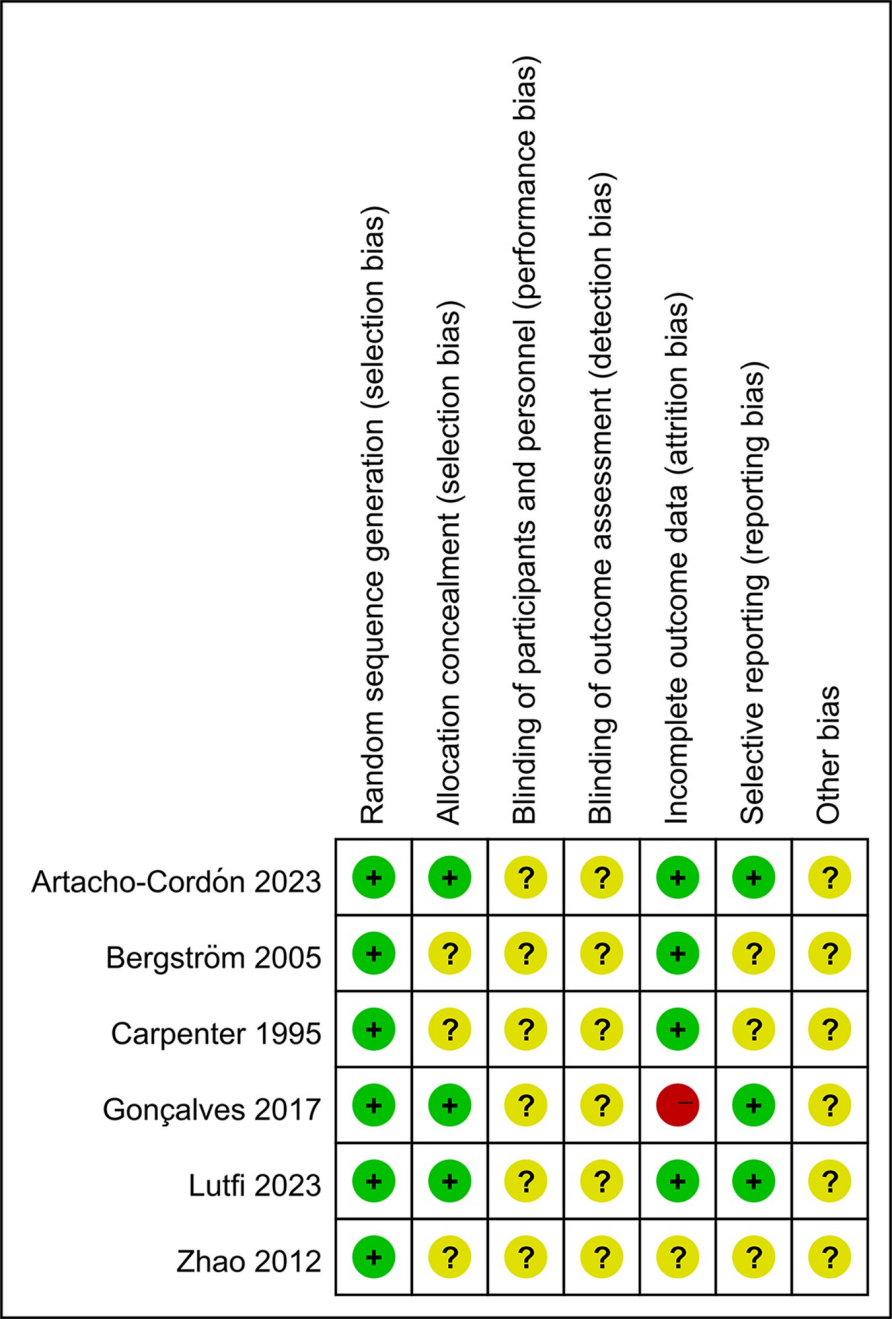
Risk of bias summary for randomized controlled trials.

All studies were assessed for potential sources of bias using the Cochrane tool. The results were summarized following the risk categories proposed by the tool, as illustrated in **Figs 2 and 3**. All studies were found to have adequately generated random sequences, as described in the respective articles, and were therefore determined to be at low risk of bias. Concerning the allocation process, only three of the six studies that employed random sequence generation described how the allocation was conducted [24, 25, 29]. Consequently, these studies were classified as being at low risk of bias. With regard to the masking of participants and outcomes, all studies that did not attempt to mask either participants or personnel were classified as having an uncertain risk of bias. Regarding incomplete outcome data, one study did not provide information regarding the proportion of missing visits, thus placing it at a high risk of bias [25]. Another study did not adequately describe data loss, resulting in an uncertain risk of bias [26]. However, four other studies did provide explanations for any data loss, thus placing them at low risk of bias [24, 27–29]. Regarding selective reporting, only three studies provided comprehensive descriptions of both primary and secondary outcomes [24, 25]. The remaining three studies did not provide sufficient detail on this aspect and were therefore classified as having an uncertain risk of bias [26–29]. A comprehensive assessment of all studies revealed that none provided sufficient information to enable an evaluation of the risk of bias.

### Study losses

All six studies incorporated intention-to-treat analyses and reported on study losses. However, the study by Zhao et al. [26]did not provide an explanation for the reasons why women were excluded or lost to follow-up. The study by Gonçalves et al. [25] revealed that key data were measured for less than 85% of the population due to loss of follow-up. Specifically, 40 women were enrolled, with 28 completing the study. The failure rate was as high as 30 percent. Similarly, the study conducted by Bergström et al. [27] exhibited a dropout rate of 15.7%. The follow-up population of less than 85% is considered to be a factor prone to bias. In the study by Artacho-Cordón. et al. [24], four participants did not complete the study due to excessive work commitments, with three in the intervention group and one in the control group affected by this. In the study by Lutfi et al. [29], all participants in the VR group completed the study. In contrast, one participant in the telehealth-delivered exercise group and two participants in the control group did not complete the study. Three participants did not complete the study by Carpenter et al. [28], and it was not specified whether they were in the intervention group or the control group.

### Effect of intervention on quality of life

Gonçalves et al. [25] found that, over the course of eight weeks of treatment, the quality of life (QoL) score of the yoga practitioners was significantly lower than that of the non-yoga practitioners, as measured by the EHP-30. Furthermore, the women in the yoga group exhibited enhanced quality of life in the domains of pain, control and powerlessness, emotional well-being, self-image, and social support. Notably, the control group was also provided with physiotherapy following the intervention at their institution.

Artacho-Cordón et al. [24] observed a more pronounced improvement in quality of life in the Physio-EndEA group compared to the control group at both the post-intervention and 1-year follow-up periods (large effect size(d>0.8)). The "Physio-EndEA" program is a nine-week, multimodal, supervised, and personalized therapeutic exercise program. The program has been shown to have a beneficial effect on the quality of life and pain experienced by women suffering from endometriosis. Notably, quality of life is also assessed through the EHP-30.

**Fig 4:**
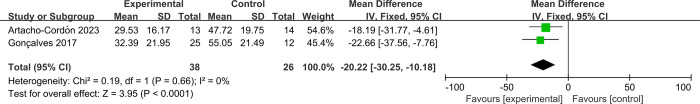
Forest plot of the analysis of the pain aspect of quality of life.

**Fig 5:**
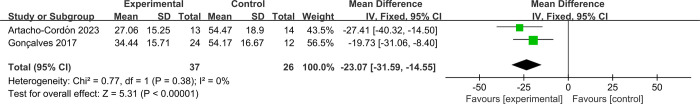
Forest plot of the analysis of the control and powerlessness aspect of quality of life.

**Fig 6:**
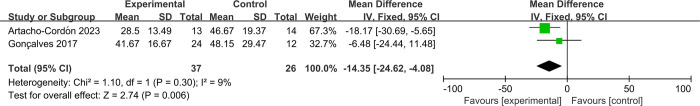
Forest plot of the analysis of the emotional well-being aspect of quality of life.

**Fig 7:**
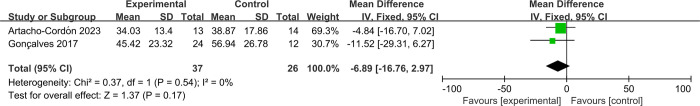
Forest plot of the analysis of the social support of quality of life.

**Fig 8:**
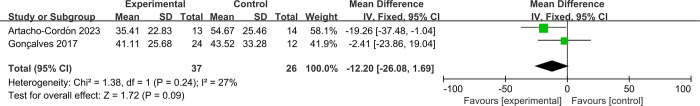
Forest plot of the analysis of the self-image aspects of quality of life.

Two studies used the EHP-30 to assess quality of life. A meta-analysis of two randomized controlled trials (71 subjects) comparing physical activity and exercise with a control group for improving quality of life was performed [24, 25]. The fix effects model was used in the meta-analysis. The results showed that after the physical activity and exercise intervention, there was a significant improvement in the pain aspect (WMD -20.22, 95% CI -30.25 to -10.18, P <0.0001, I^2^ = 0%, **Fig 4**), the control and powerlessness aspect(WMD -23.07, 95% CI -31.59 to -14.45, P <0.00001, I^2^ = 0%, **Fig 5**), and the emotional well-being aspect of quality of life compared with the control group (WMD -14.35, 95% CI -24.44 to -4.08, P = 0.006, I^2^ = 9%, **Fig 6**). Meanwhile, Zhao et al. [26] also reported that the progressive muscle relaxation (PMR) group exhibited a significantly greater improvement in overall/domain QOL than the control group (P < 0.05). However, health-related quality of life was measured using the SF-36 instrument. This is consistent with our findings. Furthermore, pooled results from the fixed-effects model indicated no significant differences in the social support (WMD-6.89, 95% CI -16.76 to 2.97, P = 0.17, I^2^ = 0%, **Fig 7**), and self-image aspects (WMD -12.20, 95% CI -26.08 to 1.69, P = 0.09, I^2^ = 27%, **Fig 8**) of QOL. However, the small number of patients included in the meta-analysis should be considered.

### Effect of intervention on pain

Lutfi et al. [29] reported no significant difference in the change in VAS scores between groups after the acute training intervention (p = 0.45).The telehealth (+10 ± 12 mm) and VR-delivered exercise (+9 ± 24 mm) interventions demonstrated a smaller increase in pain scores from the baseline compared to the control group (+16 ± 12 mm). However, there was also a "medium-large" group × time interaction effect on VAS scores (η2 = 0.10), suggesting that women with endometriosis had lower levels of pelvic pain after a single session of VR-delivered and telehealth-delivered exercise interventions compared with controls. This effect size is considered to be moderate to large. Notably, the sample size of the study was insufficient to permit the detection of statistical significance. The visual analog scale employed in this study constituted a modification of a previously validated VAS scale, no other study has employed this specific version of the VAS scale.

Gonçalves et al. [25] reported that daily pain levels were significantly lower in the yoga group than in the non-yoga group. Women in the yoga group had lower mean pain VAS scores, whereas the women in the non-yoga group exhibited a tendency to increase. In addition, after the intervention, scores on the pain-related domains of the EHP-30 were significantly lower in the yoga group than in the control group (32.39 ± 21.95 vs. 55.05 ± 21.49, p < 0.001).

In a study by Artacho-Cordón et al. [24], a near-significant reduction in current pelvic pain associated with "Physio-EndEA" was also observed after the intervention (-1.63 [-3.66, 0.02]; P = 0.060). Carpenter et al. [28] observed that pelvic pain was reduced in both the intervention and control groups. They found no additional effect on pelvic pain through physical activity and exercise compared to pharmacologic treatment with danazol. However, the study did not report the exact results and significance levels.

Zhao et al. [26] observed a significant improvement in quality of life concerning body pain in both the progressive muscle relaxation(PMR) and control groups. However, the PMR group exhibited more pronounced improvements than the control group (16.64 ± 21.73 vs 34.34 ± 18.47, p < 0.001). It is important to note that the study population was limited to patients who had been treated with gonadotrophin-releasing hormone (GnRH) agonists. The patients included in the study had previously failed contraceptive oral contraceptive (COC) therapy and therefore had stage III or stage IV endometriosis [30].

### Effects of intervention on mental health aspects

Zhao et al. [26] employed the SF-36 to assess mental health. The control group demonstrated no significant change in mental health before and after the intervention (p = 0.27). In contrast, the intervention group exhibited a significant improvement in mental health after the intervention (p = 0.04). A comparison of the improvement in scores between the two groups revealed that the PMR group exhibited a significantly greater improvement in scores in the mental health domains than the control group (p < 0.001).

Artacho-Cordón et al. [24] found that the intervention group exhibited notable improvements in emotional well-being as measured by the EHP-30, with a statistically significant difference (p < 0.05) compared to the control group. Furthermore, the findings at the one-year follow-up assessment indicated that the intervention group continued to demonstrate superior emotional well-being compared to the control group (p < 0.05). Gonçalves et al. [25] found significant improvements in certain EHP-30 items (emotional well-being) in the intervention group compared with the control group (p < 0.05).

### Effect of intervention on pelvic floor dysfunction

Gonçalves et al. [25] observed that the sexual intercourse domain of the EHP-30 was lower in both the intervention and control groups following eight weeks of hatha yoga. However, the results did not attain statistical significance at either the inter- or intra-group levels. Carpenter et al. [28] evaluated the impact of exercise during danazol therapy on pelvic floor symptoms, including painful intercourse and dysmenorrhea. The authors of this study reported improvements in symptoms in both groups but did not provide values or levels of significance. Artacho-Cordón et al. [24] observed a substantial effect size in dyspareunia following the intervention (d = 0.81; 95% CI -0.30 to 1.32).

### Effect of intervention on bone

A significant reduction in bone mineral density (BMD) of the femoral neck was observed in the control group relative to the physical training group following a 12-month intervention, as reported by Bergström et al. [27]. The intervention was unable to negate the bone loss caused by GnRH treatment during the initial six-month training period. Nevertheless, the application of physical exercise facilitated enhanced bone recovery in the femoral neck subsequent to the cessation of GnRH treatment, when compared to the control group. Six months after the cessation of GnRH treatment, no significant increase in spinal BMD was observed in the training group, although a slight increase was noted.

## Discussion

The relationship between physical activity and exercise and endometriosis has been the subject of extensive study in the past, with several reviews published on the topic [18–21, 31, 32]. However, the results of these reviews were inconclusive. The principal objective of this systematic review was to identify randomized controlled trials that investigate the effects of physical activity and exercise on endometriosis-related symptoms and to analyze and synthesize the available evidence. A total of six randomized controlled trials were identified, involving a total of 251 women diagnosed with endometriosis. The studies revealed that physical activity and exercise have a beneficial impact on quality of life, pain intensity, mental health, pelvic floor dysfunction, and bone density, in individuals diagnosed with endometriosis. However, only a simple meta-analysis of two studies could be performed due to the heterogeneity of the outcome measures and the incomplete reporting of the results in the studies included in this review [24, 25]. The systematic review and meta-analysis of quality of life demonstrated that physical activity and exercise had a significant impact on the improvement of three domains of quality of life: pain, control and powerlessness, and emotional well-being.

The long-term nature of endometriosis, along with its implications for emotional and mental health, has a significant impact on the overall quality of life of women diagnosed with the disease [33, 34]. A variety of clinical and surgical interventions have been employed with considerable success in patients with endometriosis [35]. Nevertheless, the biopsychosocial damage caused by the disease creates a vicious cycle that impacts the efficacy of underlying treatments, including clinical and surgical interventions [36]. Reducing the symptoms of endometriosis, particularly chronic pelvic pain, dysmenorrhea, and dyspareunia, is of paramount importance for enhancing the physical and mental health of individuals diagnosed with the disease. The implementation of holistic treatments may prove an effective strategy for enhancing quality of life.

The treatment of endometriosis presents a significant challenge [37]. Irrespective of the treatment received, approximately 50% of women with endometriosis will experience recurrent symptoms within five years [38]. Approximately 30% of women continue to experience pain due to endometriosis after primary treatment [39]. The pathogenesis of endometriosis pain is complex, including central sensitization (CS) and myofascial dysfunction. Moreover, it is noteworthy that pain symptoms may persist post-routine treatment [40]. Furthermore, the presence of CS has been observed in almost half of endometriosis patients, and such pain symptoms may be partially or completely unresponsive to routine endometriosis treatment [41]. Exercise therapy constitutes a significant component of evidence-based guidelines for chronic pain conditions [42]. A large study indicates that the integration of pain neuroscience education with cognition-targeted time-contingent exercise therapy results in a substantial improvement in self-reported symptoms of CS in patients suffering from chronic spinal pain [43]. Consequently, for women affected by endometriosis with CS, a multidisciplinary program combining centrally acting medications, pain education, pelvic floor physiotherapy, and behavioral health can help to improve pain symptoms and quality of life [44]. Furthermore, the mainstay of treatment for myofascial pelvic pain is a multimodal, multidisciplinary approach encompassing patient education, pelvic floor physiotherapy, and pharmacological injections at pelvic floor pain trigger points [45]. However, the potential adverse effects of pharmacologic and surgical interventions, in addition to the risk of recurrence and the poor effectiveness of conventional treatments, have prompted research into alternative treatment strategies. The inability of the majority of patients to travel to specialized treatment centers also underscores the necessity to develop alternative therapies that are effective in relieving or controlling pain [18, 46].

A multimodal approach that includes complementary therapy has been proposed as a potential method for alleviating symptoms associated with endometriosis [17, 47, 48]. However, the most efficacious complementary therapy for endometriosis-related symptoms remains to be determined [49]. A variety of approaches have been demonstrated to alleviate endometriosis symptoms and enhance quality of life, including antioxidant vitamins, dietary modifications, acupuncture, and psychological interventions [50–53]. Furthermore, several have suggested that exercise may be an option [54]. There is evidence to suggest that the symptoms associated with endometriosis are caused by a localized peritoneal inflammatory response resulting from the implantation of ectopic endometrium [55, 56]. The pain associated with endometriosis can be classified as either nociceptive (including inflammatory) or neuropathic, or a combination of both [57]. Prior research indicates that exercise may confer benefits and protection against chronic inflammatory diseases. Physical activity increases the systemic levels of various cytokines with anti-inflammatory properties [58, 59]. Furthermore, transverse and skeletal muscles are regarded as endocrine organs that stimulate the production and release of myocytokines through contraction, thereby potentially influencing and modifying the metabolism and cytokine production in tissues and organs. The contraction of these muscles releases myokines [60]. Moreover, exercise has been demonstrated to enhance the production of leukocytes, cortisol, and adrenaline, all of which have been shown to possess potent acute anti-inflammatory effects [61, 62]. It has been proposed that the underlying physiological mechanisms of the exercise-induced reduction in pro-inflammatory cytokines may include a reduction in the expression of genes involved in the regulation of the hypothalamic-pituitary-adrenal axis, as well as an improvement in the regulation of pain perception [63–65]. The aforementioned mechanisms demonstrate that physical activity and exercise serve to alleviate the symptoms of endometriosis.

Pain is a subjective experience, and endometriosis-related pain is a complex phenomenon that affects women in diverse ways and to varying degrees [66]. It is challenging to quantify and make comparisons between individuals who experience chronic pain. A uniform and accurate method of assessment is more conducive to our ability to assess the effects of physical activity and exercise on endometriosis-related pain. In our review, only two studies employed the use of the VAS to assess pain, while two others utilized the EHP-30, which is arguably the most pertinent questionnaire for evaluating pain in women with endometriosis. One study employed the SF-36 for the assessment of pain. Notably, other studies did not provide sufficient detail regarding the assessment of pain and the specific methods employed.

Endometriosis presents with a range of overlapping symptoms that are similar to those of other chronic inflammatory diseases, and the lack of reliable and reproducible diagnostic biomarkers makes it difficult to diagnose [67]. Furthermore, the presence of occult or diminutive lesions can compound the complexity of diagnosis. A definitive diagnosis based on laparoscopic surgery takes an average of 7–9 years [68]. Near-infrared radiation imaging after intravenous injection of indocyanine green imaging, employing two-dimensional or three-dimensional correlation, has been demonstrated to be valuable in identifying ’occult’ endometriotic lesions. However, the patients in this study were predominantly those with intermediate to advanced endometriosis [69]. It is noteworthy that only three studies in the present review diagnosed endometriosis on the basis of laparoscopic or open surgery, and the remaining three studies did not specify the basis of diagnosis. Further investigation is required to ascertain whether there are differences in the efficacy of physical activity and exercise in patients with varying degrees of endometriosis.

This systematic review is distinguished by its rigorous search strategy and methodological robustness. Additionally, the strength of our study lies in the inclusion of a greater number of studies for analysis, leading to more positive results compared to the previous review. Furthermore, all of the included studies were randomized controlled trials. However, it should be acknowledged that certain limitations are unavoidable. Firstly, the small sample sizes are small, which could potentially impact the reliability of the results. Secondly, there was considerable variation in the length of the intervention and follow-up periods across the studies. Furthermore, the included studies employed disparate exercise regimens, including regular exercise, yoga, and additional individualized or unspecified programs. Finally, a further issue was that all studies employed a wide range of outcome measures.

The findings of our comprehensive assessment indicate that PA and exercise may have a range of beneficial effects on endometriosis-related symptoms. It is recommended that healthcare professionals disseminate information regarding the benefits of physical activity to patients with endometriosis. Nevertheless, additional randomized controlled trials with superior methodological quality and longer follow-up periods are required to substantiate the impact of physical activity on patients with endometriosis. It is recommended that future studies adopt a patient-centered approach to measure and report relevant core outcomes from the patient’s perspective. These outcomes should include quality of life, improvement in pain and endometriosis symptoms, and acceptability and satisfaction scores. Furthermore, it is imperative that reliable and validated tools be employed to assess these outcomes. It is recommended that future studies concentrate on the type and frequency of physical activity and exercise, as well as the criteria for patient selection.

## Conclusion

The systematic review and meta-analysis demonstrated that physical activity and exercise had a significant impact on three domains of quality of life, including pain, control and powerlessness, and emotional well-being. The systematic review indicates that physical activity and exercise can alleviate pain, promote psychological well-being, and improve pelvic floor dysfunction. Moreover, physical activity and exercise have been demonstrated to facilitate bone reconstruction in women with endometriosis who have undergone treatment with GnRH analogs. However, more large sample sizes and well-designed randomized controlled trials are needed to provide evidence-based recommendations on physical activity for women with endometriosis-related symptoms.

## Supporting information

S1 File (DOCX)
