# Supplementary material for: The effectiveness and safety of physical activity and exercise on women with endometriosis: A systematic review and meta-analysis
Source: PLoS One. 2025 Feb 13;20(2):e0317820. doi: 10.1371/journal.pone.0317820 (PMC11824993; doi:10.1371/journal.pone.0317820)
Supplement: S1 File — (DOCX) [file pone.0317820.s001.docx]

# **Supporting1 File**

## **Search strategy**

**Table S1. Search strings and number of results**

| **Database**  **Date and Number of Results** | **Search**  **process** | **Search term** |
| --- | --- | --- |
| **Pubmed**  **08/05/2024**  **78** | #1 | (Endometriosis[Mesh] OR endometrio*[Title/Abstract]) |
|  | #2 | (Exercise[Mesh] OR "Physical Therapy Modalities"[mesh] OR "Exercise Movement Techniques"[Mesh] OR "Exercise Therapy"[mesh] OR Sports[mesh] OR "Resistance Training"[Mesh]) OR "High-Intensity Interval Training"[Mesh] OR (Physical Activity[Title/Abstract] OR exercis*[Title/Abstract] OR physical-activ*[Title/Abstract] OR sport*[Title/Abstract] OR physical-therap*[Title/Abstract] OR physiotherap*[Title/Abstract] OR physio-therap*[Title/Abstract] OR movement-therap*[Title/Abstract] OR motion-therap*[Title/Abstract]OR kinesiotherap*[Title/Abstract]OR kinesitherap*[Title/Abstract] OR "Acute Exercise"[Title/Abstract] OR "Acute Exercise*"[Title/Abstract] OR "Isometric Exercis*"[Title/Abstract] OR "Aerobic Exercis*"[Title/Abstract] OR "Exercise Training"[Title/Abstract] OR "Exercise Trainin*"[Title/Abstract] OR "Training, Exercise"[Title/Abstract] OR Athletic*[Title/Abstract] OR jog[Title/Abstract] OR jogging*[Title/Abstract] OR walk*[Title/Abstract] OR breathing-exercis*[Title/Abstract] OR yoga*[Title/Abstract] OR yogi*[Title/Abstract] OR pilates*[Title/Abstract] OR aerobic*[Title/Abstract] OR qigong*[Title/Abstract] OR qi-gong*[Title/Abstract] OR tai-ji*[Title/Abstract] OR taji*[Title/Abstract] OR tai-chi*[Title/Abstract] OR danc*[Title/Abstract] OR gymnast*[Title/Abstract] OR bicycl*[Title/Abstract] OR plyometric*[Title/Abstract] OR stretch*[Title/Abstract] OR swim*[Title/Abstract] OR workout*[Title/Abstract] OR work-out*[Title/Abstract]) |
|  | #3 | #1 AND #2 |
|  | #4 | (randomized controlled trial[Title/Abstract] OR trial*[Title/Abstract] OR random*[Title/Abstract] OR RCT[Title/Abstract]) |
|  | #5 | #3 AND #4 |
| **Embase**  **08/05/2024**  **172** | #1 | endometriosis:ti,ab,kw OR endometrio*:ti,ab,kw |
|  | #2 | 'physical activity'/exp OR 'exercise'/exp OR 'kinesiotherapy'/exp OR 'physiotherapy'/exp OR 'sport'/exp |
|  | #3 | exercis*:ti,ab,kw OR 'physical activ*':ti,ab,kw OR sport*:ti,ab,kw OR 'physical therap*':ti,ab,kw OR physiotherap*:ti,ab,kw OR 'physio therap*':ti,ab,kw OR 'movement therap*':ti,ab,kw OR 'motion therap*':ti,ab,kw OR kinesiotherap*:ti,ab,kw OR kinesitherap*:ti,ab,kw OR strength*:ti,ab,kw OR jog*:ti,ab,kw OR walk*:ti,ab,kw OR 'breathing exercis*':ti,ab,kw OR yoga*:ti,ab,kw OR yogi*:ti,ab,kw OR pilates*:ti,ab,kw OR aerobic*:ti,ab,kw OR qigong*:ti,ab,kw OR 'qi gong*':ti,ab,kw OR 'tai ji*':ti,ab,kw OR taji*:ti,ab,kw OR 'tai chi*':ti,ab,kw OR danc*:ti,ab,kw OR gymnast*:ti,ab,kw OR bicycl*:ti,ab,kw OR plyometric*:ti,ab,kw OR stretch*:ti,ab,kw OR swim*:ti,ab,kw OR workout*:ti,ab,kw OR 'work out*':ti,ab,kw |
|  | #4 | #2 OR #3 |
|  | #5 | #1 AND #4 |
|  | #6 | 'randomized controlled trial':ti,ab,kw OR trial*:ti,ab,kw OR random*:ti,ab,kw OR rct*:ti,ab,kw |
|  | #7 | #5 AND #6 |
| **The Cochrane Library**  **08/05/2024**  **82** | #1 | (Endometriosis OR endometrio*):ab,ti,kw |
|  | #2 | ("Exercise" OR "Physical Therapy Modalities" OR "Exercise Movement Techniques" OR "Exercise Therapy" OR "Sport" OR "Resistance Training" OR "High-Intensity Interval Training" OR "Physical Activity" exercis* OR "physical activ*" OR sport* OR "physical therap*" OR physiotherap* OR "physio therap*" OR "movement therap*" OR "motion therap*" OR kinesiotherap* OR kinesitherap* OR strength* OR jog OR jogging* OR walk* OR "breathing exercis*" OR yoga* OR yogi* OR pilates* OR aerobic* OR qigong* OR "qi gong*" OR "tai ji*" OR taji* OR "tai chi*" OR danc* OR gymnast* OR bicycl* OR plyometric* OR stretch* OR swim* OR workout*):ab,ti,kw |
|  | #3 | (randomized controlled trial or (trial* or random* or RCT*):ab,ti,kw |
|  | #4 | #1 AND #2 AND #3 |
| **Medline**  **08/05/2024**  **140** | #1 | Endometriosis or endometrio* |
|  | #2 | "Exercise" or "Physical Therapy Modalities" or "Exercise Movement Techniques" or "Exercise Therapy" or "Sport" or "Resistance Training" or "High-Intensity Interval Training" or "Physical Activity exercis*" or "physical activ*" or sport* or "physical therap*" or physiotherap* or "physio therap*" or "movement therap*" or "motion therap*" or kinesiotherap* or kinesitherap* or strength* or jog or jogging* or walk* or "breathing exercis*" or yoga* or yogi* or pilates* or aerobic* or qigong* or "qi gong*" or "tai ji*" or taji* or "tai chi*" or danc* or gymnast* or bicycl* or plyometric* or stretch* or swim* or workout* |
|  | #3 | randomized controlled trial or (trial* or random* or RCT* |
|  | #4 | #1 AND #2 AND #3 |
| **Web of Science**  **08/05/2024**  **92** | #1 | endometrio* |
|  | #2 | exercis* OR physical-activ* OR sport* OR physical-therap* OR physiotherap* OR physio-therap* OR movement-therap* OR motion-therap* OR kinesiotherap* OR kinesitherap* OR strength* OR jog OR jogging* OR walk* OR breathing-exercis* OR yoga* OR yogi* OR pilates* OR aerobic* OR qigong* OR qi-gong* OR tai-ji* OR taji* OR tai-chi* OR danc* OR gymnast* OR bicycl* OR plyometric* OR stretch* OR swim* OR workout* OR work-out* |
|  | #3 | #1 AND #2 |
| **Total records** | | **564** |
| **Total after removing duplicates** | | **309** |
| **Full-text articles evaluated for eligibility** | | **33** |
| **Studies included in quantitative synthesis** | | **6** |
| **Studies included in the meta-analysis** | | **2** |

**Identification**

Records identified through database searching (n=564)

Pubmed(n=78); The Cochrane Library(n=82); Embase(n=172); Medline:(n=140); Web of Science(n=92)

Records removed before screening: Duplicate records removed (n =255)

Records after removal of

duplicates (n=309)

**Screening**

Records excluded after reading the title and abstract

(n =276)

Full-text articles evaluated

for eligibility (n=33)

Exclude records(n=27)

-Not for randomized controlled trials(n=1)

-Review article(n=8)

-Intervention was not considered as physical activity or exercise (n=6)

-Not for Endometriosis(n=2)

-No available data (n=4)

-Uncompleted studies(n=6)

**Eligibility**

Studies included in quantitative synthesis (n =6)

Studies included in meta-analysis (n =2)

**Included**

**Fig 1. PRISMA flow diagram of the study process. PRISMA, Preferred Reporting Items for Systematic Review and Meta-analysis.**

**Table S2. The total number of initial screened articles was 564, 255 duplicates, and a total of 309 articles after deletion of duplicates. 276 articles were excluded and 33 articles were assessed in full text by reading the titles and abstracts of the articles to screen those that met the inclusion criteria, and the specific rationale for the exclusion is shown in the table below.**

| Reasons for exclude | Records |
| --- | --- |
| **Title and abstract not relevant to endometriosis** | **163** |
| menstrual cycles | Feasibility Study on Menstrual Cycles With Fitbit Device (FEMFIT): Prospective Observational Cohort Study[1] |
| endometrioid endometrial carcinoma | Disease progression, survival, and molecular disparities in Black and White patients with endometrioid endometrial carcinoma in real-world registries and GOG/NRG oncology randomized phase III clinical trials[2] |
| chronic pelvic pain | The role of psychosocial factors in the interprofessional management of women with chronic pelvic pain: A systematic review[3] |
| natural menopause age | Review of mendelian randomization studies on age at natural menopause[4] |
| prostatic cancer | Effects of gut microbiota on prostatic cancer: a two-sample Mendelian randomization study[5] |
| ovarian cancer | Causal effects of physical activity on the risk of overall ovarian cancer: A Mendelian randomization study[6] |
| endometrial cancer | Early detection, risk factors, and prevention of endometrial cancer[7] |
| ovarian cancer | 821TiP Phase I study of ceralasertib (cerala) in combination with AZD5305 in patients (pts) with advanced/metastatic ovarian cancer (OC) previously treated with PARP inhibitors (PARPis)[8] |
| ovarian cance | 747MO First results from the ENGOT-GYN2/GOG-3051/BOUQUET phase II biomarker-directed platform study: Cobimetinib (cobi) or atezolizumab (atezo) + bevacizumab (bev) for persistent/recurrent rare epithelial ovarian cancer (eOC)[9] |
| pelvic pain | Mindful Movement for Pelvic Pain[10] |
| ovarian tumor | p53 and ovarian carcinoma survival: an Ovarian Tumor Tissue Analysis consortium study[11] |
| pelvic pain | Are pelvic pain and increased pelvic floor muscle tone associated in women with persistent noncancer pelvic pain? A systematic review and meta-analysis[12] |
| ovarian cancer | LBA37 Atezolizumab (atezo) combined with platinum-based chemotherapy (CT) and maintenance niraparib for recurrent ovarian cancer (rOC) with a platinum-free interval (TFIp) >6 months: Primary analysis of the double-blind placebo (pbo)-controlled ENGOT-Ov41/GEICO 69-O/ANITA phase III trial[13] |
| patients with fresh embryo transfer | Impact of growth hormone on IVF/ICSI outcomes and endometrial receptivity of patients undergoing GnRH antagonist protocol with fresh embryo transfer: a pilot study[14] |
| 15 site-specific cancers | Genetic associations of leisure sedentary behaviors and the risk of 15 site-specific cancers: A Mendelian randomization study[15] |
| endometrial cancer | Molecular profiling of p53 mutant endometrial cancer reveals distinct subgroups with opportunities for personalized therapeutic approaches[16] |
| endometrial carcinoma | A randomized phase II trial of everolimus and letrozole or hormonal therapy in women with advanced, persistent or recurrent endometrial carcinoma: A GOG Foundation study[17] |
| dysmenorrhea | Transcutaneous electrical neurostimulation relieves primary dysmenorrhea: A randomized, double-blind clinical study versus placebo[18] |
| PFS | PRIMA/ENGOT-OV26/GOG-3012 study: Updated long-term PFS and safety[19] |
| ovarian cancer | Prognostic significance of ethnicity and age in advanced stage epithelial ovarian cancer: An NRG oncology/gynecologic oncology group study[20] |
| IVF patients | Great expectations of IVF patients: the role of gender, dispositional optimism and shared IVF prognoses[21] |
| ovarian cancer | Relacorilant + nab-paclitaxel in patients with recurrent, platinum-resistant ovarian cancer: Phase II subgroup analysis mirroring the patient population of an upcoming phase III study[22] |
| chronic pelvic pain | Efficacy of capacitive resistive monopolar radiofrequency in the physiotherapeutic treatment of chronic pelvic pain syndrome: A randomized controlled trial[23] |
| prolapse occurrence | pRophylactic utErosacral suspension AT Total lAparoscopiC Hysterectomy and the risk of prolapse occurrence – a randomised controlled trial (REATTACH)[24] |
| ovarian cancer | Delineating characteristic sequence and structural features of precursor and mature piwi-interacting rnas of epithelial ovarian cancer[25] |
| endometrial cancer | Phase II trial evaluating efficacy of a Fitbit program for improving the health of endometrial cancer survivors[26] |
| ovarian cancer | ENGOT-ov54/Swiss-GO-2/MATAO including LOGOS (Low-Grade Ovarian cancer Sub-study): MAintenance Therapy with Aromatase inhibitor in epithelial Ovarian cancer-A randomized, double-blinded, placebocontrolled, multicenter phase III Trial[27] |
| endometrial cancer | Fertility-Sparing Treatment for Atypical Endometrial Hyperplasia and Endometrial Cancer: A Cochrane Systematic Review Protocol[28] |
| endometrial cancer | A Surgical Window Trial Evaluating Medroxyprogesterone Acetate with or without Entinostat in Patients with Endometrial Cancer and Validation of Biomarkers of Cellular Response[29] |
| genital dyschromia | A Standardized Evaluation Method for Assessing Patients With Genital Dyschromia[30] |
| endometrioma | The impact of endometrioma on in vitro fertilisation/intra-cytoplasmic injection IVF/ICSI reproductive outcomes: a systematic review and meta-analysis[31] |
| ovarian cancer | Efficacy and safety of niraparib in older patients (pts) with advanced ovarian cancer (OC): Results from the PRIMA/ENGOT-OV26/GOG-3012 trial[32] |
| ovarian cancer | MOONSTONE/GOG-3032: A phase II, open-label, single-arm study to evaluate the efficacy and safety of niraparib + dostarlimab in patients with platinum-resistant ovarian cancer[33] |
| ovarian cancer | Impact of genetically predicted elevated concentrations of C-reactive protein on ovarian cancer risk: A Mendelian randomization study[34] |
| endometrial cancer | Comparative assessment of survival rate in endometrial cancer intermediate risk[35] |
| endometrial cancer | Reproducibility of lymphovascular space invasion (LVSI) assessment in endometrial cancer[36] |
| ovarian cancer | Metabolic syndrome and risk of ovarian cancer in the United States: An analysis of linked seer-medicare data[37] |
| endometrial cancer | A randomized controlled trial of WATAAP to promote physical activity in colorectal and endometrial cancer survivors[38] |
| ovarian cancer | Univariate and classification analysis reveals potential diagnostic biomarkers for early stage ovarian cancer Type 1 and Type 2[39] |
| intrinsic feedback | The Positive Effect of Intrinsic Feedback on Motivational Engagement and Self-Efficacy in Information Literacy[40] |
| ovarian cancer | Niraparib therapy in patients with newly diagnosed advanced ovarian cancer (PRIMA/ENGOT-OV26/GOG-3012 study)[41] |
| dyspareunia | Dyspareunia in Women: Updates in Mechanisms and Current/Novel Therapies[42] |
| endometrial cancer | Association between MDM2 SNP309 and endometrial cancer risk A PRISMA-compliant meta-analysis[43] |
| endometrial cancer | Short Course Vaginal Cuff Brachytherapy in Treating Participants With Stage I-II Endometrial Cancer[44] |
| ovarian cancer | The Effects of Exercise on Distress, Quality of Life, and Biomarkers in Ovarian Cancer Survivors[45] |
| pelvienne chronique | No 164 - Directive clinique de consensus pour la prise en charge de la douleur pelvienne chronique[46] |
| dysmenorrhoea | Effectiveness of dry needling of rectus abdominis trigger points for the treatment of primary dysmenorrhoea: a randomised parallel-group trial[47] |
| endometrial cancer | Surgical outcomes among elderly women with endometrial cancer treated by laparoscopic hysterectomy: a NRG/Gynecologic Oncology Group study[48] |
| endometrial cancer | Endometrial cancer: Epidemiology and etiology[49] |
| ovarian cancer | Identification of 12 new susceptibility loci for different histotypes of epithelial ovarian cancer[50] |
| ovarian cancer | Long term quality of life among epithelial ovarian cancer patients: The GINECO case/control VIVROVAIRE Study[51] |
| endometrial cancer | Effect of Total Laparoscopic Hysterectomy vs Total Abdominal Hysterectomy on Disease-Free Survival Among Women With Stage I Endometrial Cancer: A Randomized Clinical Trial[52] |
| male rat | Treadmill exercise attenuates 3,4-methylenedioxymethamphetamine-induced memory impairment through a decrease apoptosis in male rat hippocampus[53] |
| dysmenorrhea | No. 345-Primary Dysmenorrhea Consensus Guideline[54] |
| endometrial cancer | Pathologic and Treatment Outcomes Among a Geriatric Population of Endometrial Cancer Patients: An NRG Oncology/Gynecologic Oncology Group Ancillary Data Analysis of LAP2[55] |
| ovarian cancer | Does time interval between surgery and intraperitoneal chemotherapy administration in advanced ovarian cancer carry a prognostic impact? An NRG Oncology/Gynecologic Oncology Group study ancillary study[56] |
| uterine cancer | Impact of histology and surgical approach on survival among women with early-stage, high-grade uterine cancer: An NRG Oncology/Gynecologic Oncology Group ancillary analysis[57] |
| endometrial endometrioid adenocarcinoma | Expression Patterns of the Wnt Pathway Inhibitors Dickkopf3 and Secreted Frizzled-Related Proteins 1 and 4 in Endometrial Endometrioid Adenocarcinoma: An NRG Oncology/Gynecologic Oncology Group Study[58] |
| ovarian cancer | Intake of vitamins A, C, and E and folate and the risk of ovarian cancer in a pooled analysis of 10 cohort studies[59] |
| endometrial cancer | Associations between etiologic factors and mortality after endometrial cancer diagnosis: the NRG Oncology/Gynecologic Oncology Group 210 trial[60] |
| endometrioid endometrial adenocarcinoma | Histologic effects of medroxyprogesterone acetate on endometrioid endometrial adenocarcinoma: a Gynecologic Oncology Group study[61] |
| chronic pelvic pain | Complementary and alternative medications for chronic pelvic pain[62] |
| endometrial cancer | The use of technology-based weight loss intervention for endometrial cancer survivors with obesity[63] |
| endo- metrioid adenocarcinoma | Prospective lifestyle modification in patients with good prognosis endo- metrioid adenocarcinoma of the uterus[64] |
| endometrial cancer | Etiologic heterogeneity in endometrial cancer: evidence from a Gynecologic Oncology Group trial[65] |
| ovarian cancer | Tubal ligation, hysterectomy and ovarian cancer: A meta-analysis[66] |
| endometrial cancer | Targeted mutation analysis of endometrial cancer using a custom sequenom® massarray panel: A proof-of-principle study[67] |
| cesarean delivery | Elective repeat cesarean delivery compared with spontaneous trial of labor after a prior cesarean delivery: a propensity score analysis[68] |
| gynaecological endoscopic | Gynaecological endoscopic evaluation of 4% icodextrin solution: a European, multicentre, double-blind, randomized study of the efficacy and safety in the reduction of de novo adhesions after laparoscopic gynaecological surgery[69] |
| erhuang powder | Clinical observation of erhuang powder's promoting the wound healing of cervical columnar epithelium ectopy after physiotherapy[70] |
| ovarian cancer | Metabolic risk factors and ovarian cancer in the Metabolic Syndrome and Cancer project[71] |
| endometrial carcinoma | A phase 2 study of the oral mammalian target of rapamycin inhibitor, everolimus, in patients with recurrent endometrial carcinoma[72] |
| ovarian carcinoma | Diagnosis of ovarian carcinoma cell type is highly reproducible: A transcanadian study[73] |
| symptomatic adenomyosis | Uterine artery embolisation for symptomatic adenomyosis--mid-term results[74] |
| Ovarian, Fallopian Tube, or Primary Peritoneal Cancer | Diet and Physical Activity Change or Usual Care in Improving Progression-Free Survival in Patients With Previously Treated Stage II, III, or IV Ovarian, Fallopian Tube, or Primary Peritoneal Cancer[75] |
| laparoscopic hysterectomy | A randomized comparison of vesicourethral function after laparoscopic hysterectomy with and without vaginal cuff suspension[76] |
| endometrial cancer | Does fluid hysteroscopy increase the stage or worsen the prognosis in patients with endometrial cancer? A randomized controlled trial[77] |
| reproductive-age women | Complementary and alternative medicine (CAM) in reproductive-age women: a review of randomized controlled trials[78] |
| pelvic pain | Pelvic pain. A SAFE approach[79] |
| Uterin | Uterine ultrasonographic changes with gonadotropin-releasing hormone agonists[80] |
| hepatic venoocclusive disease | Hepatic venoocclusive disease as a complication of whole abdominopelvic irradiation and treatment with the transjuglar intrahepatic portosystemic shunt: case report and literature review[81] |
| IVF | Fertilization failure in IVF: Why and what next?[82] |
| ovarian cancers | Evaluation of the reproducibility of the World Health Organization classification of common ovarian cancers: With emphasis on methodology[83] |
| pelvic floor surgery | Pain Catastrophizing and Impact on Pelvic Floor Surgery Experience[84] |
| dysmenorrhea | Electrical Stimulation in women with Primary Dysmenorrhea[85] |
| human sperm selection | Human sperm selection using cumulus oophorus complexes compared with conventional sperm preparation method on sperm quality and ICSI outcomes: A pilot study[86] |
| cervical cancer | LBA38 Pembrolizumab plus chemoradiotherapy for high-risk locally advanced cervical cancer: A randomized, double-blind, phase III ENGOT-cx11/GOG-3047/KEYNOTE-A18 study[87] |
| adenomyosis | Guideline No. 437: Diagnosis and Management of Adenomyosis[88] |
| rajovriddhi | To see the effect of Rajonivrittikar Yoga in middle aged women with rajovriddhi (Heavy Bleeding during menses) in stopping menses: study on Cell line and Clinical Trial[89] |
| irisin for women | The Role of Irisin throughout Women's Life Span[90] |
| women | Guideline No. 425a: Cannabis Use Throughout Women's Lifespans - Part 1: Fertility, Contraception, Menopause, and Pelvic Pain[91] |
| unexplained infertility | Definitions and diagnostic criteria for unexplained infertility (UI) - a systematic review[92] |
| primary dysmenorrhea | Effects of Connective Tissue Manipulation Versus Stretching Exercises in Primary Dysmenorrhea[93] |
| ovarian reserve after surgery | Evaluating Ovarian Reserve After Conventional Laparoscopy Versus Robotic Surgery for Bilateral Endometrioma[94] |
| women with no known subfertility | Characteristics of menstrual cycles with or without intercourse in women with no known subfertility[95] |
| lipedema | ESL CONSENSUS DOCUMENT ON LIPEDEMA: PROPOSAL FOR DISCUSSION[96] |
| ovarian cancer | Modification of the Association Between Frequent Aspirin Use and Ovarian Cancer Risk: A Meta-Analysis Using Individual-Level Data From Two Ovarian Cancer Consortia[97] |
| birth weight | The Causal Evidence of Birth Weight and Female-Related Traits and Diseases: A Two-Sample Mendelian Randomization Analysis[98] |
| sexual dysfunctions | Redefining innovation in sexual medicine: patients and public involvement to inform patient-centered web-based interventions for female sexual dysfunctions[99] |
| chronic pelvic pain | ID:16315 Successful Treatment of Chronic Pelvic Pain With Botulinum Toxin Injections[100] |
| pelvic pain | Experience with nutraceutical supplements in the treatment of pelvic pain in gynaecology: case reports[101] |
| menstrual disorder | Analysis of survey on menstrual disorder among teenagers using Gaussian copula model with graphical lasso prior[102] |
| chronic pelvic pain | A Comprehensive Update of the Superior Hypogastric Block for the Management of Chronic Pelvic Pain[103] |
| markers | Markers of human endometrial hypoxia can be detected in vivo and ex vivo during physiological menstruation[104] |
| adenomyosis | An axonemal alteration in apical endometria of human adenomyosis[105] |
| unilaterally oophorectomized women | Could hormonal and follicular rearrangements explain timely menopause in unilaterally oophorectomized women?[106] |
| chronic pelvic pain | Music therapy in rehabilitation treatment for chronic pelvic pain[107] |
| chronic migraine | OnabotulinumtoxinA Is an Effective Treatment for Chronic Migraine in Patients With Comorbid Fibromyalgia[108] |
| deep dyspareunia | Deep Dyspareunia: Review of Pathophysiology and Proposed Future Research Priorities[109] |
| PCOS | Impact of a diagnosis of polycystic ovary syndrome on diet, physical activity and contraceptive use in young women: Findings from the Australian Longitudinal Study of Women's Health[110] |
| IVF patients | Sleep - an underrated fertility booster? A questionnaire survey on the pattern of sleep among IVF patients and their reproductive outcome[111] |
| pelvic pain | A prospective, single-centre, single-arm, open label study of the long term use of a gonadotropin releasing hormone agonist (Triptorelin SR, 11.25 mg) in combination with Tibolone add-back therapy in the management of chronic cyclical pelvic pain[112] |
| ovarian cancer | Appraising the role of previously reported risk factors in epithelial ovarian cancer risk: A Mendelian randomization analysis[113] |
| endometrial resection | 2887 Hysterectomy after Failed Endometrial Resection and Endometrial Ablation Techniques. Can We Work Out When It is Going to Fail?[114] |
| spontaneous coronary artery dissection | Spontaneous coronary artery dissection in Asian women with different clinical presentation, risk scenario and coronary angiographic evaluation: Multi centric analysis[115] |
| dysmenorrhea | The Efficacy of Two Types of Acupuncture on Autonomic Nervous Activity and Quality of Life in Women With Dysmenorrhea[116] |
| Top-Cited Journal Articles | A Bibliometric Analysis of Top-Cited Journal Articles in Obstetrics and Gynecology[117] |
| pelvic pain | Hydrotherapy for women with persistent pelvic pain[118] |
| dyspareunia | Prospective Cohort of Deep Dyspareunia in an Interdisciplinary Setting[119] |
| ovarian clear cell carcinoma | Genomics to immunotherapy of ovarian clear cell carcinoma: Unique opportunities for management[120] |
| pelvic girdle pain | Effect of Low Level Laser Therapy and Pelvic Stabilisation Exercises on Postpartum Pelvic Girdle Pain[121] |
| hysterectomy trial | Laparascopic TAP and Hysterectomy Trial #2[122] |
| visceral hyperalgesia | Epidural and sacral neuromodulation for visceral hyperalgesia-a case series[123] |
| pelvic pain | Pulsed Radiofrequency Ablation of Pudendal Nerve for Treatment of a Case of Refractory Pelvic Pain[124] |
| female fertility | Shedding new light on female fertility: The role of vitamin D[125] |
| ovulation | Plasma fatty acids and ovulation[126] |
| reproductive surgery | Robotic-assisted laparoscopy in reproductive surgery: a contemporary review[127] |
| IUI | Prognostic factors influencing IUI success[128] |
| endometriosis and breast cancer | Endometriosis and breast cancer: A survey of the epidemiological studies[129] |
| dysmenorrhea | Trial to Assess MK-8342B Treatment Efficacy-Safety in Dysmenorrhea[130] |
| pelvic pain | Anorectal and Pelvic Pain[131] |
| patients with an indication for vaginal hysterectomy | Laparoscopically assisted vaginal hysterectomy (LAVH) versus vaginal hysterectomy (VH) - a prospective, randomized, double-blind study in patients with an indication for vaginal hysterectomy[132] |
| ovarian cancer | Pre-diagnostic serum levels of inflammation markers and risk of ovarian cancer in the Prostate, Lung, Colorectal and Ovarian Cancer (PLCO) Screening Trial[133] |
| histone acetylation | Effects of psychological stress in histone acetylation[134] |
| pelvic pain | Non-surgical interventions for the management of chronic pelvic pain[135] |
| dintakeand time to conception | Is there anassociation between vitamin dintakeand time to conception? Data from the fast trial[136] |
| effects of inflammation and stress reaction | Additive effects of inflammation and stress reaction on Toll-like receptor 4-mediated growth of endometriotic stromal cells[137] |
| ovarian endometrioma | Pelvic pain in women with ovarian endometrioma is mostly associated with coexisting peritoneal lesions[138] |
| mind body medicine | A review of the current status of mind body medicine in obstetric and gynecology practice[139] |
| AHRQ | AHRQ Comparative Effectiveness Reviews[140] |
| chronic pelvic pain | Noncyclic Chronic Pelvic Pain Therapies for Women: Comparative Effectiveness[141] |
| female | The effects of oxidative stress on female reproduction: A review[142] |
| stress urinary incontinence | Effects of pelvic floor muscle exercises on quality of life out comes in women with stress urinary incontinence[143] |
| pelvic pain | The complex nature of chronic pelvic pain[144] |
| overweight and obese subfertile women | The LIFESTYLE study: costs and effects of a structured lifestyle program in overweight and obese subfertile women to reduce the need for fertility treatment and improve reproductive outcome. A randomised controlled trial[145] |
| gynecologic disorders | Measurement of electrical resistance of dermal-visceral zones as a diagnostic tool for gynecologic disorders[146] |
| pelvic floor tension myalgia | Addition of Pudendal Blocks to Pelvic Floor Physical Therapy for the Treatment of Pelvic Floor Tension Myalgia[147] |
| pelvic pain | Spinal cord stimulation is an effective treatment for the chronic intractable visceral pelvic pain[148] |
| infertility | Survey of Australasian clinicians' prior beliefs concerning lipiodol flushing as a treatment for infertility: a Bayesian study[149] |
| dysmenorrhoea | Management of dysmenorrhoea[150] |
| pelvic pain | Chronic pelvic pain: aetiology and therapy[151] |
| intrauterine insemination | Analysis of results of intrauterine insemination performed once or twice per stimulated cycle[152] |
| infertility | A review of the use of lipiodol flushing for unexplained infertility[153] |
| pelvic pain | Consensus Guidelines for the Management of Chronic Pelvic Pain[154] |
| female infertility | Treating female infertility and improving IVF pregnancy rates with a manual physical therapy technique[155] |
| menstrual migraine | Menstrual migraine[156] |
| women | Wyeth: The leader in women's health - Yesterday, today, and tomorrow[157] |
| Japanese women | Soy product intake and premenopausal hysterectomy in a follow-up study of Japanese women[158] |
| premenopausal | Factors related to variation in premenopausal bone mineral status: a health promotion approach[159] |
| intraperitoneal insemination | Direct intraperitoneal insemination - Clinical results and comparison between two methods of sperm preparation[160] |
| infertility | After the FLUSH trial: a prospective observational study of lipiodol flushing as an innovative treatment for unexplained and endometriosis-related infertility[161] |
| subfertility | The FLUSH trial--flushing with lipiodol for unexplained (and endometriosis-related) subfertility by hysterosalpingography: a randomized trial[162] |
| dysmenorrhea | Trial 1 to Assess MK-8342B Treatment Efficacy-Safety in Dysmenorrhea plus Extension[163] |
| **Other types of studies** | **31** |
| review | Endometriosis and related pelvic pain: Association with stress, anxiety and depressive symptoms[164] |
| cross-sectional qualitative research | Endometriosis and fertility: women's accounts of healthcare[165] |
| observational studies | Endometriosis-related inflammation and fertility[166] |
| observational studies | Observation on the Application of Benefiting Kidney and Strengthening Spleen Therapy in Reducing Side Effect of Mirena[167] |
| case report | Pelvic pain1[168] |
| case | Two cases of hymenal stenosis in adult women[169] |
| case | Chiropractic spinal manipulative treatment of migraine headache of 40-year duration using Gonstead method: a case study[170] |
| review; information | High-Quality eHealth Websites for Information on Endometriosis: Systematic Search[171] |
| cohort study | Depression in Adolescent and Adult Women with Endometriosis[172] |
| mendelian randomization analysis | Assessing the relationship between gut microbiota and endometriosis: a bidirectional two-sample mendelian randomization analysis[173] |
| case-control study | Dysmenorrhea pattern in adolescences informing adult endometriosis[174] |
| case-control study | Folate Intake and Ovarian Cancer Risk among Women with Endometriosis: A Case-Control Study from the Ovarian Cancer Association Consortium[175] |
| observational study | Quality of Life in Japanese Patients with Dysmenorrhea or Endometriosis-Associated Pelvic Pain Treated with Extended Regimen Ethinylestradiol/Drospirenone in a Real-World Setting: A Prospective Observational Study[176] |
| review; diet on pain perception | Impact of diet on pain perception in women with endometriosis: A systematic review[177] |
| pain intensity | THERAPEUTIC EXERCISE AND EDUCATION IN PAIN NEUROPHYSIOLOGY TO IMPROVE PAIN INTENSITY IN WOMEN WITH ENDOMETRIOSIS. CLINICAL TRIAL[178] |
| review; lifestyle and diet | Impact of lifestyle and diet on endometriosis: a fresh look to a busy corner[179] |
| observational studies | Environmental Risk Factors for Endometriosis: An Umbrella Review of a Meta-Analysis of 354 Observational Studies With Over 5 Million Populations[180] |
| clinical conundrum | Clinical Conundrum: A 33-Year-Old With Pain Post-Orgasm and a History of Endometriosis[181] |
| cohort study | Botulinum toxin for chronic pelvic pain in women with endometriosis: A cohort study of a pain-focused treatment[182] |
| qualitative study | A qualitative study of patient and clinician perspectives on item importance, scoring preferences, and clinically important differences for two patient-reported outcome measures: Endometriosis Symptom Diary (ESD) and Endometriosis Impact Scale (EIS)[183] |
| review; infertility | Interventions for endometriosis related infertility: A, systematic review and network meta-analysis[184] |
| case-control study | 'Behind blue eyes'†: The association between eye colour and deep in filtrating endometriosis[185] |
| case-controlled study | Intra-uterine microbial colonization and occurrence of endometritis in women with endometriosis†[186] |
| case-controlled study | Role of prostaglandin E2 in bacterial growth in women with endometriosis[187] |
| review; GnRH-a | Gonadotrophin-releasing hormone analogues for endometriosis: bone mineral density[188] |
| case-control study | A case-control epidemiologic study of endometriosis[189] |
| case-control study | Menstrual status as risk factors of endometriosis: a case-control study[190] |
| post hoc analysis of pooled data | Clinically Meaningful Reduction in Dyspareunia Is Associated With Significant Improvements in Health-Related Quality of Life Among Women With Moderate to Severe Pain Associated With Endometriosis: A Pooled Analysis of Two Phase III Trials of Elagolix[191] |
| review; endometriosis risk | Physical activity and endometriosis risk in women with infertility or pain: Systematic review and meta-analysis[192] |
| validation study; THIN | Under-recording of endometriosis in the health improvement network (THIN) primary care database: A validation study[193] |
| questionnaire study | Healthcare Consumption and Cost Estimates concerning Swedish Women with Endometriosis[194] |
| **Other treatments** | **51** |
| Intrathecal drug delivery | Discovery of kinked medtronic ascenda intrathecal catheter[195] |
| surgical management | Identifying the Problems of Randomized Controlled Trials for the Surgical Management of Endometriosis-associated Pelvic Pain[196] |
| aromatase inhibitor plus progestin | Psychometric validation of the Endometriosis Symptom Diary (ESD) and Endometriosis Impact Scale (EIS): Findings from an interventional study[197] |
| surgery | A Prospective Study of Bladder Function Following Endometriosis Surgery With Up to Eight years Follow-up[198] |
| therapeutic-educational Physiotherapy | Therapeutic-educational Physiotherapy on Pain, Physical-functionality and Quality of Life in Women With Endometriosis[199] |
| transcranial magnetic stimulation | Is Repetitive Transcranial Magnetic Stimulation Effective in Reducing Endometriosis-associated Pain[200] |
| robotic management | Robotic Management of Recurrent Rectal Endometriosis After Previous Segmental Bowel Resection[201] |
| dietary interventions | The effect of dietary interventions on pain and quality of life in women diagnosed with endometriosis: a prospective study with control group[202] |
| surgery | Effects of sensate focus technique and position changing on sexual function of women with deep-infiltrating endometriosis after surgery: A clinical trial study[203] |
| physiotherapy based on tele-rehabilitation | Multimodal Physiotherapy Based on Tele-rehabilitation in Chronic Pelvic Pain Associated With Endometriosis[204] |
| self-management strategies | The Efficacy of Self-Management Strategies for Females with Endometriosis: a Systematic Review[205] |
| vitamin D | A systematic review of vitamin D and endometriosis: role in pathophysiology, diagnosis, treatment, and prevention[206] |
| psychological interventions | Psychological interventions improve quality of life despite persistent pain in endometriosis: results of a 3-armed randomized controlled trial[207] |
| ozone therapy | Effectiveness of Ozone Therapy Treatment of Endometriosis[208] |
| physical therapy; review | Benefits of physical therapy in improving quality of life and pain associated with endometriosis: A systematic review and meta-analysis[209] |
| medical treatment | The co-effect of sensate focus technique and sexual position changing on sexual function of women who use medical treatment for endometriosis[210] |
| conservative surgery | Effect of Postoperative Hormonal Suppression on Fertility in Patients With Endometriosis After Conservative Surgery: A Systematic Review and Meta-analysis[211] |
| surgery | Conventional Laparoscopy Versus Robotic Surgery for Pain Relief in Patients With Deep Infiltrating Endometriosis[212] |
| relugolix combination therapy | SPIRIT long-term extension study: two-year efficacy and safety of relugolix combination therapy in women with endometriosis-associated pain[213] |
| pelvic floor muscle exercises | Pelvic floor muscle exercises and mindfulness for women with endometriosis-associated pelvic pain[214] |
| medicinal cannabis | EndoCannED – The effect of Medicinal Cannabis on emergency department presentations in people with endometriosis: a randomised, controlled feasibility study[215] |
| leuprolide acetate | Analysis of the clinical efficacy of leuprolide acetate in the treatment of obese patients with endometriosis and its role on the expression of MIF gene[216] |
| laparoscopic treatment | Laparoscopic treatment of isolated superficial peritoneal endometriosis for managing chronic pelvic pain in women: study protocol for a randomised controlled feasibility trial (ESPriT1)[217] |
| hormonal therapy | Hormonal therapy potentiates the effect of surgery on gene expression profile of peripheral blood mononuclear cells in patients affected by endometriosis[218] |
| suppressive treatment | The Effect of Post-Operative Suppressive Treatment in Women with Endometriosis-Related Infertility[219] |
| complementary medicine | Overview of the Effect of Complementary Medicine on Treating or Mitigating the Risk of Endometriosis[220] |
| LNG-IUD | Levonorgestrel-releasing intrauterine device (LNG-IUD) for symptomatic endometriosis following surgery[221] |
| 3D/4D transperineal ultrasound | Assessment of levator hiatal area using 3D/4D transperineal ultrasound in women with deep infiltrating endometriosis and superficial dyspareunia treated with pelvic floor muscle physiotherapy: randomized controlled trial[222] |
| neuromuscular electrical stimulation | Effectiveness of neuromuscular electrical stimulation for endometriosis-related pain: A protocol of systematic review and meta-analysis[223] |
| hormonal treatment | Hormonal treatment isolated versus hormonal treatment associated with electrotherapy for pelvic pain control in deep endometriosis: Randomized clinical trial[224] |
| maintenance treatment | A prospective randomized controlled study of postoperative maintenance treatment for patients with endometriosis[225] |
| acupuncture | The Relationship of Acupuncture Use to the Endometriosis Risk in Females With Rheumatoid Arthritis: Real-World Evidence From Population-Based Health Claims[226] |
| review; self-management | Self-management in condition-specific health: A systematic review of the evidence among women diagnosed with endometriosis[227] |
| psychological and mind-body interventions | Psychological and mind-body interventions for endometriosis: A systematic review[228] |
| traditional Chinese patent medicine | Comparative efficacy and safety of traditional Chinese patent medicine for endometriosis: A Bayesian network meta-analysis protocol[229] |
| pulsedhigh-intensity laser | Effect of Pulsed High-Intensity Laser Therapy on Pain, Adhesions, and Quality of Life in Women Having Endometriosis: A Randomized Controlled Trial[230] |
| physiotherapy | Physiotherapy of the Pelvic Floor in Women With Deep Infiltrating Endometriosis[231] |
| rehabilitation program | Effect of a Rehabilitation Program to Improve Quality of Life in Women Diagnosed With Endometriosis (Physio-EndEA Study)[232] |
| danchi decoction | Effects of danchi decoction on P450arom, survivin of eutopic endometrium of patients with endometriosis after conservative surgery[233] |
| hormonal add-back therapy | The effect of hormonal add-back therapy in adolescents treated with a gonadotropin releasing hormone (GNRH) agonist for endometriosis: A randomized trial[234] |
| resveratrol | Is resveratrol a potential substitute for leuprolide acetate in experimental endometriosis?[235] |
| surgical versus low-dose progestin treatment | Surgical versus low-dose progestin treatment for endometriosis-associated severe deep dyspareunia II: Effect on sexual functioning, psychological status and health-related quality of life[236] |
| LNG-IUD | Postoperative application of LNG-IUD for symptomatic endometriosis[237] |
| LNG-IUD | Levonorgestrel-releasing intrauterine device (LNG-IUD) for symptomatic endometriosis following surgery[238] |
| surgical versus medical treatment | Surgical versus medical treatment for endometriosis-associated severe deep dyspareunia: I. Effect on pain during intercourse and patient satisfaction[239] |
| complementary and alternative medicine | Complementary and alternative medicine (CAM) therapies for management of pain related to endometriosis[240] |
| Surgery | Surgery for endometriosis related pain[241] |
| manual physical therapy | Decreasing dyspareunia and dysmenorrhea in women with endometriosis via a manual physical therapy: Results from two independent studies[242] |
| ear-electroacupuncture treatment | Randomized controlled study on ear-electroacupuncture treatment of endometriosis-induced dysmenorrhea in patients[243] |
| aromatase inhibitors; review | Systematic review of the effects of aromatase inhibitors on pain associated with endometriosis[244] |
| hypothetical comparisons between a new drug and a standard drug, and between medical and surgical treatment | "You can't always get what you want": from doctrine to practicability of study designs for clinical investigation in endometriosis[245] |
| **Biomarkers or gene** | **11** |
| biomarkers | Machine learning-based integrated identification of predictive combined diagnostic biomarkers for endometriosis[246] |
| transcriptomic landscape | The mid-secretory endometrial transcriptomic landscape in endometriosis: a meta-analysis[247] |
| IGF1R variant | Reduced cardiovascular risks in women with endometriosis or polycystic ovary syndrome carrying a common functional IGF1R variant[248] |
| biomarkers | Validated biomarker assays confirm that ARID1A loss is confounded with MMR deficiency, CD8+ TIL infiltration, and provides no independent prognostic value in endometriosis-associated ovarian carcinomas[249] |
| lncRNAs | Identification of Functional lncRNAs Associated With Ovarian Endometriosis Based on a ceRNA Network[250] |
| TNF-α gene | Association of TNF-α gene T-1031C polymorphism with endometriosis: A meta-analysis[251] |
| OCT4, SOX15 and TWIST1 | Enhanced expression of the stemness-related factors OCT4, SOX15 and TWIST1 in ectopic endometrium of endometriosis patients[252] |
| matrix metalloproteinase-2 and matrix metalloproteinase-9 polymorphisms | Association between matrix metalloproteinase-2 and matrix metalloproteinase-9 polymorphisms and endometriosis: A systematic review and meta-analysis[253] |
| tumor necrosis factor gene | Tumor necrosis factor gene polymorphisms and endometriosis in Asians: A systematic review and meta-analysis[254] |
| TGF-β1-509C/T polymorphism | Association between TGF-β1-509C/T polymorphism and endometriosis: A systematic review and meta-analysis[255] |
| expression of aromatase | Expression of aromatase (P450 aromatase/CYP19) in peritoneal and ovarian endometriotic tissues and deep endometriotic (adenomyotic) nodules of the rectovaginal septum[256] |
| **Guidelines; scale; consensus; perspective; and editorial** | **7** |
| guidelines | Development of Provisional Acupuncture Guidelines for Pelvic Pain in Endometriosis Using an e-Delphi Consensus Process[257] |
| scale | Patients' and clinicians' perspectives on item importance, scoring, and clinically meaningful differences for the Endometriosis Symptom Diary (ESD) and Endometriosis Impact Scale (EIS)[258] |
| consensus | World Endometriosis Society consensus on the classification of endometriosis[259] |
| consensus | Consensus on Recording Deep Endometriosis Surgery: the CORDES statement[260] |
| consensus | Russian consensus in treatment of endometriosis[261] |
| perspective | Management of adolescent chronic pelvic pain from endometriosis: A pain center perspective[262] |
| editorial | Towards comprehensive management of symptomatic endometriosis: beyond the dichotomy of medical versus surgical treatment[263] |
| **Animal models** | **5** |
| animal models | Palmitoylethanolamide effectiveness and safety for pain relief in animal models: A systematic review[264] |
| rats | Effects of the LHRH antagonist Cetrorelix on affective and cognitive functions in rats[265] |
| mice | Effects of the LHRH antagonist Cetrorelix on the brain function in mice[266] |
| mouse | Enriched Environment Decelerates the Development of Endometriosis in Mouse[267] |
| rats | Effect of a period of swimming exercise and vitamin E intake on catalase and superoxide dismutase activity, and malondialdehyde levels in ovarian tissue of endometriosis model rats[268] |
| **Reply to articles** | **3** |
| reply | Re: Hormonal treatment isolated versus hormonal treatment associated with electrotherapy for pelvic pain control in deep endometriosis: Randomized clinical trial[269] |
| reply | Re: Assessment of levator hiatal area using 3D/4D transperineal ultrasound in women with deep infiltrating endometriosis and superficial dyspareunia treated with pelvic floor muscle physiotherapy: randomized controlled trial[270] |
| reply | Reply to Letter to the Editor entitled "Re: Hormonal treatment isolated versus hormonal treatment associated with electrotherapy for pelvic pain control in deep endometriosis: Randomized clinical trial"[271] |
| **Retracted articles** | **2** |
| retracted | Effectiveness of complementary pain treatment for women with deep endometriosis through Transcutaneous Electrical Nerve Stimulation (TENS): randomized controlled trial[272] |
| retracted | RETRACTED: Effectiveness of complementary pain treatment for women with deep endometriosis through Transcutaneous Electrical Nerve Stimulation (TENS): randomized controlled trial (Retracted Article)[273] |
| **Other reasons** | **3** |
| the crescendo program | The CRESCENDO Program (in Crease Physical Exercise and Sport to Combat Endometriosis)[274] |
| etiology and epidemiology | Etiology and epidemiology of endometriosis[275] |
| neonatal uterine bleeding | Is neonatal uterine bleeding responsible for early-onset endometriosis?[276] |

**Table S3.** **A total of 33 articles were subjected to comprehensive evaluation. However, 27 were subsequently excluded following the stipulated inclusion and exclusion criteria, the specific rationales for which are delineated in the following table.**

| Reasons for exclude | Records |
| --- | --- |
| **Not for randomized controlled trials** | **1** |
| Yoga, but a qualitative study | A Qualitative Study on the Practice of Yoga for Women with Pain-Associated Endometriosis[277] |
| **Review article** | **8** |
| physical activity, but a review | Diet, physical activity, and complementary medicine: What else is beneficial? A review of current medical trials[278] |
| physical exercises, but a review | Endometriosis and physical exercises: a narrative review[279] |
| physical activity, but a review | What is the evidence of effectiveness of non-pharmaceutical, non-surgical, biopsychosocial interventions for body image and pain management in individuals with endometriosis? A systematic review[280] |
| exercise, but a review | Impact of exercise on pain perception in women with endometriosis: A systematic review[281] |
| physical activity and exercise, but a review | Effect of physical activity and exercise on endometriosis-associated symptoms: a systematic review[282] |
| physical activity, but a review | Endometriosis and physical activity: A systematic review[283] |
| physiotherapy treatment, but a review | Update on physiotherapy treatment in endometriosis: A systematic review[284] |
| complementary treatments, but a review | Systematic review and meta-analysis of complementary treatments for women with symptomatic endometriosis[285] |
| **Intervention was not considered as physical activity or exercise** | **6** |
| osteopathic manipulative therapy | Impact of osteopathic manipulative therapy in patient with deep with colorectal endometriosis: A classification based on symptoms and quality of life[286] |
| osteopathic manipulative therapy | Impact of osteopathic manipulative therapy on quality of life of patients with deep infiltrating endometriosis with colorectal involvement: results of a pilot study[287] |
| pelvic floor muscle physiotherapy | Effects of Pelvic Floor Muscle Physiotherapy on Urinary, Bowel, and Sexual Functions in Women with Deep Infiltrating Endometriosis: A Randomized Controlled Trial[288] |
| immersive digital therapeutic | Pain Reduction With an Immersive Digital Therapeutic in Women Living With Endometriosis-Related Pelvic Pain: At-Home Self-Administered Randomized Controlled Trial[289] |
| manual therapy protocol | Effectiveness of a Manual Therapy Protocol in Women with Pelvic Pain Due to Endometriosis: A Randomized Clinical Trial[290] |
| an adapted environmental enrichment intervention | Feasibility and acceptability of an adapted environmental enrichment intervention for endometriosis: A pilot study[291] |
| **Not for Endometriosis** | **2** |
| pelvic pain (with/without endometriosis) | Does group physiotherapy improve pain scores and reduce the impact of pelvic pain for women referred with persistent pelvic pain? A clinical trial[292] |
| women with chronic pelvic pain | Physical activity in women with chronic pelvic pain in a multidisciplinary clinic[293] |
| **No available data(Study Protocol)** | **4** |
| No available data(Study Protocol) | Effects of a physical activity and endometriosis-based education program delivered by videoconference on endometriosis symptoms: the CRESCENDO program (inCRease physical Exercise and Sport to Combat ENDOmetriosis) protocol study[294] |
|  | Yoga, cognitive-behavioural therapy versus education to improve quality of life and reduce healthcare costs in people with endometriosis: a randomised controlled trial[295] |
|  | Physiotherapeutic Exercises in Perineal Pain in Women with Endometriosis[296] |
|  | 'Physio-EndEA' Study: A Randomized, Parallel-Group Controlled Trial to Evaluate the Effect of a Supervised and Adapted Therapeutic Exercise Program to Improve Quality of Life in Symptomatic Women Diagnosed with Endometriosis[297] |
| **Uncompleted studies** | **6** |
| research registration information | Evaluating the influence of Yoga, Cognitive Behaviour Therapy and Standard Care on Quality of Life and Healthcare Costs in Endometriosis[298] |
| research registration information | Effects of Aerobic Exercise and Core Muscle Strengthening on Pain, Menstrual Pattern and QOL in Endometriosis[299] |
| research registration information | Effect of Physical Activity and Pain Education on Endometriosis-associated Pain[300] |
| research registration information | Effect of Mediterranean Diet and Physical Activity in Patients With Endometriosis[301] |
| research registration information | Yoga in the management of pain and related stress on women with Endometriosis[302] |
| research registration information | Iyengar Yoga Therapy for Dysmenorrhea and Endometriosis[303] |

**Table S4. The literature was screened according to the inclusion and exclusion criteria and the PICOS principle. A total of six studies met the inclusion criteria.**

| Study | **Participants:**  endometriosis | **Intervention**: physical activity or exercise | **Control**: Activities of daily living and/or routine treatment | **Outcome**: quality of life and other outcomes | **Study design**: RCT | **English literature** |
| --- | --- | --- | --- | --- | --- | --- |
| Artacho-Cordón. et al.[304] | √ | √ | √ | √ | √ | √ |
| Gonçalves. et al.[305] | √ | √ | √ | √ | √ | √ |
| Lutfi et al.[306] | √ | √ | √ | √ | √ | √ |
| Carpenter et al.[307] | √ | √ | √ | √ | √ | √ |
| Bergström et al.[308] | √ | √ | √ | √ | √ | √ |
| Zhao et al.[309] | √ | √ | √ | √ | √ | √ |

**Table S5. A total of two studies were subjected to meta-analysis, all the data extracted from each study are presented in the table below.**

| Study | EHP-30 pain | | Control and powerlessness | | Emotional well-being | | Social support | | Self-image | | Dropouts |
| --- | --- | --- | --- | --- | --- | --- | --- | --- | --- | --- | --- |
|  | **Intervention** | **Control** | **Intervention** | **Control** | **Intervention** | **Control** | **Intervention** | **Control** | **Intervention** | **Control** | Dropouts |
| Artacho-Cordón. et al.[304] | 29.53±16.17  (n=13) | 47.72±19.75  (n=14) | 27.06±15.25  (n=13) | 54.47±18.90  (n=14) | 28.50±13.49  (n=13) | 46.67±19.37  (n=14) | 34.03±13.40  (n=13) | 38.87±17.86  (n=14) | 35.41±22.83  (n=13) | 54.67±25.46  (n=14) | 4(12.90%)  (n=31) |
| Gonçalves. et al.[305] | 32.39±21.95  (n=25) | 55.05±21.49  (n=12) | 34.44±15.71  (n=24) | 54.17±16.67  (n=12) | 41.67±15.67  (n=24) | 48.15±29.47  (n=12) | 45.42±23.32  (n=24) | 56.94±26.78  (n=12) | 41.11±25.68  (n=24) | 43.52±33.28  (n=12) | 12 (30.00%)  (n=40) |
| Lutfi et al.[306] | +9±24 mm | +16±12 mm |  |  |  |  |  |  |  |  | 3(13.64%)  (n=22) |
| Carpenter et al.[307] |  |  |  |  |  |  |  |  |  |  | 3 (7.69%)  (n=39) |
| Bergström et al.[308] |  |  |  |  |  |  |  |  |  |  | 3(15.70%)  (n=19) |
| Zhao et al.[309] |  |  |  |  |  |  |  |  |  |  | 13(13.00%)  (n=100) |

NOTE: Data are shown as mean ± standard deviation.

**Table 1 Characteristics of the included studies.**

| Study | Country | Year | Recruitment | Number | Study population | Intervention description | Control group | Duration | primary outcomes | Secondary outcome | Dropouts |
| --- | --- | --- | --- | --- | --- | --- | --- | --- | --- | --- | --- |
| Artacho-Cordón. et al.[304] | **Spain** | **2023** | **2020-2021** | **31（16 interventions vs 15 controls）** | **endometriosis**  **unresponsive to conventional therapy** | **1-week lumbopelvic stabilization learning phase 8-week phase of**  **stretching, aerobic, and resistance exercises focused on the lumbopelvic sequentially instructed and supervised by a trained physiotherapist** | **usual treatments** | **9 weeks and 1-year follow-up** | **QOL(EHP-30)** | **PPTs, pain intensity, and pain-related catastrophizing thoughts, isometric resistance of abdominal and lumbar muscles, lumbopelvic stability, and muscle architecture of the abdominal wall and lumbar multifidus** | **4(12.9%),3 in the intervention group, 1 in the control group** |
| Gonçalves. et al.[305] | **Brazil** | **2017** | **2013.08-2014.12** | **40 (28 interventions vs 12**  **controls)** | **Endometriosis**  **and CPP, prior**  **hormonal and surgical therapy, age34.88 ± 6.70 years, no regular exercise** | **Supervised;120 min of Hatha yoga, posture**  **(60 min) + conversation**  **(30 min) + relaxation, breathing exercises, meditation (30 min) Medical therapy was continued** | **Continuing medical**  **therapy or**  **physiotherapy**  **once per week** | **Twice weekly**  **for 8 weeks** | **QOL (EHP-30)** | **Pain (VAS,0–10), menstrual pattern measured daily (amount of bleeding scored from 0 to 5)** | **12 (30%), only**  **in intervention**  **group** |
| Lutfi et al.[306] | **Australia** | **2023** | **2021.01-2022.09** | **22 (8 telehealth-delivered exercises; 8 VR-delivered exercises; 6 controls.)** | **Endometriosis patients without contraindications to exercise and visual impairment, age 18-45years** | **attend one supervised exercise or telehealth-delivered exercise training session** | **activities of daily living** | **48 hours** | **Pain (VAS,0-10)** | **NR** | **3(13.6%),1 in the telehealth-delivered exercise group, 2 in the control group** |
| Carpenter et al.[307] | **USA** | **1995** | **NR** | **39 (18 interventions vs 18**  **controls)** | **Endometriosis**  **with no other hormonal treatment**  **during previous**  **12 months, no**  **regular exercise** | **Unsupervised; 40 min of individualized cardio fitness at 50–70% of max heart rate + flexibility exercises + danazol** | **Danazol treatment only** | **Four times**  **weekly for**  **24 weeks** | **Number of**  **side effects**  **of danazol**  **(direct inquiry)** | **Fitness (VO2max),**  **general muscle strength**  **(KINCOM), sex hormone**  **levels, pelvic symptoms** | **3 (7.69%)** |
| Bergström et al.[308] | **Sweden** | **2005** | **NR** | **19(8 interventions vs 11**  **controls)** | **endometriosis,**  **no GnRH analogs, no other diseases or medications that**  **could interfere with the BMD. age 23–38 years** | **three 30-minute fast walks and two 1-h aerobic training sessions a week + GnRH treatment** | **activities of daily living + GnRH treatment** | **Every six months for 12 months** | **BMD** | **NR** | **3(15.7%),2 in the intervention group, 1 in the control group** |
| Zhao et al.[309] | **China** | **2012** | **2010.01-2010.11** | **100(50 interventions vs 50**  **Controls)** | **Chinese endometriosis, age 18–48 years** | **twenty-four 40-min group PMR practice sessions over 12 weeks, twice per week. +depot leuprolide** | **activities of daily living +depot leuprolide** | **12weeks** | **QOL(SF-36)** | **anxiety, depression** | **13(13%), 8 in the intervention group, 5 in the control group** |

**QOL** quality of life, **PPTs** pressure pain thresholds, **CPP** chronic pelvic pain, **VAS** visual analog scale, **KINCOM** Kinetic Communicator Exercise System, **BMD** bone mineral density, **SF-36** 36-item Short-Form Health Survey, **EHP-30** Endometriosis Health Profile-30, **PMR** progressive muscular relaxation, **GnRH** gonadotrophin-releasing hormone, **VR** virtual reality, **NR** not reported.

**Fig 2. Overall risk of bias assessment.**

**
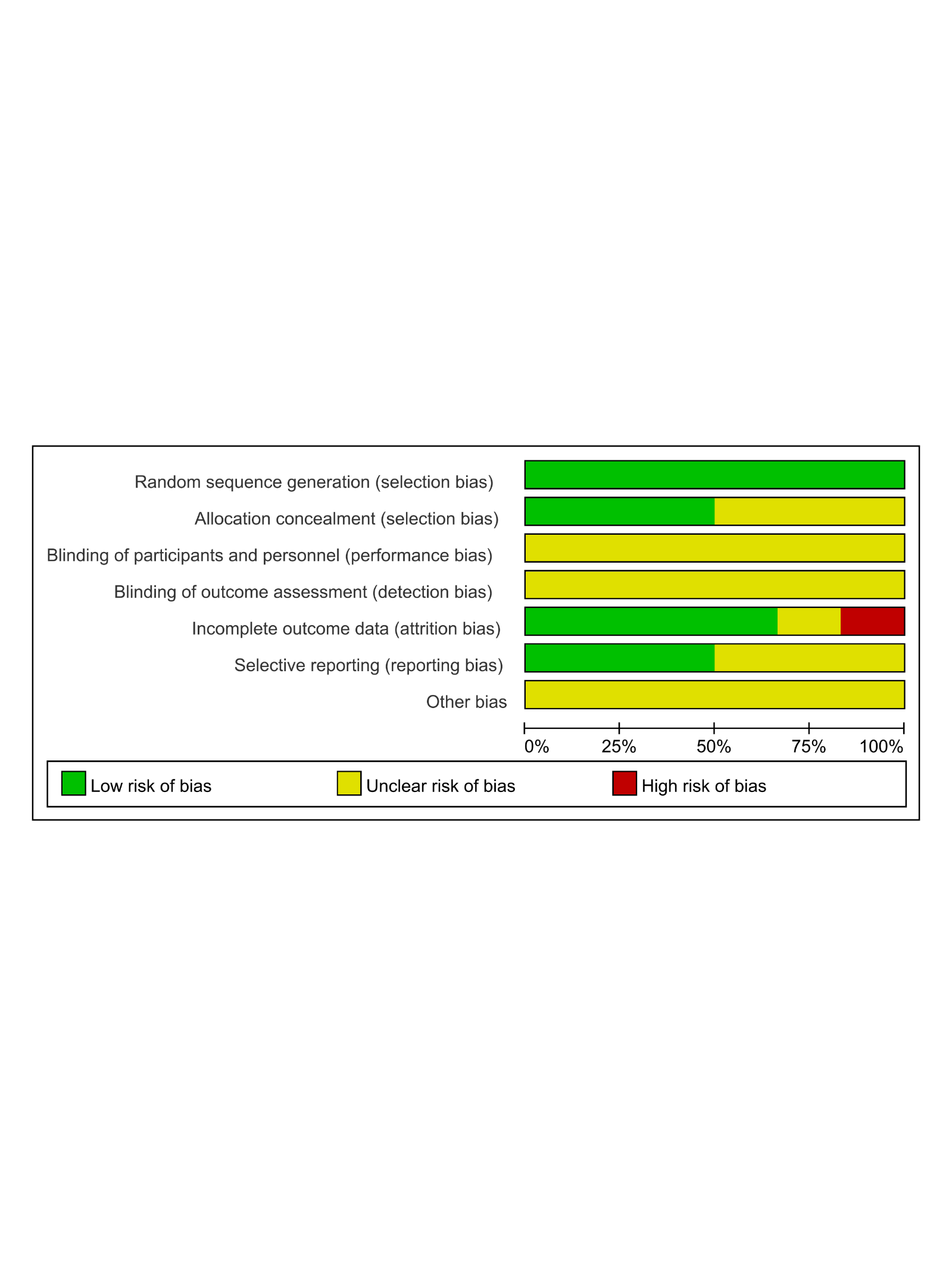
**

**Fig 3. Risk of bias summary for randomized controlled trials.**

**
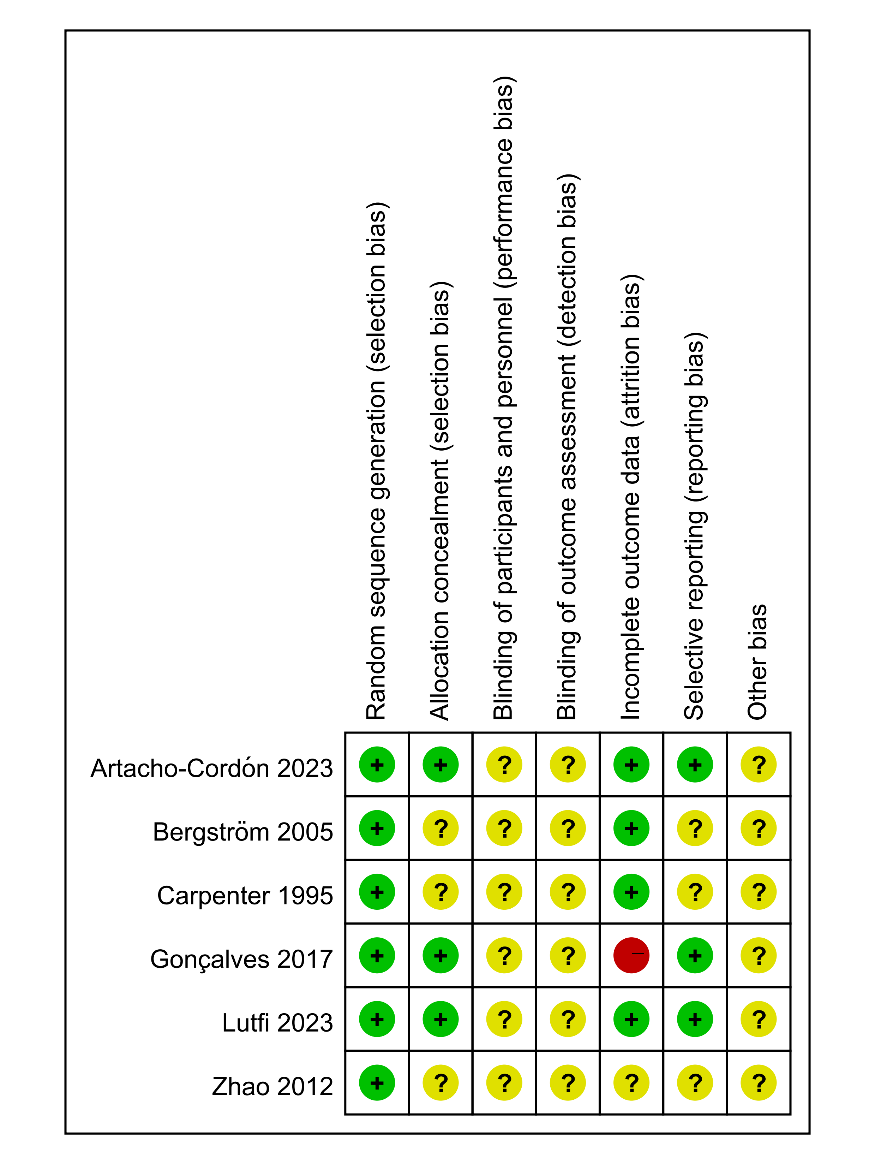
**

**Fig 4.** **Forest plot of the analysis of the pain aspect of quality of life.**

**
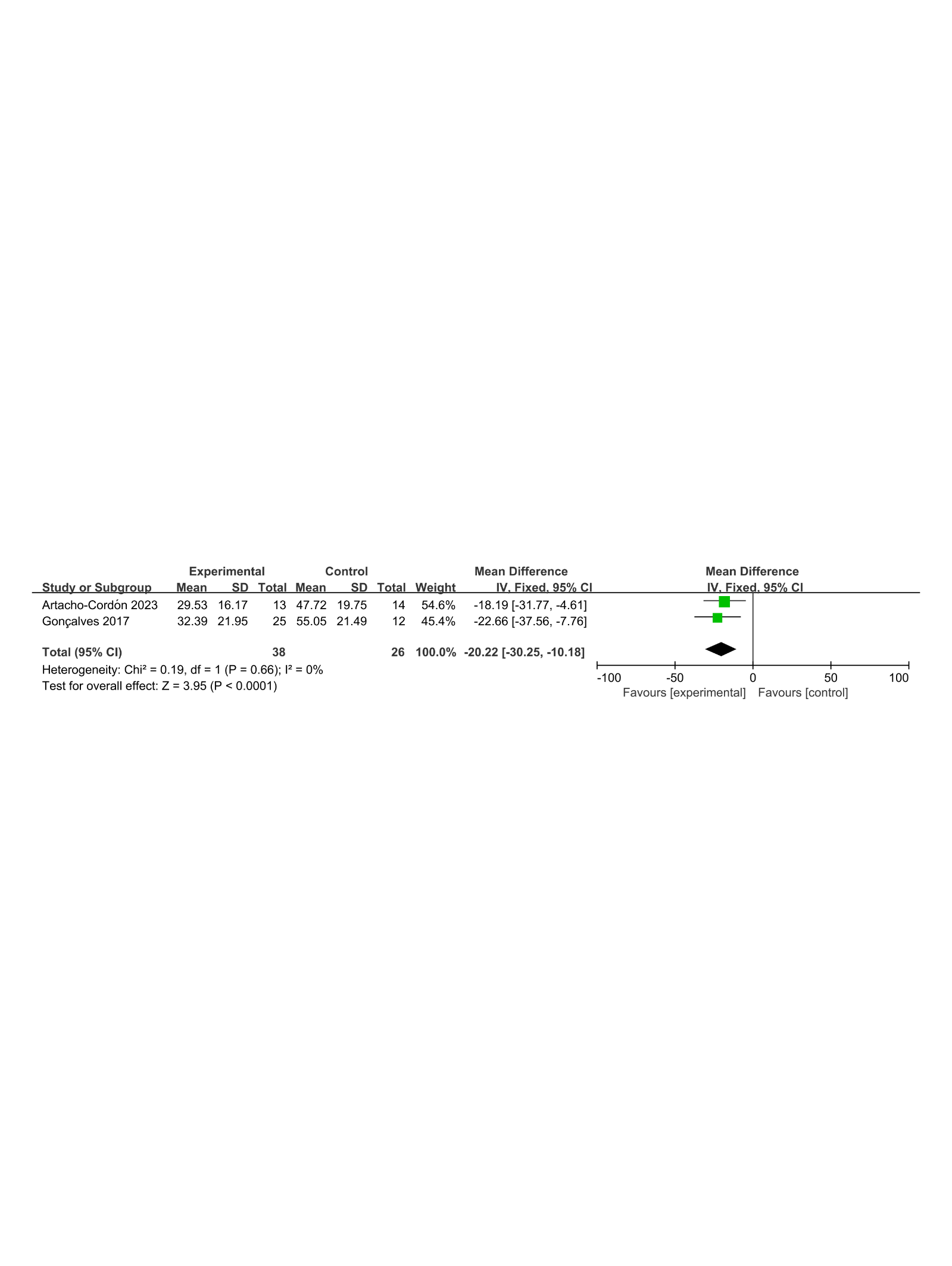
**

**Fig 5.** **Forest plot of the analysis of the control and powerlessness aspect of quality of life.**

**
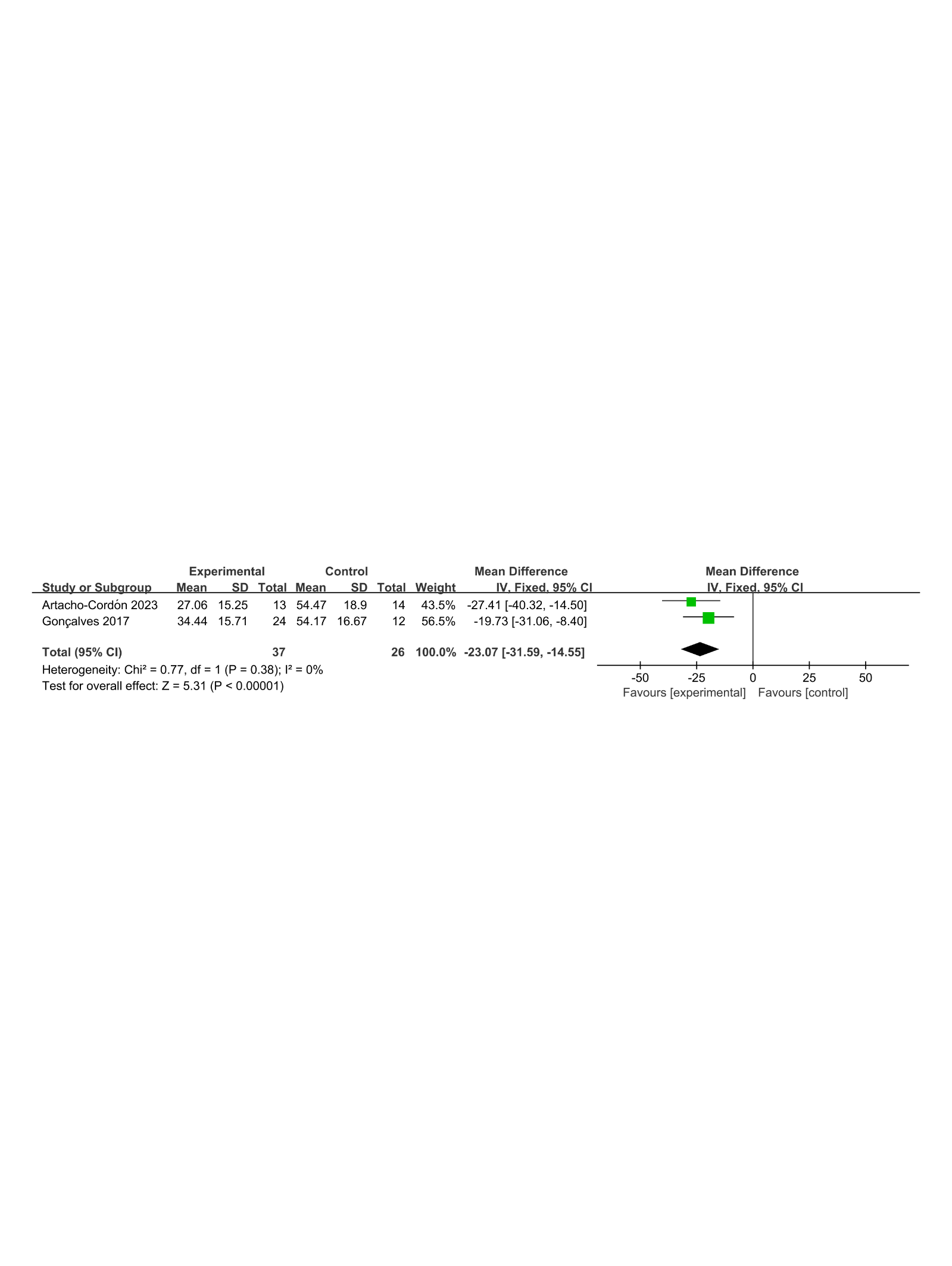
**

**Fig 6. Forest plot of the analysis of the emotional well-being aspect of quality of life.**

**
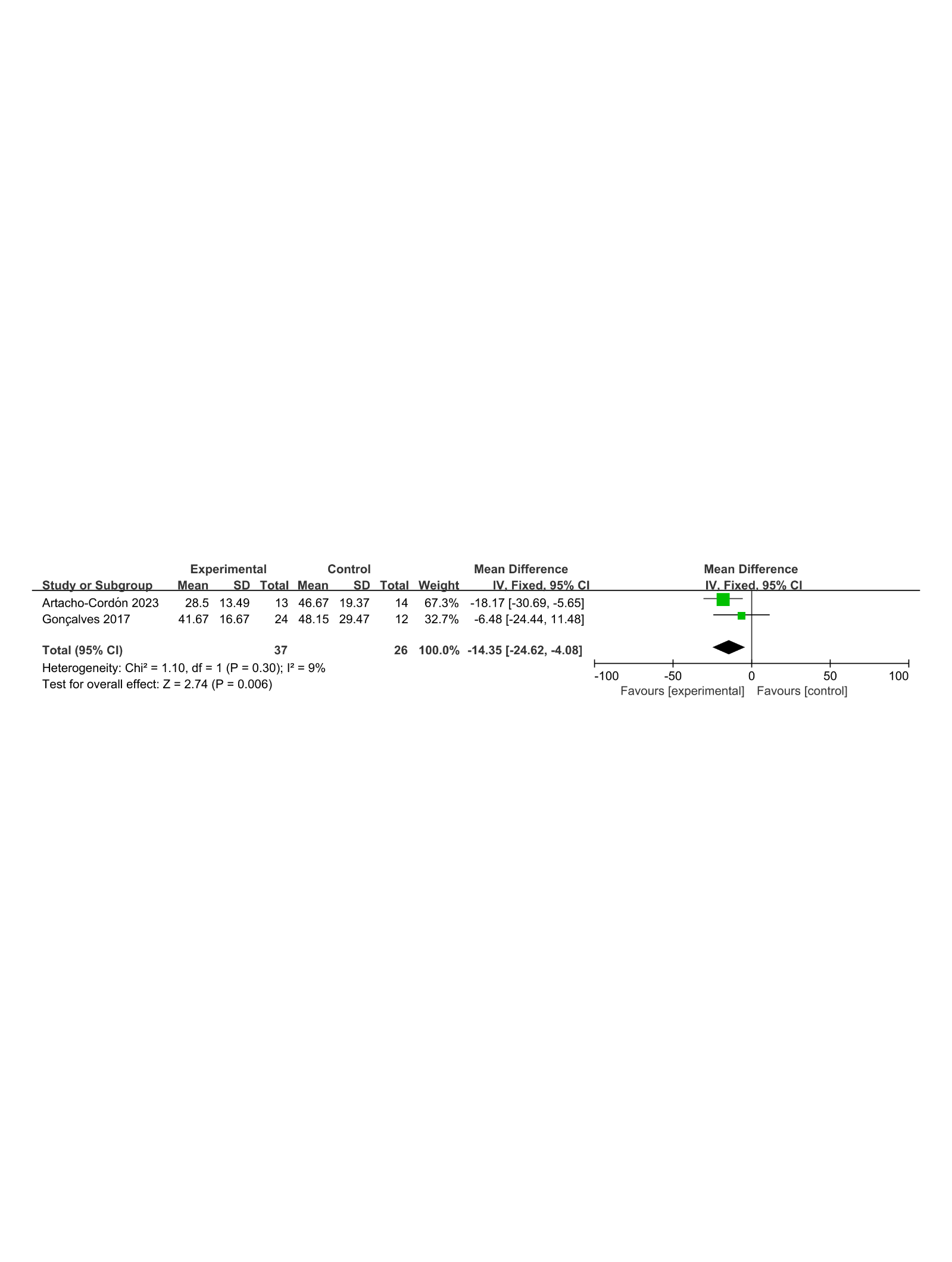
**

**Fig 7. Forest plot of the analysis of the social support of quality of life.**

**
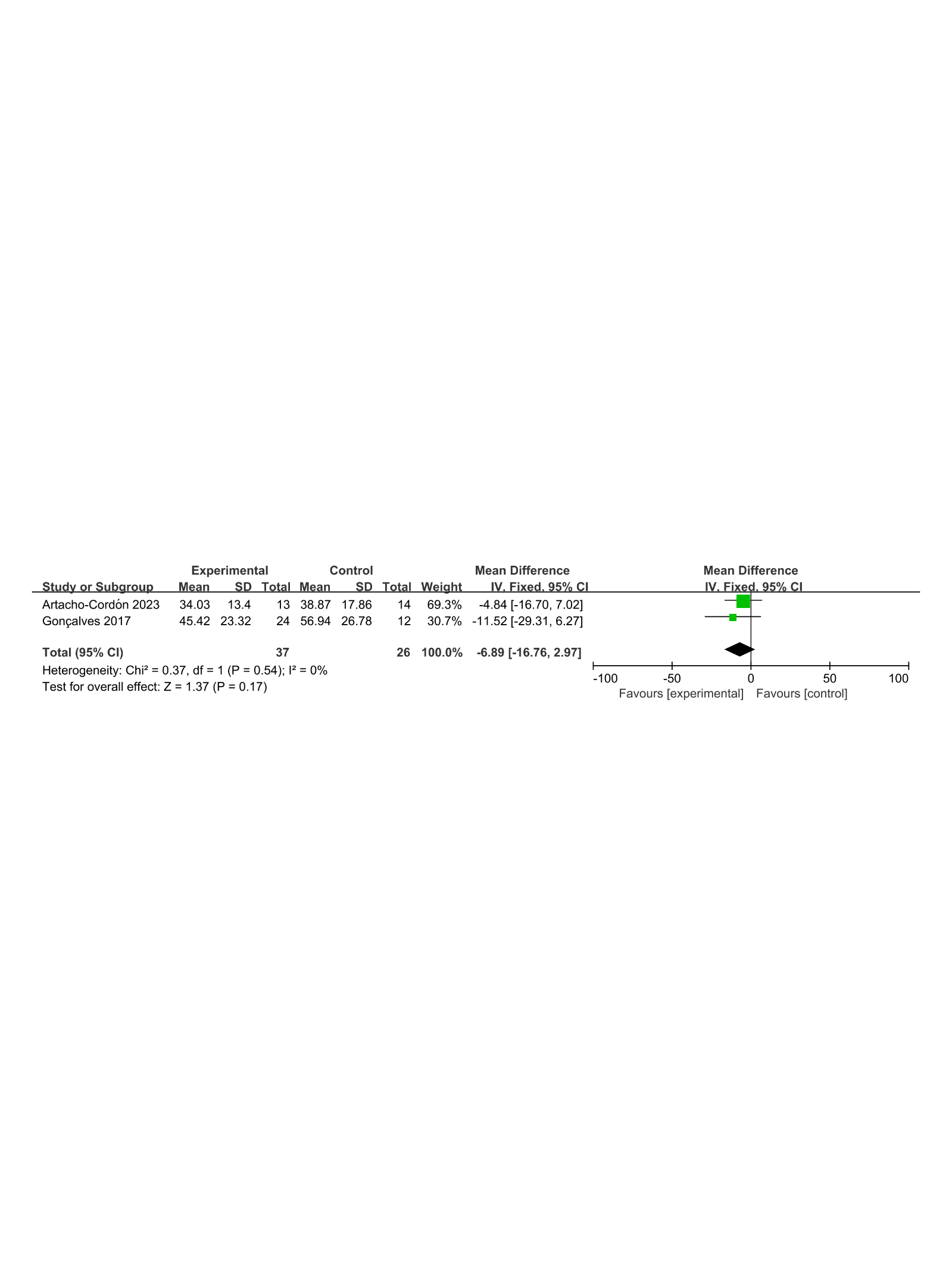
**

**Fig 8. Forest plot of the analysis of the self-image aspects of quality of life.**

**
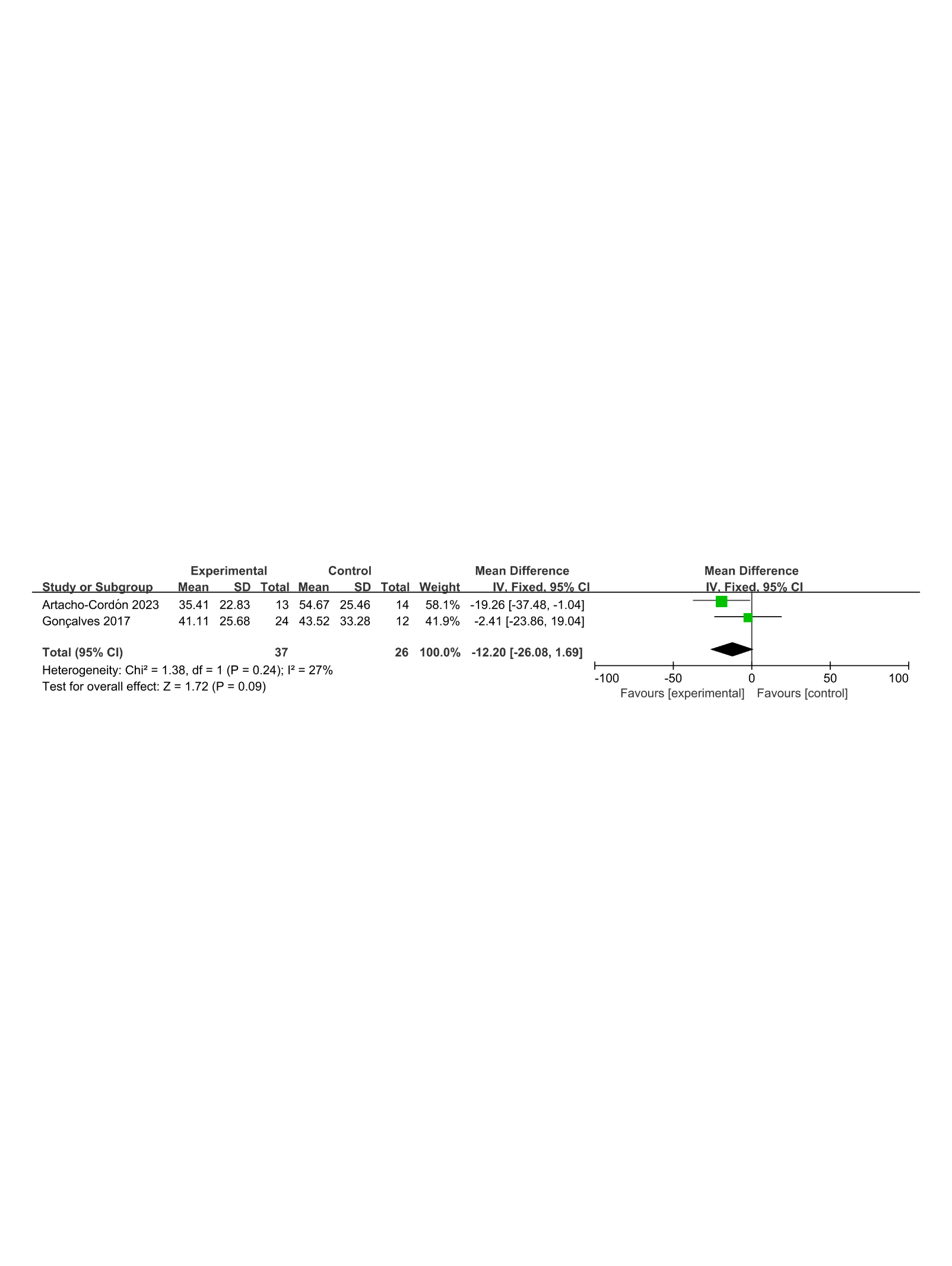
**

**References**

1. Lang AL, Bruhn RL, Fehling M, Heidenreich A, Reisdorf J, Khanyaree I, et al. Feasibility Study on Menstrual Cycles With Fitbit Device (FEMFIT): Prospective Observational Cohort Study. JMIR mHealth and uHealth. 2024;12. doi: 10.2196/50135. PubMed PMID: WOS:001186187900001.

2. Kopelman ZA, Tian C, Tumas J, Phippen NT, Tarney CM, Hope ER, et al. Disease progression, survival, and molecular disparities in Black and White patients with endometrioid endometrial carcinoma in real-world registries and GOG/NRG oncology randomized phase III clinical trials. GYNECOLOGIC ONCOLOGY. 2024;183:103-14. doi: <https://dx.doi.org/10.1016/j.ygyno.2024.03.026>.

3. Klotz SGR, Kolbe C, Ruess M, Brünahl CA. The role of psychosocial factors in the interprofessional management of women with chronic pelvic pain: A systematic review. ACTA OBSTETRICIA ET GYNECOLOGICA SCANDINAVICA. 2024;103(2):199-209. doi: 10.1111/aogs.14708. PubMed PMID: WOS:001101596600001.

4. Zhang X, Huangfu Z, Wang S. Review of mendelian randomization studies on age at natural menopause. Frontiers in Endocrinology. 2023;14. doi: 10.3389/fendo.2023.1234324.

5. Xie Q, Hu B. Effects of gut microbiota on prostatic cancer: a two-sample Mendelian randomization study. Frontiers in Microbiology. 2023;14. doi: 10.3389/fmicb.2023.1250369.

6. Wang J, Zhao H, Zhu J, Jiang M. Causal effects of physical activity on the risk of overall ovarian cancer: A Mendelian randomization study. Digit Health. 2023;9:20552076231162988. Epub 20230315. doi: 10.1177/20552076231162988. PubMed PMID: 36937699; PubMed Central PMCID: PMCPMC10017925.

7. Tempfer C. Early detection, risk factors, and prevention of endometrial cancer. Onkologie. 2023;29(5):396-405. doi: 10.1007/s00761-022-01271-z.

8. Shapiro GI, Basu B, El-Khoueiry AB, Postel-Vinay S, Im SA, Rha SY, et al. 821TiP Phase I study of ceralasertib (cerala) in combination with AZD5305 in patients (pts) with advanced/metastatic ovarian cancer (OC) previously treated with PARP inhibitors (PARPis). ANNALS OF ONCOLOGY. 2023;34:S542. doi: 10.1016/j.annonc.2023.09.1996.

9. Ray-Coquard IL, Pignata S, Lee JY, Coleman RL, Brown J, Kim JW, et al. 747MO First results from the ENGOT-GYN2/GOG-3051/BOUQUET phase II biomarker-directed platform study: Cobimetinib (cobi) or atezolizumab (atezo) + bevacizumab (bev) for persistent/recurrent rare epithelial ovarian cancer (eOC). ANNALS OF ONCOLOGY. 2023;34:S511-S2. doi: 10.1016/j.annonc.2023.09.1926.

10. Nct. Mindful Movement for Pelvic Pain. <https://clinicaltrialsgov/show/NCT05899088>. 2023. PubMed PMID: CN-02573118.

11. Kobel M, Kang E-Y, Weir A, Rambau PF, Lee C-H, Nelson GS, et al. p53 and ovarian carcinoma survival: an Ovarian Tumor Tissue Analysis consortium study. The journal of pathology Clinical research. 2023;9(3):208-22. doi: <https://dx.doi.org/10.1002/cjp2.311>.

12. Kadah S, Soh SE, Morin M, Schneider M, Ang WC, McPhate L, et al. Are pelvic pain and increased pelvic floor muscle tone associated in women with persistent noncancer pelvic pain? A systematic review and meta-analysis. The journal of sexual medicine. 2023;20(9):1206-21. doi: 10.1093/jsxmed/qdad089.

13. Gonzalez Martin A, Rubio Perez MJ, Heitz F, Christensen RD, Colombo N, Van Gorp T, et al. LBA37 Atezolizumab (atezo) combined with platinum-based chemotherapy (CT) and maintenance niraparib for recurrent ovarian cancer (rOC) with a platinum-free interval (TFIp) >6 months: Primary analysis of the double-blind placebo (pbo)-controlled ENGOT-Ov41/GEICO 69-O/ANITA phase III trial. ANNALS OF ONCOLOGY. 2023;34:S1278-S9. doi: 10.1016/j.annonc.2023.10.031.

14. Chu Y, Wang L, Xie J, Yang S, Liu S, Hu D, et al. Impact of growth hormone on IVF/ICSI outcomes and endometrial receptivity of patients undergoing GnRH antagonist protocol with fresh embryo transfer: a pilot study. Frontiers in Endocrinology. 2023;14. doi: 10.3389/fendo.2023.1225121. PubMed PMID: CN-02622975.

15. Chen JW, Yang KB, Qiu YY, Lai WJ, Qi SF, Wang GY, et al. Genetic associations of leisure sedentary behaviors and the risk of 15 site-specific cancers: A Mendelian randomization study. Cancer Medicine. 2023;12(12):13623-36. doi: 10.1002/cam4.5974. PubMed PMID: WOS:000981376600001.

16. Blanc-Durand F, Kramer C, Rouleau E, Vasseur D, Bosse T, de Boer S, et al. Molecular profiling of p53 mutant endometrial cancer reveals distinct subgroups with opportunities for personalized therapeutic approaches. ANNALS OF ONCOLOGY. 2023;34:S529-S30. doi: 10.1016/j.annonc.2023.09.1966.

17. Slomovitz BM, Filiaci VL, Walker JL, Taub MC, Finkelstein KA, Moroney JW, et al. A randomized phase II trial of everolimus and letrozole or hormonal therapy in women with advanced, persistent or recurrent endometrial carcinoma: A GOG Foundation study. GYNECOLOGIC ONCOLOGY. 2022;164(3):481-91. doi: <https://dx.doi.org/10.1016/j.ygyno.2021.12.031>.

18. Guy M, Foucher C, Juhel C, Rigaudier F, Mayeux G, Levesque A. Transcutaneous electrical neurostimulation relieves primary dysmenorrhea: A randomized, double-blind clinical study versus placebo. Prog Urol. 2022;32(7):487-97. Epub 20220303. doi: 10.1016/j.purol.2022.01.005. PubMed PMID: 35249825.

19. Gonzalez Martin AJ, Pothuri B, Vergote IB, Graybill W, Mirza MR, McCormick C, et al. PRIMA/ENGOT-OV26/GOG-3012 study: Updated long-term PFS and safety. ANNALS OF ONCOLOGY. 2022;33:S789. doi: 10.1016/j.annonc.2022.07.658.

20. duPont NC, Enserro D, Brady MF, Moxley K, Walker JL, Cosgrove C, et al. Prognostic significance of ethnicity and age in advanced stage epithelial ovarian cancer: An NRG oncology/gynecologic oncology group study. GYNECOLOGIC ONCOLOGY. 2022;164(2):398-405. doi: <https://dx.doi.org/10.1016/j.ygyno.2021.11.013>.

21. Devroe J, Peeraer K, D'Hooghe TM, Boivin J, Laenen A, Vriens J, et al. Great expectations of IVF patients: the role of gender, dispositional optimism and shared IVF prognoses. Human reproduction (Oxford, England). 2022;37(5):997-1006. doi: <https://dx.doi.org/10.1093/humrep/deac038>.

22. Colombo N, Van Gorp T, Matulonis UA, Oaknin A, Grisham RN, Fleming G, et al. Relacorilant + nab-paclitaxel in patients with recurrent, platinum-resistant ovarian cancer: Phase II subgroup analysis mirroring the patient population of an upcoming phase III study. ANNALS OF ONCOLOGY. 2022;33:S793. doi: 10.1016/j.annonc.2022.07.664.

23. Carralero-Martinez A, Perez MAM, Kauffmann S, Blanco-Ratto L, Ramirez-Garcia I. Efficacy of capacitive resistive monopolar radiofrequency in the physiotherapeutic treatment of chronic pelvic pain syndrome: A randomized controlled trial. NEUROUROLOGY AND URODYNAMICS. 2022;41(4):962-72. doi: 10.1002/nau.24903. PubMed PMID: WOS:000766391300001.

24. Actrn. pRophylactic utErosacral suspension AT Total lAparoscopiC Hysterectomy and the risk of prolapse occurrence – a randomised controlled trial (REATTACH). <https://trialsearchwhoint/Trial2aspx?TrialID=ACTRN12622000947707>. 2022. PubMed PMID: CN-02428884.

25. Singh G, Swain AC, Mallick B. Delineating characteristic sequence and structural features of precursor and mature piwi-interacting rnas of epithelial ovarian cancer. Current Bioinformatics. 2021;16(4):541-52. doi: 10.2174/1574893615999200715164755.

26. Rahimy E, Usoz M, von Eyben R, Fujimoto D, Watanabe D, Karam A, et al. Phase II trial evaluating efficacy of a Fitbit program for improving the health of endometrial cancer survivors. GYNECOLOGIC ONCOLOGY. 2021;161(1):275-81. doi: <https://dx.doi.org/10.1016/j.ygyno.2021.01.033>.

27. Heinzelmann-Schwarz VA, Kurzeder C, Schmid S, Gabriel N, Mueller A, Fehr MK, et al. ENGOT-ov54/Swiss-GO-2/MATAO including LOGOS (Low-Grade Ovarian cancer Sub-study): MAintenance Therapy with Aromatase inhibitor in epithelial Ovarian cancer-A randomized, double-blinded, placebocontrolled, multicenter phase III Trial. JOURNAL OF CLINICAL ONCOLOGY. 2021;39(15 SUPPL). doi: 10.1200/JCO.2021.39.15_suppl.TPS5598.

28. Fernandez-Montoli ME, Sabadell J, Contreras-Perez NA. Fertility-Sparing Treatment for Atypical Endometrial Hyperplasia and Endometrial Cancer: A Cochrane Systematic Review Protocol. ADVANCES IN THERAPY. 2021;38(5):2717-31. doi: 10.1007/s12325-021-01693-y.

29. Duska LR, Filiaci VL, Walker JL, Holman LL, Hill EK, Moore RG, et al. A Surgical Window Trial Evaluating Medroxyprogesterone Acetate with or without Entinostat in Patients with Endometrial Cancer and Validation of Biomarkers of Cellular Response. Clinical cancer research : an official journal of the American Association for Cancer Research. 2021;27(10):2734-41. doi: <https://dx.doi.org/10.1158/1078-0432.CCR-20-4618>.

30. Alves R, Gomes T, Baqueiro P, Meyer PF, Barros D, Schiattarella A, et al. A Standardized Evaluation Method for Assessing Patients With Genital Dyschromia. CUREUS JOURNAL OF MEDICAL SCIENCE. 2021;13(6). doi: 10.7759/cureus.15840. PubMed PMID: WOS:000664669000021.

31. Alshehre SM, Narice BF, Fenwick MA, Metwally M. The impact of endometrioma on in vitro fertilisation/intra-cytoplasmic injection IVF/ICSI reproductive outcomes: a systematic review and meta-analysis. ARCHIVES OF GYNECOLOGY AND OBSTETRICS. 2021;303(1):3-16. doi: <https://dx.doi.org/10.1007/s00404-020-05796-9>.

32. Valabrega G, Pothuri B, Oaknin A, Graybill W, Sánchez AB, McCormick C, et al. Efficacy and safety of niraparib in older patients (pts) with advanced ovarian cancer (OC): Results from the PRIMA/ENGOT-OV26/GOG-3012 trial. ANNALS OF ONCOLOGY. 2020;31:S619. doi: 10.1016/j.annonc.2020.08.958.

33. Randall LM, O'Malley DM, Monk BJ, Coleman RL, O'Cearbhaill RE, Gaillard S, et al. MOONSTONE/GOG-3032: A phase II, open-label, single-arm study to evaluate the efficacy and safety of niraparib + dostarlimab in patients with platinum-resistant ovarian cancer. ANNALS OF ONCOLOGY. 2020;31:S646-S7. doi: 10.1016/j.annonc.2020.08.1022.

34. Peng H, Wu X, Li C, Liang W, He J. Impact of genetically predicted elevated concentrations of C-reactive protein on ovarian cancer risk: A Mendelian randomization study. ANNALS OF ONCOLOGY. 2020;31:S1339. doi: 10.1016/j.annonc.2020.10.240.

35. Mavrichev S. Comparative assessment of survival rate in endometrial cancer intermediate risk. INTERNATIONAL JOURNAL OF GYNECOLOGICAL CANCER. 2020;30(SUPPL 3):A139. doi: 10.1136/ijgc-2020-IGCS.294.

36. Peters EEM, Bartosch C, McCluggage WG, Genestie C, Lax SF, Nout R, et al. Reproducibility of lymphovascular space invasion (LVSI) assessment in endometrial cancer. HISTOPATHOLOGY. 2019;75(1):128-36. doi: <https://dx.doi.org/10.1111/his.13871>.

37. Michels KA, Trabert B. Metabolic syndrome and risk of ovarian cancer in the United States: An analysis of linked seer-medicare data. CLINICAL CANCER RESEARCH. 2019;25(22). doi: 10.1158/1557-3265.OVCASYMP18-AP06.

38. Maxwell-Smith C, Hince D, Cohen PA, Bulsara MK, Boyle T, Platell C, et al. A randomized controlled trial of WATAAP to promote physical activity in colorectal and endometrial cancer survivors. PSYCHO-ONCOLOGY. 2019;28(7):1420-9. doi: <https://dx.doi.org/10.1002/pon.5090>.

39. Marcisauskas S, Ulfenborg B, Kristjansdottir B, Waldemarson S, Sundfeldt K. Univariate and classification analysis reveals potential diagnostic biomarkers for early stage ovarian cancer Type 1 and Type 2. Journal of Proteomics. 2019;196:57-68. doi: <https://dx.doi.org/10.1016/j.jprot.2019.01.017>.

40. Lerdpornkulrat T, Poondej C, Koul R, Khiawrod G, Prasertsirikul P. The Positive Effect of Intrinsic Feedback on Motivational Engagement and Self-Efficacy in Information Literacy. JOURNAL OF PSYCHOEDUCATIONAL ASSESSMENT. 2019;37(4):421‐34. doi: 10.1177/0734282917747423. PubMed PMID: CN-02417277.

41. González Martín A, Pothuri B, Vergote IB, Christensen RD, Graybill W, Mirza MR, et al. Niraparib therapy in patients with newly diagnosed advanced ovarian cancer (PRIMA/ENGOT-OV26/GOG-3012 study). ANNALS OF ONCOLOGY. 2019;30:v893. doi: 10.1093/annonc/mdz394.052.

42. Caruso S, Monaco C. Dyspareunia in Women: Updates in Mechanisms and Current/Novel Therapies. CURRENT SEXUAL HEALTH REPORTS. 2019;11(1):9-20. doi: 10.1007/s11930-019-00188-w. PubMed PMID: WOS:000703023900002.

43. Zou X, Zhang Y, Zhang L, Li J, Zhu C, Cheng Q, et al. Association between MDM2 SNP309 and endometrial cancer risk A PRISMA-compliant meta-analysis. Medicine (United States). 2018;97(49). doi: 10.1097/MD.0000000000013273.

44. Nct. Short Course Vaginal Cuff Brachytherapy in Treating Participants With Stage I-II Endometrial Cancer. <https://clinicaltrialsgov/ct2/show/NCT03422198>. 2018. PubMed PMID: CN-01522646.

45. Nct. The Effects of Exercise on Distress, Quality of Life, and Biomarkers in Ovarian Cancer Survivors. <https://clinicaltrialsgov/show/NCT03641287>. 2018. PubMed PMID: CN-01662486.

46. Jarrell JF, Vilos GA, Allaire C, Burgess S, Fortin C, Gerwin R, et al. No 164 - Directive clinique de consensus pour la prise en charge de la douleur pelvienne chronique. Journal of Obstetrics and Gynaecology Canada. 2018;40(11):e788-e836. doi: 10.1016/j.jogc.2018.08.017.

47. Gaubeca-Gilarranz A, Fernández-de-las-Peñas C, Medina-Torres JR, Seoane-Ruiz JM, Company-Palonés A, Cleland JA, et al. Effectiveness of dry needling of rectus abdominis trigger points for the treatment of primary dysmenorrhoea: a randomised parallel-group trial. Acupuncture in Medicine. 2018;36(5):302-10. doi: 10.1136/acupmed-2017-011566. PubMed PMID: WOS:000448518900004.

48. Bishop EA, Java JJ, Moore KN, Spirtos NM, Pearl ML, Zivanovic O, et al. Surgical outcomes among elderly women with endometrial cancer treated by laparoscopic hysterectomy: a NRG/Gynecologic Oncology Group study. AMERICAN JOURNAL OF OBSTETRICS AND GYNECOLOGY. 2018;218(1):109.e1-.e11. doi: <https://dx.doi.org/10.1016/j.ajog.2017.09.026>.

49. Tempfer C. Endometrial cancer: Epidemiology and etiology. Onkologe. 2017;23(1):7-14. doi: 10.1007/s00761-016-0095-4.

50. Phelan CM, Kuchenbaecker KB, Tyrer JP, Kar SP, Lawrenson K, Winham SJ, et al. Identification of 12 new susceptibility loci for different histotypes of epithelial ovarian cancer. NATURE GENETICS. 2017;49(5):680-91. doi: <https://dx.doi.org/10.1038/ng.3826>.

51. Joly Lobbedez F, Floquet A, Kalbacher E, Heutte N, Berton-Rigaud D, Tredan O, et al. Long term quality of life among epithelial ovarian cancer patients: The GINECO case/control VIVROVAIRE Study. ANNALS OF ONCOLOGY. 2017;28:v334. doi: 10.1093/annonc/mdx372.010.

52. Janda M, Gebski V, Davies LC, Forder P, Brand A, Hogg R, et al. Effect of Total Laparoscopic Hysterectomy vs Total Abdominal Hysterectomy on Disease-Free Survival Among Women With Stage I Endometrial Cancer: A Randomized Clinical Trial. JAMA. 2017;317(12):1224-33. doi: <https://dx.doi.org/10.1001/jama.2017.2068>.

53. Gharebaghi A, Amiri I, Salehi I, Shahidi S, Komaki A, Mehdizadeh M, et al. Treadmill exercise attenuates 3,4-methylenedioxymethamphetamine-induced memory impairment through a decrease apoptosis in male rat hippocampus. JOURNAL OF NEUROSCIENCE RESEARCH. 2017;95(12):2448-55. doi: <https://dx.doi.org/10.1002/jnr.24078>.

54. Burnett M, Lemyre M. No. 345-Primary Dysmenorrhea Consensus Guideline. Journal of Obstetrics and Gynaecology Canada. 2017;39(7):585-95. doi: 10.1016/j.jogc.2016.12.023.

55. Bishop EA, Java JJ, Moore KN, Walker JL. Pathologic and Treatment Outcomes Among a Geriatric Population of Endometrial Cancer Patients: An NRG Oncology/Gynecologic Oncology Group Ancillary Data Analysis of LAP2. International journal of gynecological cancer : official journal of the International Gynecological Cancer Society. 2017;27(4):730-7. doi: <https://dx.doi.org/10.1097/IGC.0000000000000947>.

56. Garcia-Soto AE, Java JJ, Nieves Neira W, Pearson JM, Cohn DE, Lele SB, et al. Does time interval between surgery and intraperitoneal chemotherapy administration in advanced ovarian cancer carry a prognostic impact? An NRG Oncology/Gynecologic Oncology Group study ancillary study. GYNECOLOGIC ONCOLOGY. 2016;143(3):484-9. doi: <https://dx.doi.org/10.1016/j.ygyno.2016.10.003>.

57. Fader AN, Java J, Tenney M, Ricci S, Gunderson CC, Temkin SM, et al. Impact of histology and surgical approach on survival among women with early-stage, high-grade uterine cancer: An NRG Oncology/Gynecologic Oncology Group ancillary analysis. GYNECOLOGIC ONCOLOGY. 2016;143(3):460-5. doi: <https://dx.doi.org/10.1016/j.ygyno.2016.10.016>.

58. Eskander RN, Ali S, Dellinger T, Lankes HA, Randall LM, Ramirez NC, et al. Expression Patterns of the Wnt Pathway Inhibitors Dickkopf3 and Secreted Frizzled-Related Proteins 1 and 4 in Endometrial Endometrioid Adenocarcinoma: An NRG Oncology/Gynecologic Oncology Group Study. International journal of gynecological cancer : official journal of the International Gynecological Cancer Society. 2016;26(1):125-32. doi: <https://dx.doi.org/10.1097/IGC.0000000000000563>.

59. Koushik A, Wang M, Anderson KE, van den Brandt P, Clendenen TV, Eliassen AH, et al. Intake of vitamins A, C, and E and folate and the risk of ovarian cancer in a pooled analysis of 10 cohort studies. Cancer Causes and Control. 2015;26(9):1315-27. doi: 10.1007/s10552-015-0626-0.

60. Felix AS, Scott McMeekin D, Mutch D, Walker JL, Creasman WT, Cohn DE, et al. Associations between etiologic factors and mortality after endometrial cancer diagnosis: the NRG Oncology/Gynecologic Oncology Group 210 trial. GYNECOLOGIC ONCOLOGY. 2015;139(1):70-6. doi: <https://dx.doi.org/10.1016/j.ygyno.2015.08.022>.

61. Zaino RJ, Brady WE, Todd W, Leslie K, Fischer EG, Horowitz NS, et al. Histologic effects of medroxyprogesterone acetate on endometrioid endometrial adenocarcinoma: a Gynecologic Oncology Group study. International journal of gynecological pathology : official journal of the International Society of Gynecological Pathologists. 2014;33(6):543-53. doi: <https://dx.doi.org/10.1097/PGP.0000000000000177>.

62. Leong FC. Complementary and alternative medications for chronic pelvic pain. OBSTETRICS AND GYNECOLOGY CLINICS OF NORTH AMERICA. 2014;41(3):503-10. doi: 10.1016/j.ogc.2014.05.001.

63. Haggerty AF, Allison K, Sarwer DB, Spitzer J, Raggio G, Chu C. The use of technology-based weight loss intervention for endometrial cancer survivors with obesity. GYNECOLOGIC ONCOLOGY. 2014;133:50-1. doi: 10.1016/j.ygyno.2014.03.142.

64. Nugent E, Wild R, Moxley K, Nelson T, Bishop E, Mathews C, et al. Prospective lifestyle modification in patients with good prognosis endo- metrioid adenocarcinoma of the uterus. GYNECOLOGIC ONCOLOGY. 2013;130(1):e67-e8. doi: 10.1016/j.ygyno.2013.04.222.

65. Brinton LA, Felix AS, McMeekin DS, Creasman WT, Sherman ME, Mutch D, et al. Etiologic heterogeneity in endometrial cancer: evidence from a Gynecologic Oncology Group trial. GYNECOLOGIC ONCOLOGY. 2013;129(2):277-84. doi: <https://dx.doi.org/10.1016/j.ygyno.2013.02.023>.

66. Rice MS, Murphy MA, Tworoger SS. Tubal ligation, hysterectomy and ovarian cancer: A meta-analysis. Journal of Ovarian Research. 2012;5(1). doi: 10.1186/1757-2215-5-13.

67. Kerr SE, Holtegaard LM, Peterson LM, Medeiros F, Mariani A, Highsmith WE, et al. Targeted mutation analysis of endometrial cancer using a custom sequenom® massarray panel: A proof-of-principle study. LABORATORY INVESTIGATION. 2012;92:513A. doi: 10.1038/labinvest.2012.24.

68. Gilbert SA, Grobman WA, Landon MB, Spong CY, Rouse DJ, Leveno KJ, et al. Elective repeat cesarean delivery compared with spontaneous trial of labor after a prior cesarean delivery: a propensity score analysis. AMERICAN JOURNAL OF OBSTETRICS AND GYNECOLOGY. 2012;206(4):311.e1-9. doi: <https://dx.doi.org/10.1016/j.ajog.2012.02.002>.

69. Trew G, Pistofidis G, Pados G, Lower A, Mettler L, Wallwiener D, et al. Gynaecological endoscopic evaluation of 4% icodextrin solution: a European, multicentre, double-blind, randomized study of the efficacy and safety in the reduction of de novo adhesions after laparoscopic gynaecological surgery. Hum Reprod. 2011;26(8):2015-27. Epub 20110601. doi: 10.1093/humrep/der135. PubMed PMID: 21632697.

70. Ning M, Feng-juan H, Guo R. [Clinical observation of erhuang powder's promoting the wound healing of cervical columnar epithelium ectopy after physiotherapy]. Zhongguo Zhong Xi Yi Jie He Za Zhi. 2011;31(12):1601-3. PubMed PMID: 22384542.

71. Bjorge T, Lukanova A, Tretli S, Manjer J, Ulmer H, Stocks T, et al. Metabolic risk factors and ovarian cancer in the Metabolic Syndrome and Cancer project. INTERNATIONAL JOURNAL OF EPIDEMIOLOGY. 2011;40(6):1667-77. doi: 10.1093/ije/dyr130. PubMed PMID: WOS:000297868500028.

72. Slomovitz BM, Lu KH, Johnston T, Coleman RL, Munsell M, Broaddus RR, et al. A phase 2 study of the oral mammalian target of rapamycin inhibitor, everolimus, in patients with recurrent endometrial carcinoma. CANCER. 2010;116(23):5415-9. doi: <https://dx.doi.org/10.1002/cncr.25515>.

73. Köbel M, Kalloger SE, Baker PM, Ewanowich CA, Arseneau J, Zherebitskiy V, et al. Diagnosis of ovarian carcinoma cell type is highly reproducible: A transcanadian study. AMERICAN JOURNAL OF SURGICAL PATHOLOGY. 2010;34(7):984-93. doi: 10.1097/PAS.0b013e3181e1a3bb.

74. Bratby MJ, Walker WJ. Uterine artery embolisation for symptomatic adenomyosis--mid-term results. EUROPEAN JOURNAL OF RADIOLOGY. 2009;70(1):128-32. doi: <https://dx.doi.org/10.1016/j.ejrad.2007.12.009>.

75. Nct. Diet and Physical Activity Change or Usual Care in Improving Progression-Free Survival in Patients With Previously Treated Stage II, III, or IV Ovarian, Fallopian Tube, or Primary Peritoneal Cancer. <https://clinicaltrialsgov/show/NCT00719303>. 2008. PubMed PMID: CN-02039516.

76. Long CY, Liu CM, Wu TP, Hsu SC, Chang Y, Tsai EM. A randomized comparison of vesicourethral function after laparoscopic hysterectomy with and without vaginal cuff suspension. Journal of Minimally Invasive Gynecology. 2005;12(2):137‐43. doi: 10.1016/j.jmig.2005.01.017. PubMed PMID: CN-00511787.

77. Sáinz de la Cuesta R, Espinosa JA, Crespo E, Granizo JJ, Rivas F. Does fluid hysteroscopy increase the stage or worsen the prognosis in patients with endometrial cancer? A randomized controlled trial. Eur J Obstet Gynecol Reprod Biol. 2004;115(2):211-5. doi: 10.1016/j.ejogrb.2004.01.029. PubMed PMID: 15262358.

78. Fugh-Berman A, Kronenberg F. Complementary and alternative medicine (CAM) in reproductive-age women: a review of randomized controlled trials. Reprod Toxicol. 2003;17(2):137-52. doi: 10.1016/s0890-6238(02)00128-4. PubMed PMID: 12642146.

79. Rickert VI, Kozlowski KJ. Pelvic pain. A SAFE approach. Obstet Gynecol Clin North Am. 2000;27(1):181-93. doi: 10.1016/s0889-8545(00)80013-3. PubMed PMID: 10693189.

80. Weeks AD, Duffy SR, Walker JJ. Uterine ultrasonographic changes with gonadotropin-releasing hormone agonists. AMERICAN JOURNAL OF OBSTETRICS AND GYNECOLOGY. 1999;180(1 Pt 1):8-13. doi: <https://dx.doi.org/10.1016/s0002-9378(99)70140-7>.

81. Shen-Gunther J, Walker JL, Johnson GA, Mannel RS. Hepatic venoocclusive disease as a complication of whole abdominopelvic irradiation and treatment with the transjuglar intrahepatic portosystemic shunt: case report and literature review. GYNECOLOGIC ONCOLOGY. 1996;61(2):282-6. doi: <https://dx.doi.org/10.1006/gyno.1996.0140>.

82. Barlow P, Englert Y, Puissant F, Lejeune B, Delvigne A, Van Rysselberge M, et al. Fertilization failure in IVF: Why and what next? HUMAN REPRODUCTION. 1990;5(4):451-6. PubMed Central PMCID: PMCHoechst(Germany)

Serono

Organon.

83. Cramer SF, Roth LM, Ulbright TM. Evaluation of the reproducibility of the World Health Organization classification of common ovarian cancers: With emphasis on methodology. Archives of Pathology and Laboratory Medicine. 1987;111(9):819-29.

84. Powell TC, Meyer I, Redden DT, Maier J, Nguyen C, Richter HE. Pain Catastrophizing and Impact on Pelvic Floor Surgery Experience. Obstetrical and Gynecological Survey. 2024;79(3):157-9. doi: 10.1097/OGX.0000000000001258.

85. w7h2p RBR. Electrical Stimulation in women with Primary Dysmenorrhea. <https://trialsearchwhoint/Trial2aspx?TrialID=RBR-56w7h2p>. 2023. PubMed PMID: CN-02570654.

86. Ruvolo G, Camera I, Ermini B, Sapienza F, Manno M, Brucculeri AM, et al. Human sperm selection using cumulus oophorus complexes compared with conventional sperm preparation method on sperm quality and ICSI outcomes: A pilot study. HUMAN REPRODUCTION. 2023;38:i183-i4. doi: 10.1093/humrep/dead093.364.

87. Lorusso D, Xiang Y, Hasegawa K, Scambia G, Leiva Galves MH, Ramos Elias P, et al. LBA38 Pembrolizumab plus chemoradiotherapy for high-risk locally advanced cervical cancer: A randomized, double-blind, phase III ENGOT-cx11/GOG-3047/KEYNOTE-A18 study. ANNALS OF ONCOLOGY. 2023;34:S1279-S80. doi: 10.1016/j.annonc.2023.10.032.

88. Dason ES, Maxim M, Sanders A, Papillon-Smith J, Ng D, Chan C, et al. Guideline No. 437: Diagnosis and Management of Adenomyosis. Journal of Obstetrics and Gynaecology Canada. 2023;45(6):417-29.e1. doi: 10.1016/j.jogc.2023.04.008.

89. Ctri. To see the effect of Rajonivrittikar Yoga in middle aged women with rajovriddhi (Heavy Bleeding during menses) in stopping menses: study on Cell line and Clinical Trial. <https://trialsearchwhoint/Trial2aspx?TrialID=CTRI/2023/08/056124>. 2023. PubMed PMID: CN-02591436.

90. Barbagallo F, Cannarella R, Garofalo V, Marino M, La Vignera S, Condorelli RA, et al. The Role of Irisin throughout Women's Life Span. Biomedicines. 2023;11(12). doi: 10.3390/biomedicines11123260. PubMed PMID: WOS:001130675100001.

91. Robert M, Graves LE, Allen VM, Dama S, Gabrys RL, Tanguay RL, et al. Guideline No. 425a: Cannabis Use Throughout Women's Lifespans - Part 1: Fertility, Contraception, Menopause, and Pelvic Pain. Journal of obstetrics and gynaecology Canada : JOGC = Journal d'obstetrique et gynecologie du Canada : JOGC. 2022;44(4):407-19.e4. doi: <https://dx.doi.org/10.1016/j.jogc.2022.01.012>.

92. Raperport C, Qureshi D, Desai J, Bhide P. Definitions and diagnostic criteria for unexplained infertility (UI) - a systematic review. HUMAN REPRODUCTION. 2022;37:i132. doi: 10.1093/humrep/deac106.P-732.

93. Nct. Effects of Connective Tissue Manipulation Versus Stretching Exercises in Primary Dysmenorrhea. <https://clinicaltrialsgov/show/NCT05357001>. 2022. PubMed PMID: CN-02395595.

94. Nct. Evaluating Ovarian Reserve After Conventional Laparoscopy Versus Robotic Surgery for Bilateral Endometrioma. <https://clinicaltrialsgov/show/NCT05357924>. 2022. PubMed PMID: CN-02404859.

95. Najmabadi S, Schliep KC, Simonsen SE, Porucznik CA, Egger MJ, Stanford JB. Characteristics of menstrual cycles with or without intercourse in women with no known subfertility. Hum Reprod Open. 2022;2022(4):hoac039. Epub 20220927. doi: 10.1093/hropen/hoac039. PubMed PMID: 36186844; PubMed Central PMCID: PMCPMC9519089.

96. Michelini S, Boccardo F, Cestari M. ESL CONSENSUS DOCUMENT ON LIPEDEMA: PROPOSAL FOR DISCUSSION. LYMPHOLOGY. 2022;33(82):42-3.

97. Hurwitz LM, Townsend MK, Jordan SJ, Patel AV, Teras LR, Lacey JV, Jr., et al. Modification of the Association Between Frequent Aspirin Use and Ovarian Cancer Risk: A Meta-Analysis Using Individual-Level Data From Two Ovarian Cancer Consortia. Journal of clinical oncology : official journal of the American Society of Clinical Oncology. 2022;40(36):4207-17. doi: <https://dx.doi.org/10.1200/JCO.21.01900>.

98. He R, Liu R, Wu H, Yu J, Jiang Z, Huang H. The Causal Evidence of Birth Weight and Female-Related Traits and Diseases: A Two-Sample Mendelian Randomization Analysis. Frontiers in Genetics. 2022;13. doi: 10.3389/fgene.2022.850892.

99. Hatzler L, Kronthaler SM, Beier PKM. Redefining innovation in sexual medicine: patients and public involvement to inform patient-centered web-based interventions for female sexual dysfunctions. Journal of Sexual Medicine. 2022;19(11):S133. doi: 10.1016/j.jsxm.2022.10.071.

100. Chokshi K, Yih C, Sackheim K. ID:16315 Successful Treatment of Chronic Pelvic Pain With Botulinum Toxin Injections. NEUROMODULATION. 2022;25(5):S15. doi: 10.1016/j.neurom.2022.02.022.

101. Alfonso FN. Experience with nutraceutical supplements in the treatment of pelvic pain in gynaecology: case reports. Drugs in Context. 2022;11. doi: 10.7573/dic.2021-10-8.

102. Wang J, Westveld AH, Welsh AH, Parker M, Loong B. Analysis of survey on menstrual disorder among teenagers using Gaussian copula model with graphical lasso prior. PLoS One. 2021;16(3 March). doi: 10.1371/journal.pone.0248340.

103. Urits I, Schwartz R, Herman J, Berger AA, Lee D, Lee C, et al. A Comprehensive Update of the Superior Hypogastric Block for the Management of Chronic Pelvic Pain. Current Pain and Headache Reports. 2021;25(3). doi: 10.1007/s11916-020-00933-0.

104. Reavey JJ, Walker C, Nicol M, Murray AA, Critchley HOD, Kershaw LE, et al. Markers of human endometrial hypoxia can be detected in vivo and ex vivo during physiological menstruation. Human reproduction (Oxford, England). 2021;36(4):941-50. doi: <https://dx.doi.org/10.1093/humrep/deaa379>.

105. Khan KN, Fujishita A, Suematsu T, Ogawa K, Koshiba A, Mori T, et al. An axonemal alteration in apical endometria of human adenomyosis. HUMAN REPRODUCTION. 2021;36(6):1574-89. doi: 10.1093/humrep/deab090.

106. Grynberg M, Labrosse J, Smires BB, Sifer C, Peigne M, Sonigo C. Could hormonal and follicular rearrangements explain timely menopause in unilaterally oophorectomized women? HUMAN REPRODUCTION. 2021;36(7):1941-7. doi: 10.1093/humrep/deab132.

107. Vara J, Muñoz E, Garcia S, Guijarro MJ, Rodriguez ML, Martin C, et al. Music therapy in rehabilitation treatment for chronic pelvic pain. INTERNATIONAL UROGYNECOLOGY JOURNAL. 2020;31(SUPPL 1):S206-S7. doi: 10.1007/s00192-020-04555-3.

108. Sastre Real M, Diaz de Teran J. OnabotulinumtoxinA Is an Effective Treatment for Chronic Migraine in Patients With Comorbid Fibromyalgia. Frontiers in Neurology. 2020;11:575130. doi: <https://dx.doi.org/10.3389/fneur.2020.575130>.

109. Orr N, Wahl K, Joannou A, Hartmann D, Valle L, Yong P. Deep Dyspareunia: Review of Pathophysiology and Proposed Future Research Priorities. Sexual Medicine Reviews. 2020;8(1):3-17. doi: <https://dx.doi.org/10.1016/j.sxmr.2018.12.007>.

110. Copp T, Cvejic E, McCaffery K, Hersch J, Doust J, Mol BW, et al. Impact of a diagnosis of polycystic ovary syndrome on diet, physical activity and contraceptive use in young women: Findings from the Australian Longitudinal Study of Women's Health. HUMAN REPRODUCTION. 2020;35(2):394-403. doi: 10.1093/humrep/dez274.

111. Chelladurai R, Pm G, Ss GD, Vaidyanathan H. Sleep - an underrated fertility booster? A questionnaire survey on the pattern of sleep among IVF patients and their reproductive outcome. HUMAN REPRODUCTION. 2020;35(SUPPL 1):i353.

112. Alshehre SM, Duffy S, Jones G, Ledger WL, Metwally M. A prospective, single-centre, single-arm, open label study of the long term use of a gonadotropin releasing hormone agonist (Triptorelin SR, 11.25 mg) in combination with Tibolone add-back therapy in the management of chronic cyclical pelvic pain. Reproductive biology and endocrinology : RB&E. 2020;18(1):28. doi: <https://dx.doi.org/10.1186/s12958-020-00586-z>.

113. Yarmolinsky J, Relton CL, Lophatananon A, Muir K, Menon U, Gentry-Maharaj A, et al. Appraising the role of previously reported risk factors in epithelial ovarian cancer risk: A Mendelian randomization analysis. PLOS MEDICINE. 2019;16(8). doi: 10.1371/journal.pmed.1002893.

114. Skelly C, Sheehan E, Niblock K, Johnston KM. 2887 Hysterectomy after Failed Endometrial Resection and Endometrial Ablation Techniques. Can We Work Out When It is Going to Fail? Journal of Minimally Invasive Gynecology. 2019;26(7):S106. doi: 10.1016/j.jmig.2019.09.097.

115. Sen N, Tanwar S, Ashok J, Kui SH, Ling HL, Akihiro S, et al. Spontaneous coronary artery dissection in Asian women with different clinical presentation, risk scenario and coronary angiographic evaluation: Multi centric analysis. CATHETERIZATION AND CARDIOVASCULAR INTERVENTIONS. 2019;93:S1. doi: 10.1002/ccd.28216.

116. Nct. The Efficacy of Two Types of Acupuncture on Autonomic Nervous Activity and Quality of Life in Women With Dysmenorrhea. <https://clinicaltrialsgov/show/NCT04178226>. 2019. PubMed PMID: CN-02010200.

117. Brandt JS, Hadaya O, Schuster M, Rosen T, Sauer MV, Ananth CV. A Bibliometric Analysis of Top-Cited Journal Articles in Obstetrics and Gynecology. JAMA Network Open. 2019. doi: 10.1001/jamanetworkopen.2019.18007.

118. Actrn. Hydrotherapy for women with persistent pelvic pain. <https://trialsearchwhoint/Trial2aspx?TrialID=ACTRN12619001611112>. 2019. PubMed PMID: CN-02064975.

119. Yong PJ, Williams C, Bodmer-Roy S, Ezeigwe C, Zhu S, Arion K, et al. Prospective Cohort of Deep Dyspareunia in an Interdisciplinary Setting. Journal of Sexual Medicine. 2018;15(12):1765-75. doi: 10.1016/j.jsxm.2018.10.005.

120. Oda K, Hamanishi J, Matsuo K, Hasegawa K. Genomics to immunotherapy of ovarian clear cell carcinoma: Unique opportunities for management. Gynecol Oncol. 2018;151(2):381-9. Epub 20180912. doi: 10.1016/j.ygyno.2018.09.001. PubMed PMID: 30217369; PubMed Central PMCID: PMCPMC7526052.

121. Nct. Effect of Low Level Laser Therapy and Pelvic Stabilisation Exercises on Postpartum Pelvic Girdle Pain. <https://clinicaltrialsgov/show/NCT03606720>. 2018. PubMed PMID: CN-01625905.

122. Nct. Laparascopic TAP and Hysterectomy Trial #2. <https://clinicaltrialsgov/show/NCT03711981>. 2018. PubMed PMID: CN-01664258.

123. Chan K, Howard E, Vancaillie T, Chow J. Epidural and sacral neuromodulation for visceral hyperalgesia-a case series. Australian and New Zealand Journal of Obstetrics and Gynaecology. 2018;58:45. doi: 10.1111/ajo.12874.

124. Petrov-Kondratov V, Chhabra A, Jones S. Pulsed Radiofrequency Ablation of Pudendal Nerve for Treatment of a Case of Refractory Pelvic Pain. Pain Physician. 2017;20(3):E451-e4. PubMed PMID: 28339446.

125. Muscogiuri G, Altieri B, de Angelis C, Palomba S, Pivonello R, Colao A, et al. Shedding new light on female fertility: The role of vitamin D. REVIEWS IN ENDOCRINE & METABOLIC DISORDERS. 2017;18(3):273-83. doi: <https://dx.doi.org/10.1007/s11154-017-9407-2>.

126. Mumford SL, Kim K, Browne RW, Sjaarda L, Connell MT, Wilcox B, et al. Plasma fatty acids and ovulation. FERTILITY AND STERILITY. 2017;108(3):e257.

127. Jayakumaran J, Patel SD, Gangrade BK, Narasimhulu DM, Pandian SR, Silva C. Robotic-assisted laparoscopy in reproductive surgery: a contemporary review. Journal of robotic surgery. 2017;11(2):97-109. doi: 10.1007/s11701-017-0682-4.

128. Dhont N. Prognostic factors influencing IUI success. HUMAN REPRODUCTION. 2017;32:i122.

129. Pontikaki A, Sifakis S, Spandidos DA. Endometriosis and breast cancer: A survey of the epidemiological studies. Oncology Letters. 2016;11(1):23-30. doi: 10.3892/ol.2015.3895.

130. Euctr PL. Trial to Assess MK-8342B Treatment Efficacy-Safety in Dysmenorrhea. <https://trialsearchwhoint/Trial2aspx?TrialID=EUCTR2015-004326-34-PL>. 2016. PubMed PMID: CN-01801006.

131. Bharucha AE, Lee TH. Anorectal and Pelvic Pain. Mayo Clin Proc. 2016;91(10):1471-86. doi: 10.1016/j.mayocp.2016.08.011. PubMed PMID: 27712641; PubMed Central PMCID: PMCPMC5123821.

132. Drks. Laparoscopically assisted vaginal hysterectomy (LAVH) versus vaginal hysterectomy (VH) - a prospective, randomized, double-blind study in patients with an indication for vaginal hysterectomy. <https://trialsearchwhoint/Trial2aspx?TrialID=DRKS00009356>. 2015. PubMed PMID: CN-01866152.

133. Trabert B, Pinto L, Hartge P, Kemp T, Black A, Sherman ME, et al. Pre-diagnostic serum levels of inflammation markers and risk of ovarian cancer in the Prostate, Lung, Colorectal and Ovarian Cancer (PLCO) Screening Trial. GYNECOLOGIC ONCOLOGY. 2014;135(2):297-304. doi: 10.1016/j.ygyno.2014.08.025.

134. Padilla PG, Caraballo MC, Rivera I, Appleyard C, Flores I. Effects of psychological stress in histone acetylation. FASEB JOURNAL. 2014;28(1).

135. Cheong YC, Smotra G, Williams ACDC. Non-surgical interventions for the management of chronic pelvic pain. Cochrane Database of Systematic Reviews. 2014;2014(3). doi: 10.1002/14651858.CD008797.pub2.

136. Pacis MM, Goldman MB, Fung JL, Reindollar RH. Is there anassociation between vitamin dintakeand time to conception? Data from the fast trial. FERTILITY AND STERILITY. 2013;100(3):S408. doi: 10.1016/j.fertnstert.2013.07.646.

137. Khan KN, Kitajima M, Inoue T, Tateishi S, Fujishita A, Nakashima M, et al. Additive effects of inflammation and stress reaction on Toll-like receptor 4-mediated growth of endometriotic stromal cells. Hum Reprod. 2013;28(10):2794-803. Epub 20130709. doi: 10.1093/humrep/det280. PubMed PMID: 23842561.

138. Khan KN, Kitajima M, Fujishita A, Hiraki K, Matsumoto A, Nakashima M, et al. Pelvic pain in women with ovarian endometrioma is mostly associated with coexisting peritoneal lesions. Hum Reprod. 2013;28(1):109-18. Epub 20121028. doi: 10.1093/humrep/des364. PubMed PMID: 23108348.

139. Hariharan C, Sinha A, Chaudhary RK. A review of the current status of mind body medicine in obstetric and gynecology practice. Journal of South Asian Federation of Obstetrics and Gynaecology. 2013;5(3):vii-xi. doi: 10.5005/jsafog-5-3-vii.

140. Andrews J, Yunker A, Reynolds WS, Likis FE, Sathe NA, Jerome RN. AHRQ Comparative Effectiveness Reviews. Noncyclic Chronic Pelvic Pain Therapies for Women: Comparative Effectiveness. Rockville (MD): Agency for Healthcare Research and Quality (US); 2012.

141. Andrews J, Yunker A, Reynolds WS, Likis FE, Sathe NA, Jerome RN. Noncyclic Chronic Pelvic Pain Therapies for Women: Comparative Effectiveness. 2012.

142. Agarwal A, Aponte-Mellado A, Premkumar BJ, Shaman A, Gupta S. The effects of oxidative stress on female reproduction: A review. Reproductive Biology and Endocrinology. 2012;10. doi: 10.1186/1477-7827-10-49.

143. Shahali S, Kashanian M, Azari A, Salehi R. Effects of pelvic floor muscle exercises on quality of life out comes in women with stress urinary incontinence. Physiotherapy (United Kingdom). 2011;97:eS1125. doi: 10.1016/j.physio.2011.04.002.

144. Malcolm C, Shannon D. The complex nature of chronic pelvic pain. JOURNAL OF OBSTETRICS AND GYNAECOLOGY. 2011;31:23. doi: 10.3109/01443615.2011.552340.

145. Mutsaerts MA, Groen H, ter Bogt NC, Bolster JH, Land JA, Bemelmans WJ, et al. The LIFESTYLE study: costs and effects of a structured lifestyle program in overweight and obese subfertile women to reduce the need for fertility treatment and improve reproductive outcome. A randomised controlled trial. BMC Womens Health. 2010;10:22. Epub 20100625. doi: 10.1186/1472-6874-10-22. PubMed PMID: 20579357; PubMed Central PMCID: PMCPMC2907305.

146. Itsekson A, Shepshelovich D, Kanevsky A, Seidman DS. Measurement of electrical resistance of dermal-visceral zones as a diagnostic tool for gynecologic disorders. Isr Med Assoc J. 2010;12(6):334-7. PubMed PMID: 20928985.

147. Nct. Addition of Pudendal Blocks to Pelvic Floor Physical Therapy for the Treatment of Pelvic Floor Tension Myalgia. <https://clinicaltrialsgov/show/NCT00928564>. 2009. PubMed PMID: CN-01524556.

148. Kapural L, Narouze SN, Janicki TI, Mekhail N. Spinal cord stimulation is an effective treatment for the chronic intractable visceral pelvic pain. Pain Med. 2006;7(5):440-3. doi: 10.1111/j.1526-4637.2006.00165.x. PubMed PMID: 17014604.

149. Johnson NP, Fisher RA, Braunholtz DA, Gillett WR, Lilford RJ. Survey of Australasian clinicians' prior beliefs concerning lipiodol flushing as a treatment for infertility: a Bayesian study. Aust N Z J Obstet Gynaecol. 2006;46(4):298-304. doi: 10.1111/j.1479-828X.2006.00596.x. PubMed PMID: 16866790.

150. Johnson N. Management of dysmenorrhoea. Reviews in Gynaecological and Perinatal Practice. 2006;6(1-2):57-62. doi: 10.1016/j.rigp.2005.09.008.

151. Cheong Y, William Stones R. Chronic pelvic pain: aetiology and therapy. Best practice & research Clinical obstetrics & gynaecology. 2006;20(5):695-711. doi: <https://dx.doi.org/10.1016/j.bpobgyn.2006.04.004>.

152. Ruiz Anguas J, Anta Jaen E, Duran Monterrosas L, Kably Ambe A. [Analysis of results of intrauterine insemination performed once or twice per stimulated cycle]. Analisis de los resultados de la inseminacion artificial realizada en una o dos ocasiones por ciclo estimulado. 2005;73(2):76-82.

153. Johnson NP. A review of the use of lipiodol flushing for unexplained infertility. Treat Endocrinol. 2005;4(4):233-43. doi: 10.2165/00024677-200504040-00004. PubMed PMID: 16053340.

154. Jarrell JF, Vilos GA, Allaire C, Burgess S, Fortin C, Gerwin R, et al. Consensus Guidelines for the Management of Chronic Pelvic Pain. Journal of Obstetrics and Gynaecology Canada. 2005;27(9):869-87. doi: 10.1016/S1701-2163(16)30993-8.

155. Wurn BF, Wurn LJ, King CR, Heuer MA, Roscow AS, Scharf ES, et al. Treating female infertility and improving IVF pregnancy rates with a manual physical therapy technique. MedGenMed : Medscape general medicine. 2004;6(2):51.

156. Mannix LK, Calhoun AH. Menstrual migraine. Current Treatment Options in Neurology. 2004;6(6):489-98. doi: 10.1007/s11940-004-0006-7.

157. Dey M, Constantine GD. Wyeth: The leader in women's health - Yesterday, today, and tomorrow. Sexuality, Reproduction and Menopause. 2004;2(3):181-4. doi: 10.1016/j.sram.2004.07.012. PubMed Central PMCID: PMCWyeth.

158. Nagata C, Takatsuka N, Kawakami N, Shimizu H. Soy product intake and premenopausal hysterectomy in a follow-up study of Japanese women. Eur J Clin Nutr. 2001;55(9):773-7. doi: 10.1038/sj.ejcn.1601223. PubMed PMID: 11528492.

159. Tudor-Locke C, McColl RS. Factors related to variation in premenopausal bone mineral status: a health promotion approach. OSTEOPOROSIS INTERNATIONAL. 2000;11(1):1‐24. doi: 10.1007/s001980050001. PubMed PMID: CN-01740451.

160. Karlstrom PO, Bakos O, Palmstierna M, Bergh T, Lundkvist O. Direct intraperitoneal insemination - Clinical results and comparison between two methods of sperm preparation. FERTILITY AND STERILITY. 1991;56(5):939-45. doi: 10.1016/s0015-0282(16)54669-x. PubMed Central PMCID: PMCBiotest Pharma(Switzerland).

161. Brent K, Hadden WE, Weston-Webb M, Johnson NP. After the FLUSH trial: a prospective observational study of lipiodol flushing as an innovative treatment for unexplained and endometriosis-related infertility. Aust N Z J Obstet Gynaecol. 2006;46(4):293-7. doi: 10.1111/j.1479-828X.2006.00595.x. PubMed PMID: 16866789.

162. Johnson NP, Farquhar CM, Hadden WE, Suckling J, Yu Y, Sadler L. The FLUSH trial--flushing with lipiodol for unexplained (and endometriosis-related) subfertility by hysterosalpingography: a randomized trial. Hum Reprod. 2004;19(9):2043-51. Epub 20040722. doi: 10.1093/humrep/deh418. PubMed PMID: 15271870.

163. Euctr FI. Trial 1 to Assess MK-8342B Treatment Efficacy-Safety in Dysmenorrhea plus Extension. <https://trialsearchwhoint/Trial2aspx?TrialID=EUCTR2015-004325-14-FI>. 2016. PubMed PMID: CN-01876744.

164. Casalechi M, Vieira-Lopes M, Quessada MP, Arão TC, Reis FM. Endometriosis and related pelvic pain: Association with stress, anxiety and depressive symptoms. Minerva Obstetrics and Gynecology. 2021;73(3):283-9. doi: 10.23736/S2724-606X.21.04704-3.

165. Young K, Fisher J, Kirkman M. Endometriosis and fertility: women's accounts of healthcare. Human reproduction (Oxford, England). 2016;31(3):554-62. doi: <https://dx.doi.org/10.1093/humrep/dev337>.

166. Mehedintu C, Antonovici M, Cirstoiu M, Bratila E, Comandasu D, Berceanu C, et al. Endometriosis-related inflammation and fertility. EUROPEAN JOURNAL OF CLINICAL INVESTIGATION. 2016;46:51. doi: 10.1111/eci.12604.

167. Hong ZJ, Chen LS, Li XP. Observation on the Application of Benefiting Kidney and Strengthening Spleen Therapy in Reducing Side Effect of Mirena. Guangming traditional chinese medicine [guang ming zhong yi]. 2016;31(19):7‐9. PubMed PMID: CN-01927463.

168. Lasak AM. Pelvic pain1. PM and R. 2013;5(9):S169-S70. doi: 10.1016/j.pmrj.2013.08.111.

169. Bedell S, Burrows L, Goldstein A. Two cases of hymenal stenosis in adult women. Journal of Sexual Medicine. 2013;10:176. doi: 10.1111/jsm.12150.

170. Chaibi A, Tuchin PJ. Chiropractic spinal manipulative treatment of migraine headache of 40-year duration using Gonstead method: a case study. Journal of chiropractic medicine. 2011;10(3):189-93. doi: <https://dx.doi.org/10.1016/j.jcm.2011.02.002>.

171. Sirohi D, Ng CHM, Bidargaddi N, Slater H, Parker MA, Hull ML, et al. High-Quality eHealth Websites for Information on Endometriosis: Systematic Search. JOURNAL OF MEDICAL INTERNET RESEARCH. 2024;26:e48243. doi: <https://dx.doi.org/10.2196/48243>.

172. Kim SE, Stamoulis C, Gallagher J, Draisin E, Laufer M, DiVasta A. 2. Depression in Adolescent and Adult Women with Endometriosis. Journal of Pediatric and Adolescent Gynecology. 2024;37(2):222. doi: 10.1016/j.jpag.2024.01.147.

173. Dang C, Chen Z, Chai Y, Liu P, Yu X, Liu Y, et al. Assessing the relationship between gut microbiota and endometriosis: a bidirectional two-sample mendelian randomization analysis. BMC women's health. 2024;24(1):123. doi: <https://dx.doi.org/10.1186/s12905-024-02945-z>.

174. Dai Y, Luo H, Zhu L, Yang W, Xiang H, Shi Q, et al. Dysmenorrhea pattern in adolescences informing adult endometriosis. BMC PUBLIC HEALTH. 2024;24(1):373. Epub 20240205. doi: 10.1186/s12889-024-17825-2. PubMed PMID: 38317119; PubMed Central PMCID: PMCPMC10840152.

175. Gersekowski K, Ibiebele TI, Doherty JA, Harris HR, Goodman MT, Terry KL, et al. Folate Intake and Ovarian Cancer Risk among Women with Endometriosis: A Case-Control Study from the Ovarian Cancer Association Consortium. Cancer epidemiology, biomarkers & prevention : a publication of the American Association for Cancer Research, cosponsored by the American Society of Preventive Oncology. 2023;32(8):1087-96. doi: <https://dx.doi.org/10.1158/1055-9965.EPI-23-0121>.

176. Yoshino O, Suzukamo Y, Yoshihara K, Takahashi N. Quality of Life in Japanese Patients with Dysmenorrhea or Endometriosis-Associated Pelvic Pain Treated with Extended Regimen Ethinylestradiol/Drospirenone in a Real-World Setting: A Prospective Observational Study. ADVANCES IN THERAPY. 2022;39(11):5087-104. doi: 10.1007/s12325-022-02301-3.

177. Sverrisdóttir U, Hansen S, Rudnicki M. Impact of diet on pain perception in women with endometriosis: A systematic review. Eur J Obstet Gynecol Reprod Biol. 2022;271:245-9. Epub 20220225. doi: 10.1016/j.ejogrb.2022.02.028. PubMed PMID: 35245715.

178. Nct. THERAPEUTIC EXERCISE AND EDUCATION IN PAIN NEUROPHYSIOLOGY TO IMPROVE PAIN INTENSITY IN WOMEN WITH ENDOMETRIOSIS. CLINICAL TRIAL. <https://clinicaltrialsgov/show/NCT05682235>. 2022. PubMed PMID: CN-02514732.

179. Habib N, Buzzaccarini G, Centini G, Moawad GN, Ceccaldi PF, Gitas G, et al. Impact of lifestyle and diet on endometriosis: a fresh look to a busy corner. Przeglad Menopauzalny. 2022;21(2):124-32. doi: 10.5114/pm.2022.116437.

180. Zhang Y, Ma NY. Environmental Risk Factors for Endometriosis: An Umbrella Review of a Meta-Analysis of 354 Observational Studies With Over 5 Million Populations. Frontiers in Medicine. 2021;8. doi: 10.3389/fmed.2021.680833.

181. Yong PJ. Clinical Conundrum: A 33-Year-Old With Pain Post-Orgasm and a History of Endometriosis. Journal of Obstetrics and Gynaecology Canada. 2020;42(5):625-8. doi: 10.1016/j.jogc.2020.02.003.

182. Tandon HK, Stratton P, Sinaii N, Shah J, Karp BI. Botulinum toxin for chronic pelvic pain in women with endometriosis: A cohort study of a pain-focused treatment. REGIONAL ANESTHESIA AND PAIN MEDICINE. 2019;44(9):886-92. doi: 10.1136/rapm-2019-100529. PubMed Central PMCID: PMCAllergan(United States).

183. Kitchen H, Haberland C, Trigg A, Aldhouse N, Willgoss T, Schmitz H, et al. A qualitative study of patient and clinician perspectives on item importance, scoring preferences, and clinically important differences for two patient-reported outcome measures: Endometriosis Symptom Diary (ESD) and Endometriosis Impact Scale (EIS). QUALITY OF LIFE RESEARCH. 2018;27:S41-S2. doi: 10.1007/s11136-018-1946-9.

184. Hodgson R, Lee HL, Wang R, Mol BWJ, Johnson N. Interventions for endometriosis related infertility: A, systematic review and network meta-analysis. HUMAN REPRODUCTION. 2018;33:i267-i8. doi: 10.1093/humrep/33.Supplement_1.1.

185. Vercellini P, Buggio L, Somigliana E, Dridi D, Marchese MA, Viganò P. 'Behind blue eyes'†: The association between eye colour and deep in filtrating endometriosis. HUMAN REPRODUCTION. 2014;29(10):2171-5. doi: 10.1093/humrep/deu169.

186. Khan KN, Fujishita A, Kitajima M, Hiraki K, Nakashima M, Masuzaki H. Intra-uterine microbial colonization and occurrence of endometritis in women with endometriosis†. Hum Reprod. 2014;29(11):2446-56. Epub 20140908. doi: 10.1093/humrep/deu222. PubMed PMID: 25205755.

187. Khan KN, Kitajima M, Yamaguchi N, Fujishita A, Nakashima M, Ishimaru T, et al. Role of prostaglandin E2 in bacterial growth in women with endometriosis. Hum Reprod. 2012;27(12):3417-24. Epub 20120920. doi: 10.1093/humrep/des331. PubMed PMID: 23001777.

188. Sagsveen M, Farmer JE, Prentice A, Breeze A. Gonadotrophin-releasing hormone analogues for endometriosis: bone mineral density. Cochrane Database Syst Rev. 2003;2003(4):Cd001297. doi: 10.1002/14651858.Cd001297. PubMed PMID: 14583930; PubMed Central PMCID: PMCPMC7027701.

189. Han M, Pan L, Wu B, Bian X. A case-control epidemiologic study of endometriosis. Chinese medical sciences journal = Chung-kuo i hsüeh k'o hsüeh tsa chih / Chinese Academy of Medical Sciences. 1994;9(2):114-8.

190. Pan LY. Menstrual status as risk factors of endometriosis: a case-control study. Zhonghua fu chan ke za zhi. 1993;28(3):147-9, 88.

191. Agarwal SK, Soliman AM, Pokrzywinski RM, Snabes MC, Coyne KS. Clinically Meaningful Reduction in Dyspareunia Is Associated With Significant Improvements in Health-Related Quality of Life Among Women With Moderate to Severe Pain Associated With Endometriosis: A Pooled Analysis of Two Phase III Trials of Elagolix. Journal of Sexual Medicine. 2020;17(12):2427-33. doi: 10.1016/j.jsxm.2020.08.002.

192. Ricci E, Viganò P, Cipriani S, Chiaffarino F, Bianchi S, Rebonato G, et al. Physical activity and endometriosis risk in women with infertility or pain: Systematic review and meta-analysis. Medicine (Baltimore). 2016;95(40):e4957. doi: 10.1097/md.0000000000004957. PubMed PMID: 27749551; PubMed Central PMCID: PMCPMC5059053.

193. Schulze-Rath R, Soriano LC, Lopez-Garcia E, Soriano-Gabarró M, Garcia Rodriguez LA. Under-recording of endometriosis in the health improvement network (THIN) primary care database: A validation study. PHARMACOEPIDEMIOLOGY AND DRUG SAFETY. 2014;23:402-3. doi: 10.1002/pds.3701.

194. Grundstrom H, Hammar Spagnoli G, Lövqvist L, Olovsson M. Healthcare Consumption and Cost Estimates concerning Swedish Women with Endometriosis. GYNECOLOGIC AND OBSTETRIC INVESTIGATION. 2020;85(3):237-44. doi: 10.1159/000507326.

195. Potru S, Jaycox M. Discovery of kinked medtronic ascenda intrathecal catheter. REGIONAL ANESTHESIA AND PAIN MEDICINE. 2015;40(5).

196. Budden A, Ravendran K, Abbott JA. Identifying the Problems of Randomized Controlled Trials for the Surgical Management of Endometriosis-associated Pelvic Pain. Journal of Minimally Invasive Gynecology. 2020;27(2):419-32. doi: 10.1016/j.jmig.2019.11.002.

197. Haberland C, Gater A, Wessiepe D, Schmitz H, Gerlinger C, Seitz C. Psychometric validation of the Endometriosis Symptom Diary (ESD) and Endometriosis Impact Scale (EIS): Findings from an interventional study. QUALITY OF LIFE RESEARCH. 2018;27:S42. doi: 10.1007/s11136-018-1946-9.

198. Nguyen K, McCormack L, Deans R, Nesbitt-Hawes E, Knapman B, Li F, et al. A Prospective Study of Bladder Function Following Endometriosis Surgery With Up to Eight years Follow-up. Journal of Minimally Invasive Gynecology. 2024;31(3):205-12.e4. doi: 10.1016/j.jmig.2023.11.020.

199. Nct. Therapeutic-educational Physiotherapy on Pain, Physical-functionality and Quality of Life in Women With Endometriosis. <https://clinicaltrialsgov/ct2/show/NCT06212349>. 2024. PubMed PMID: CN-02676652.

200. Nct. Is Repetitive Transcranial Magnetic Stimulation Effective in Reducing Endometriosis-associated Pain. <https://clinicaltrialsgov/ct2/show/NCT06333353>. 2024. PubMed PMID: CN-02682174.

201. Canturk MM, D'Ancona G, Francois MO, Roman H. Robotic Management of Recurrent Rectal Endometriosis After Previous Segmental Bowel Resection. Journal of Minimally Invasive Gynecology. 2024;31(3):176-7. doi: <https://dx.doi.org/10.1016/j.jmig.2023.11.021>.

202. van Haaps AP, Wijbers JV, Schreurs AMF, Vlek S, Tuynman J, De Bie B, et al. The effect of dietary interventions on pain and quality of life in women diagnosed with endometriosis: a prospective study with control group. Human reproduction (Oxford, England). 2023;38(12):2433-46. doi: <https://dx.doi.org/10.1093/humrep/dead214>.

203. Tajik P, Shahali S, Padmehr R. Effects of sensate focus technique and position changing on sexual function of women with deep-infiltrating endometriosis after surgery: A clinical trial study. International journal of reproductive biomedicine. 2023;21(6):509-20. doi: <https://dx.doi.org/10.18502/ijrm.v21i6.13638>.

204. Nct. Multimodal Physiotherapy Based on Tele-rehabilitation in Chronic Pelvic Pain Associated With Endometriosis. <https://clinicaltrialsgov/ct2/show/NCT06209346>. 2023. PubMed PMID: CN-02676602.

205. Mardon AK, Leake HB, Hayles C, Henry ML, Neumann PB, Moseley GL, et al. The Efficacy of Self-Management Strategies for Females with Endometriosis: a Systematic Review. Reprod Sci. 2023;30(2):390-407. Epub 20220429. doi: 10.1007/s43032-022-00952-9. PubMed PMID: 35488093; PubMed Central PMCID: PMCPMC9988721.

206. Kahlon BK, Simon-Collins M, Nylander E, Segars J, Singh B. A systematic review of vitamin D and endometriosis: role in pathophysiology, diagnosis, treatment, and prevention. F&S REVIEWS. 2023;4(1):1-14. doi: 10.1016/j.xfnr.2022.11.005. PubMed PMID: WOS:001208918800007.

207. Hansen KE, Brandsborg B, Kesmodel US, Forman A, Kold M, Pristed R, et al. Psychological interventions improve quality of life despite persistent pain in endometriosis: results of a 3-armed randomized controlled trial. QUALITY OF LIFE RESEARCH. 2023;32(6):1727-44. doi: 10.1007/s11136-023-03346-9.

208. fhfdt RBR. Effectiveness of Ozone Therapy Treatment of Endometriosis. <https://trialsearchwhoint/Trial2aspx?TrialID=RBR-10fhfdt4>. 2023. PubMed PMID: CN-02588028.

209. Abril-Coello R, Correyero-León M, Ceballos-Laita L, Jiménez-Barrio S. Benefits of physical therapy in improving quality of life and pain associated with endometriosis: A systematic review and meta-analysis. Int J Gynaecol Obstet. 2023;162(1):233-43. Epub 20230117. doi: 10.1002/ijgo.14645. PubMed PMID: 36571475.

210. Tajik M, Shahali S, Shadjoo K. The co-effect of sensate focus technique and sexual position changing on sexual function of women who use medical treatment for endometriosis. Journal of obstetrics and gynaecology : the journal of the Institute of Obstetrics and Gynaecology. 2022;42(8):3706-11. doi: <https://dx.doi.org/10.1080/01443615.2022.2158316>.

211. Richard E, Morin J, Murji A, Lemyre M, Laberge PY, Maheux-Lacroix S. Effect of Postoperative Hormonal Suppression on Fertility in Patients With Endometriosis After Conservative Surgery: A Systematic Review and Meta-analysis. OBSTETRICS AND GYNECOLOGY. 2022;139(6):1169-79. doi: 10.1097/AOG.0000000000004811.

212. Nct. Conventional Laparoscopy Versus Robotic Surgery for Pain Relief in Patients With Deep Infiltrating Endometriosis. <https://clinicaltrialsgov/show/NCT05362838>. 2022. PubMed PMID: CN-02398807.

213. Becker C, As-Sanie S, Abrao MS, Brown E, Arjona Ferreira JC, Wagman RB, et al. SPIRIT long-term extension study: two-year efficacy and safety of relugolix combination therapy in women with endometriosis-associated pain. HUMAN REPRODUCTION. 2022;37:i110. doi: 10.1093/humrep/deac106.O-305.

214. Actrn. Pelvic floor muscle exercises and mindfulness for women with endometriosis-associated pelvic pain. <https://trialsearchwhoint/Trial2aspx?TrialID=ACTRN12622001393741>. 2022. PubMed PMID: CN-02493724.

215. Actrn. EndoCannED – The effect of Medicinal Cannabis on emergency department presentations in people with endometriosis: a randomised, controlled feasibility study. <https://trialsearchwhoint/Trial2aspx?TrialID=ACTRN12622001560785>. 2022. PubMed PMID: CN-02512163.

216. Zhang J, Dong L, Zhang S, Wang L. Analysis of the clinical efficacy of leuprolide acetate in the treatment of obese patients with endometriosis and its role on the expression of MIF gene. CELLULAR AND MOLECULAR BIOLOGY. 2021;67(4):282-8. doi: 10.14715/cmb/2021.67.4.31.

217. Whitaker LHR, Doust A, Stephen J, Norrie J, Cooper K, Daniels J, et al. Laparoscopic treatment of isolated superficial peritoneal endometriosis for managing chronic pelvic pain in women: study protocol for a randomised controlled feasibility trial (ESPriT1). Pilot and Feasibility Studies. 2021;7(1). doi: 10.1186/s40814-020-00740-9.

218. Vignali M, Pisoni S, Gentilini D, Spada E, Solima E, Viganò P, et al. Hormonal therapy potentiates the effect of surgery on gene expression profile of peripheral blood mononuclear cells in patients affected by endometriosis. Minerva Endocrinology. 2021;46(1):90-8. doi: 10.23736/S2724-6507.20.03298-8.

219. Richard E, Maheux-Lacroix S. The Effect of Post-Operative Suppressive Treatment in Women with Endometriosis-Related Infertility. Journal of Minimally Invasive Gynecology. 2021;28(11):S145-S6. doi: 10.1016/j.jmig.2021.09.283.

220. Mirzaee F, Ahmadi A. Overview of the Effect of Complementary Medicine on Treating or Mitigating the Risk of Endometriosis. Rev Bras Ginecol Obstet. 2021;43(12):919-25. Epub 20211221. doi: 10.1055/s-0041-1735156. PubMed PMID: 34933385; PubMed Central PMCID: PMCPMC10183906.

221. Gibbons T, Georgiou EX, Cheong YC, Wise MR. Levonorgestrel-releasing intrauterine device (LNG-IUD) for symptomatic endometriosis following surgery. Cochrane Database of Systematic Reviews. 2021;(12). doi: 10.1002/14651858.CD005072.pub4. PubMed PMID: WOS:000737966900034.

222. Del Forno S, Arena A, Pellizzone V, Lenzi J, Raimondo D, Cocchi L, et al. Assessment of levator hiatal area using 3D/4D transperineal ultrasound in women with deep infiltrating endometriosis and superficial dyspareunia treated with pelvic floor muscle physiotherapy: randomized controlled trial. Ultrasound in obstetrics & gynecology : the official journal of the International Society of Ultrasound in Obstetrics and Gynecology. 2021;57(5):726-32. doi: <https://dx.doi.org/10.1002/uog.23590>.

223. Zhang ZY, Wang J, Fan YL, Wang BY, Zhang WT. Effectiveness of neuromuscular electrical stimulation for endometriosis-related pain: A protocol of systematic review and meta-analysis. Medicine (Baltimore). 2020;99(25):e20483. doi: 10.1097/md.0000000000020483. PubMed PMID: 32569169; PubMed Central PMCID: PMCPMC7310956.

224. Mira TAA, Yela DA, Podgaec S, Baracat EC, Benetti-Pinto CL. Hormonal treatment isolated versus hormonal treatment associated with electrotherapy for pelvic pain control in deep endometriosis: Randomized clinical trial. Eur J Obstet Gynecol Reprod Biol. 2020;255:134-41. Epub 20201015. doi: 10.1016/j.ejogrb.2020.10.018. PubMed PMID: 33129015.

225. ChiCtr. A prospective randomized controlled study of postoperative maintenance treatment for patients with endometriosis. <https://trialsearchwhoint/Trial2aspx?TrialID=ChiCTR2000036504>. 2020. PubMed PMID: CN-02185020.

226. Chen W-J, Livneh H, Hsu C-H, Hu Y-T, Lai N-S, Guo H-R, et al. The Relationship of Acupuncture Use to the Endometriosis Risk in Females With Rheumatoid Arthritis: Real-World Evidence From Population-Based Health Claims. Frontiers in Medicine. 2020;7:601606. doi: <https://dx.doi.org/10.3389/fmed.2020.601606>.

227. O'Hara R, Rowe H, Fisher J. Self-management in condition-specific health: A systematic review of the evidence among women diagnosed with endometriosis. BMC Women's Health. 2019;19(1). doi: 10.1186/s12905-019-0774-6.

228. Evans S, Fernandez S, Olive L, Payne LA, Mikocka-Walus A. Psychological and mind-body interventions for endometriosis: A systematic review. J Psychosom Res. 2019;124:109756. Epub 20190627. doi: 10.1016/j.jpsychores.2019.109756. PubMed PMID: 31443810.

229. Dong S, Zhang J, Zhai F, Zhao X, Qin X. Comparative efficacy and safety of traditional Chinese patent medicine for endometriosis: A Bayesian network meta-analysis protocol. MEDICINE. 2019;98(29):e16473. doi: <https://dx.doi.org/10.1097/MD.0000000000016473>.

230. Thabet AAE-M, Alshehri MA. Effect of Pulsed High-Intensity Laser Therapy on Pain, Adhesions, and Quality of Life in Women Having Endometriosis: A Randomized Controlled Trial. PHOTOMEDICINE AND LASER SURGERY. 2018;36(7):363-9. doi: <https://dx.doi.org/10.1089/pho.2017.4419>.

231. Nct. Physiotherapy of the Pelvic Floor in Women With Deep Infiltrating Endometriosis. <https://clinicaltrialsgov/show/NCT03572075>. 2018. PubMed PMID: CN-01660725.

232. Nct. Effect of a Rehabilitation Program to Improve Quality of Life in Women Diagnosed With Endometriosis (Physio-EndEA Study). <https://clinicaltrialsgov/show/NCT03979183>. 2018. PubMed PMID: CN-01933783.

233. Yang Y, Zhao R, Hao Z, Li L, Xu C, Cui Y. Effects of danchi decoction on P450arom, survivin of eutopic endometrium of patients with endometriosis after conservative surgery. African Journal of Traditional, Complementary and Alternative Medicines. 2015;12(4):65-71. doi: 10.4314/ajtcam.v12i4.10. PubMed Central PMCID: PMCBeijing Baoyuan(China).

234. DiVasta AD, Feldman HA, Gallagher JS, Laufer MR, Hornstein MD, Gordon CM. The effect of hormonal add-back therapy in adolescents treated with a gonadotropin releasing hormone (GNRH) agonist for endometriosis: A randomized trial. JOURNAL OF ADOLESCENT HEALTH. 2015;56(2):S24. doi: 10.1016/j.jadohealth.2014.10.049.

235. Bayoglu Tekin Y, Guven S, Kirbas A, Kalkan Y, Tumkaya L, Guvendag Guven ES. Is resveratrol a potential substitute for leuprolide acetate in experimental endometriosis? European Journal of Obstetrics and Gynecology and Reproductive Biology. 2015;184:1-6. doi: 10.1016/j.ejogrb.2014.10.041. PubMed Central PMCID: PMCSigma Aldrich(United States).

236. Vercellini P, Frattaruolo MP, Somigliana E, Jones GL, Consonni D, Alberico D, et al. Surgical versus low-dose progestin treatment for endometriosis-associated severe deep dyspareunia II: Effect on sexual functioning, psychological status and health-related quality of life. HUMAN REPRODUCTION. 2013;28(5):1221-30. doi: 10.1093/humrep/det041. PubMed PMID: WOS:000318108900009.

237. Al-Inany H, Houston B, Farquhar C, Abousetta A. Postoperative application of LNG-IUD for symptomatic endometriosis. HUMAN REPRODUCTION. 2013;28:i223. doi: 10.1093/humrep/det211.

238. Abou-Setta AM, Houston B, Al-Inany HG, Farquhar C. Levonorgestrel-releasing intrauterine device (LNG-IUD) for symptomatic endometriosis following surgery. The Cochrane database of systematic reviews. 2013;(1):CD005072. doi: <https://dx.doi.org/10.1002/14651858.CD005072.pub3>.

239. Vercellini P, Somigliana E, Consonni D, Frattaruolo MP, De Giorgi O, Fedele L. Surgical versus medical treatment for endometriosis-associated severe deep dyspareunia: I. Effect on pain during intercourse and patient satisfaction. HUMAN REPRODUCTION. 2012;27(12):3450-9. doi: 10.1093/humrep/des313. PubMed PMID: WOS:000311644700012.

240. Roshni P, Suneetha Susan Cleave A, Suresh PK. Complementary and alternative medicine (CAM) therapies for management of pain related to endometriosis. International Research Journal of Pharmacy. 2012;3(3):30-4.

241. Api M. Surgery for endometriosis related pain. Journal of Endometriosis. 2012;4(3):133. doi: 10.5301/JE.2012.9727.

242. Wurn BF, Wurn LJ, Patterson K, Richard King C, Scharf ES. Decreasing dyspareunia and dysmenorrhea in women with endometriosis via a manual physical therapy: Results from two independent studies. Journal of Endometriosis. 2011;3(4):188-96. doi: 10.5301/JE.2012.9088.

243. Jin YB, Sun ZL, Jin HF. [Randomized controlled study on ear-electroacupuncture treatment of endometriosis-induced dysmenorrhea in patients]. Zhen Ci Yan Jiu. 2009;34(3):188-92. PubMed PMID: 19761114.

244. Patwardhan S, Nawathe A, Yates D, Harrison GR, Khan KS. Systematic review of the effects of aromatase inhibitors on pain associated with endometriosis. BJOG: An International Journal of Obstetrics and Gynaecology. 2008;115(7):818-22. doi: 10.1111/j.1471-0528.2008.01740.x.

245. Vercellini P, Somigliana E, Cortinovis I, Bracco B, de Braud L, Dridi D, et al. "You can't always get what you want": from doctrine to practicability of study designs for clinical investigation in endometriosis. BMC Womens Health. 2015;15. doi: 10.1186/s12905-015-0248-4. PubMed PMID: WOS:000363297100001.

246. Zhang H, Zhang H, Yang H, Shuid AN, Sandai D, Chen X. Machine learning-based integrated identification of predictive combined diagnostic biomarkers for endometriosis. Frontiers in Genetics. 2023;14. doi: 10.3389/fgene.2023.1290036.

247. Vargas E, García-Moreno E, Aghajanova L, Salumets A, Horcajadas JA, Esteban FJ, et al. The mid-secretory endometrial transcriptomic landscape in endometriosis: a meta-analysis. Hum Reprod Open. 2022;2022(2):hoac016. Epub 20220404. doi: 10.1093/hropen/hoac016. PubMed PMID: 35464885; PubMed Central PMCID: PMCPMC9022214.

248. Powell MJ, Fuller S, Gunderson EP, Benz CC. Reduced cardiovascular risks in women with endometriosis or polycystic ovary syndrome carrying a common functional IGF1R variant. HUMAN REPRODUCTION. 2022;37(5):1083-94. doi: 10.1093/humrep/deac059.

249. Heinze K, Nazeran TM, Lee S, Kramer P, Cairns ES, Chiu DS, et al. Validated biomarker assays confirm that ARID1A loss is confounded with MMR deficiency, CD8+ TIL infiltration, and provides no independent prognostic value in endometriosis-associated ovarian carcinomas. The Journal of pathology. 2022;256(4):388-401. doi: <https://dx.doi.org/10.1002/path.5849>.

250. Bai J, Wang B, Wang T, Ren W. Identification of Functional lncRNAs Associated With Ovarian Endometriosis Based on a ceRNA Network. Front Genet. 2021;12:534054. Epub 20210127. doi: 10.3389/fgene.2021.534054. PubMed PMID: 33584822; PubMed Central PMCID: PMCPMC7873467.

251. Cao XL, Chai J, Yu YY, Tian X, Zhao JY, Yu LY, et al. Association of TNF-α gene T-1031C polymorphism with endometriosis: A meta-analysis. AMERICAN JOURNAL OF REPRODUCTIVE IMMUNOLOGY. 2020;84(6). doi: 10.1111/aji.13305.

252. Proestling K, Birner P, Balendran S, Nirtl N, Marton E, Yerlikaya G, et al. Enhanced expression of the stemness-related factors OCT4, SOX15 and TWIST1 in ectopic endometrium of endometriosis patients. Reproductive biology and endocrinology : RB&E. 2016;14(1):81. doi: <https://dx.doi.org/10.1186/s12958-016-0215-4>.

253. Xin L, Hou Q, Xiong Q, Ding X. Association between matrix metalloproteinase-2 and matrix metalloproteinase-9 polymorphisms and endometriosis: A systematic review and meta-analysis. Biomedical Reports. 2015;3(4):559-65. doi: 10.3892/br.2015.447.

254. Lyu J, Yang H, Lang J, Tan X. Tumor necrosis factor gene polymorphisms and endometriosis in Asians: A systematic review and meta-analysis. CHINESE MEDICAL JOURNAL. 2014;127(9):1761-7. doi: 10.3760/cma.j.issn.0366-6999.20132534.

255. Zhang F, Yang Y, Wang Y. Association between TGF-β1-509C/T polymorphism and endometriosis: A systematic review and meta-analysis. European Journal of Obstetrics and Gynecology and Reproductive Biology. 2012;164(2):121-6. doi: 10.1016/j.ejogrb.2012.05.004.

256. Heilier J-F, Donnez O, Van Kerckhove V, Lison D, Donnez J. Expression of aromatase (P450 aromatase/CYP19) in peritoneal and ovarian endometriotic tissues and deep endometriotic (adenomyotic) nodules of the rectovaginal septum. FERTILITY AND STERILITY. 2006;85(5):1516-8. doi: <https://dx.doi.org/10.1016/j.fertnstert.2005.10.041>.

257. Giese N, Heirs MK. Development of Provisional Acupuncture Guidelines for Pelvic Pain in Endometriosis Using an e-Delphi Consensus Process. Journal of Integrative and Complementary Medicine. 2023;29(3):169-80. doi: 10.1089/jicm.2022.0659.

258. Kitchen H, Seitz C, Trigg A, Aldhouse N, Willgoss T, Schmitz H, et al. Patients' and clinicians' perspectives on item importance, scoring, and clinically meaningful differences for the Endometriosis Symptom Diary (ESD) and Endometriosis Impact Scale (EIS). Health Qual Life Outcomes. 2021;19(1):7. Epub 20210106. doi: 10.1186/s12955-020-01579-7. PubMed PMID: 33407560; PubMed Central PMCID: PMCPMC7789138.

259. Johnson NP, Hummelshoj L, Adamson GD, Keckstein J, Taylor HS, Abrao MS, et al. World Endometriosis Society consensus on the classification of endometriosis. Human reproduction (Oxford, England). 2017;32(2):315-24. doi: <https://dx.doi.org/10.1093/humrep/dew293>.

260. Vanhie A, Meuleman C, Tomassetti C, Timmerman D, D'Hoore A, Wolthuis A, et al. Consensus on Recording Deep Endometriosis Surgery: the CORDES statement. Human reproduction (Oxford, England). 2016;31(6):1219-23. doi: <https://dx.doi.org/10.1093/humrep/dew067>.

261. Bezhenar V. Russian consensus in treatment of endometriosis. Gynecological Surgery. 2014;11(1):97-8. doi: 10.1007/s10397-014-0857-1.

262. Greco CD. Management of adolescent chronic pelvic pain from endometriosis: A pain center perspective. Journal of Pediatric and Adolescent Gynecology. 2003;16(3 SUPPL.):S17-S9. doi: 10.1016/S1083-3188(03)00064-0.

263. Mijatovic V, Vercellini P. Towards comprehensive management of symptomatic endometriosis: beyond the dichotomy of medical versus surgical treatment. HUMAN REPRODUCTION. 2024;39(3):464-77. doi: 10.1093/humrep/dead262.

264. Di Franco S, Alfieri A, Miccio R, Fiore M, Pace MC, Passavanti MB. Palmitoylethanolamide effectiveness and safety for pain relief in animal models: A systematic review. Pain Practice. 2020;20(SUPPL 1):87. doi: 10.1111/papr.12886.

265. Telegdy G, Adamik A, Tanaka M, Schally AV. Effects of the LHRH antagonist Cetrorelix on affective and cognitive functions in rats. Regulatory Peptides. 2010;159(1-3):142-7. doi: 10.1016/j.regpep.2009.08.005. PubMed Central PMCID: PMCAeterna(Germany)

Zentaris(Germany).

266. Telegdy G, Tanaka M, Schally AV. Effects of the LHRH antagonist Cetrorelix on the brain function in mice. NEUROPEPTIDES. 2009;43(3):229-34. doi: 10.1016/j.npep.2009.03.001. PubMed Central PMCID: PMCEterna Zentaris(Germany).

267. Yin B, Jiang H, Liu X, Guo SW. Enriched Environment Decelerates the Development of Endometriosis in Mouse. Reprod Sci. 2020;27(7):1423-35. Epub 20200421. doi: 10.1007/s43032-019-00117-1. PubMed PMID: 32318984.

268. Akbar FS, Farzanegi P, Abbaszadeh H. Effect of a period of swimming exercise and vitamin E intake on catalase and superoxide dismutase activity, and malondialdehyde levels in ovarian tissue of endometriosis model rats. Iranian Journal of Obstetrics, Gynecology and Infertility. 2020;23(9):43-51. doi: 10.22038/IJOGI.2020.17374.

269. Hirsch M, Duffy JMN, Farquhar CM. Re: Hormonal treatment isolated versus hormonal treatment associated with electrotherapy for pelvic pain control in deep endometriosis: Randomized clinical trial. Eur J Obstet Gynecol Reprod Biol. 2021;256:511-2. Epub 20201121. doi: 10.1016/j.ejogrb.2020.11.051. PubMed PMID: 33277060.

270. Hirsch M, Duffy JMN, Farquhar CM. Re: Assessment of levator hiatal area using 3D/4D transperineal ultrasound in women with deep infiltrating endometriosis and superficial dyspareunia treated with pelvic floor muscle physiotherapy: randomized controlled trial. Ultrasound in obstetrics & gynecology : the official journal of the International Society of Ultrasound in Obstetrics and Gynecology. 2021;57(5):849. doi: <https://dx.doi.org/10.1002/uog.23636>.

271. de Mira TAA, Yela DA, Podgaec S, Baracat EC, Benetti-Pinto CL. Reply to Letter to the Editor entitled "Re: Hormonal treatment isolated versus hormonal treatment associated with electrotherapy for pelvic pain control in deep endometriosis: Randomized clinical trial". Eur J Obstet Gynecol Reprod Biol. 2021;258:463-4. Epub 20210116. doi: 10.1016/j.ejogrb.2021.01.022. PubMed PMID: 33485693.

272. Mira TAA, Giraldo PC, Yela DA, Benetti-Pinto CL. Effectiveness of complementary pain treatment for women with deep endometriosis through Transcutaneous Electrical Nerve Stimulation (TENS): randomized controlled trial. European journal of obstetrics, gynecology, and reproductive biology. 2015;194:1-6. doi: <https://dx.doi.org/10.1016/j.ejogrb.2015.07.009>.

273. Mira TAA, Giraldo PC, Yela DA, Benetti-Pinto CL. RETRACTED: Effectiveness of complementary pain treatment for women with deep endometriosis through Transcutaneous Electrical Nerve Stimulation (TENS): randomized controlled trial (Retracted Article). European Journal of Obstetrics & Gynecology and Reproductive Biology. 2015;194:1-6. doi: 10.1016/j.ejogrb.2015.07.009. PubMed PMID: WOS:000365057800001.

274. Nct. The CRESCENDO Program (inCRease Physical Exercise and Sport to Combat ENDOmetriosis). <https://clinicaltrialsgov/show/NCT05831735>. 2023. PubMed PMID: CN-02559712.

275. Barbieri RL. Etiology and epidemiology of endometriosis. Am J Obstet Gynecol. 1990;162(2):565-7. doi: 10.1016/0002-9378(90)90430-f. PubMed PMID: 2309844.

276. Ogawa K, Khan KN, Kuroboshi H, Koshiba A, Shimura K, Tajiri T, et al. Is neonatal uterine bleeding responsible for early-onset endometriosis? Reproductive biology and endocrinology : RB&E. 2023;21(1):56. doi: <https://dx.doi.org/10.1186/s12958-023-01099-1>.

277. Gonçalves AV, Makuch MY, Setubal MS, Barros NF, Bahamondes L. A Qualitative Study on the Practice of Yoga for Women with Pain-Associated Endometriosis. J Altern Complement Med. 2016;22(12):977-82. Epub 20160823. doi: 10.1089/acm.2016.0021. PubMed PMID: 27552065.

278. Adamietz A, Müller A, Boosz A. Diet, physical activity, and complementary medicine: What else is beneficial? A review of current medical trials. Deutsche Zeitschrift fur Akupunktur. 2024;67(2):67-71. doi: 10.1007/s42212-024-00656-y.

279. Tourny C, Zouita A, El Kababi S, Feuillet L, Saeidi A, Laher I, et al. Endometriosis and physical activity: A narrative review. Int J Gynaecol Obstet. 2023;163(3):747-56. Epub 20230622. doi: 10.1002/ijgo.14898. PubMed PMID: 37345574.

280. Falconer L, Hendricks E, Harcourt D. What is the evidence of effectiveness of non-pharmaceutical, non-surgical, biopsychosocial interventions for body image and pain management in individuals with endometriosis? A systematic review. Journal of Endometriosis and Pelvic Pain Disorders. 2022;14(4):206-16. doi: 10.1177/22840265221139909.

281. Hansen S, Sverrisdóttir UÁ, Rudnicki M. Impact of exercise on pain perception in women with endometriosis: A systematic review. ACTA OBSTETRICIA ET GYNECOLOGICA SCANDINAVICA. 2021;100(9):1595-601. doi: 10.1111/aogs.14169.

282. Tennfjord MK, Gabrielsen R, Tellum T. Effect of physical activity and exercise on endometriosis-associated symptoms: a systematic review. BMC Womens Health. 2021;21(1):355. Epub 2021/10/11. doi: 10.1186/s12905-021-01500-4. PubMed PMID: 34627209; PubMed Central PMCID: PMCPMC8502311.

283. Bonocher CM, Montenegro ML, Rosa ESJC, Ferriani RA, Meola J. Endometriosis and physical exercises: a systematic review. Reprod Biol Endocrinol. 2014;12:4. Epub 20140106. doi: 10.1186/1477-7827-12-4. PubMed PMID: 24393293; PubMed Central PMCID: PMCPMC3895811.

284. Cabanas-Barja A, Alonso-Calvete A, Da Cuña-Carrera I. Update on physiotherapy treatment in endometriosis: A systematic review. Clinica e Investigacion en Ginecologia y Obstetricia. 2024;51(1). doi: 10.1016/j.gine.2023.100929.

285. Mira TAA, Buen MM, Borges MG, Yela DA, Benetti-Pinto CL. Systematic review and meta-analysis of complementary treatments for women with symptomatic endometriosis. Int J Gynaecol Obstet. 2018;143(1):2-9. Epub 20180709. doi: 10.1002/ijgo.12576. PubMed PMID: 29944729.

286. Daraï C, Bendifallah S, Foulot H, Ballester M, Chabbert-Buffet N, Daraï E. [Impact of osteopathic manipulative therapy in patient with deep with colorectal endometriosis: A classification based on symptoms and quality of life]. Gynecol Obstet Fertil Senol. 2017;45(9):472-7. Epub 20170830. doi: 10.1016/j.gofs.2017.07.006. PubMed PMID: 28869181.

287. Daraï C, Deboute O, Zacharopoulou C, Laas E, Canlorbe G, Belghiti J, et al. Impact of osteopathic manipulative therapy on quality of life of patients with deep infiltrating endometriosis with colorectal involvement: results of a pilot study. Eur J Obstet Gynecol Reprod Biol. 2015;188:70-3. Epub 20150309. doi: 10.1016/j.ejogrb.2015.03.001. PubMed PMID: 25796057.

288. Del Forno S, Cocchi L, Arena A, Pellizzone V, Lenzi J, Raffone A, et al. Effects of Pelvic Floor Muscle Physiotherapy on Urinary, Bowel, and Sexual Functions in Women with Deep Infiltrating Endometriosis: A Randomized Controlled Trial. Medicina (Kaunas). 2023;60(1). Epub 20231229. doi: 10.3390/medicina60010067. PubMed PMID: 38256327; PubMed Central PMCID: PMCPMC10818504.

289. Merlot B, Elie V, Perigord A, Husson Z, Jubert A, Chanavaz-Lacheray I, et al. Pain Reduction With an Immersive Digital Therapeutic in Women Living With Endometriosis-Related Pelvic Pain: At-Home Self-Administered Randomized Controlled Trial. JOURNAL OF MEDICAL INTERNET RESEARCH. 2023;25. doi: 10.2196/47869. PubMed PMID: WOS:001045687800004.

290. Munoz-Gomez E, Alcaraz-Martinez AM, Molla-Casanova S, Sempere-Rubio N, Aguilar-Rodriguez M, Serra-Ano P, et al. Effectiveness of a Manual Therapy Protocol in Women with Pelvic Pain Due to Endometriosis: A Randomized Clinical Trial. Journal of Clinical Medicine. 2023;12(9). doi: <https://dx.doi.org/10.3390/jcm12093310>.

291. Nieves-Vázquez CI, Detrés-Marquéz AC, Torres-Reverón A, Appleyard CB, Llorens-De Jesús A, Resto IN, et al. Feasibility and acceptability of an adapted environmental enrichment intervention for endometriosis: A pilot study. FRONTIERS IN GLOBAL WOMENS HEALTH. 2023;3. doi: 10.3389/fgwh.2022.1058559. PubMed PMID: WOS:001019289200001.

292. Beaumont T, Phillips K, Hull ML, Green R. Does group physiotherapy improve pain scores and reduce the impact of pelvic pain for women referred with persistent pelvic pain? A clinical trial. Journal of Endometriosis and Pelvic Pain Disorders. 2022;14(4):169-77. doi: 10.1177/22840265221141527.

293. Bennis S, Nikolis L, Adams W, Westbay L, Fitzgerald C. PHYSICAL ACTIVITY IN WOMEN WITH CHRONIC PELVIC PAIN IN A MULTIDISCIPLINARY CLINIC. Female Pelvic Medicine and Reconstructive Surgery. 2022;28(6):S223-S4. doi: 10.1097/SPV.0000000000001202.

294. Escriva-Boulley G, Philip CA, Warembourg S, Lenotre L, Flore P, Faure P, et al. Effects of a physical activity and endometriosis-based education program delivered by videoconference on endometriosis symptoms: the CRESCENDO program (inCRease physical Exercise and Sport to Combat ENDOmetriosis) protocol study. Trials. 2023;24(1):759. Epub 20231127. doi: 10.1186/s13063-023-07792-1. PubMed PMID: 38012776; PubMed Central PMCID: PMCPMC10680283.

295. Mikocka-Walus A, Druitt M, O'Shea M, Skvarc D, Watts JJ, Esterman A, et al. Yoga, cognitive-behavioural therapy versus education to improve quality of life and reduce healthcare costs in people with endometriosis: a randomised controlled trial. BMJ Open. 2021;11(8):e046603. Epub 20210809. doi: 10.1136/bmjopen-2020-046603. PubMed PMID: 34373298; PubMed Central PMCID: PMCPMC8354255.

296. qpm3gt RBR. Physiotherapeutic Exercises in Perineal Pain in Women with Endometriosis. <https://trialsearchwhoint/Trial2aspx?TrialID=RBR-2qpm3gt>. 2023. PubMed PMID: CN-02594499.

297. Salinas-Asensio MDM, Ocón-Hernández O, Mundo-López A, Fernández-Lao C, Peinado FM, Padilla-Vinuesa C, et al. 'Physio-EndEA' Study: A Randomized, Parallel-Group Controlled Trial to Evaluate the Effect of a Supervised and Adapted Therapeutic Exercise Program to Improve Quality of Life in Symptomatic Women Diagnosed with Endometriosis. Int J Environ Res Public Health. 2022;19(3). Epub 20220202. doi: 10.3390/ijerph19031738. PubMed PMID: 35162761; PubMed Central PMCID: PMCPMC8834829.

298. Actrn. Evaluating the influence of Yoga, Cognitive Behaviour Therapy and Standard Care on Quality of Life and Healthcare Costs in Endometriosis. <https://trialsearchwhoint/Trial2aspx?TrialID=ACTRN12620000756921>. 2020. PubMed PMID: CN-02184347.

299. Nct. Effects of Aerobic Exercise and Core Muscle Strengthening on Pain, Menstrual Pattern and QOL in Endometriosis. <https://clinicaltrialsgov/ct2/show/NCT06241040>. 2024. PubMed PMID: CN-02677268.

300. Nct. Effect of Physical Activity and Pain Education on Endometriosis-associated Pain. <https://clinicaltrialsgov/ct2/show/NCT05091268>. 2021. PubMed PMID: CN-02341235.

301. Nct. Effect of Mediterranean Diet and Physical Activity in Patients With Endometriosis. <https://clinicaltrialsgov/show/NCT03994432>. 2019. PubMed PMID: CN-01952735.

302. Ctri. Yoga in the management of pain and related stress on women with Endometriosis. <https://trialsearchwhoint/Trial2aspx?TrialID=CTRI/2017/12/010923>. 2017. PubMed PMID: CN-01897192.

303. Nct. Iyengar Yoga Therapy for Dysmenorrhea and Endometriosis. <https://clinicaltrialsgov/show/NCT03784976>. 2018. PubMed PMID: CN-01701737.

304. Artacho-Cordón F, Salinas-Asensio MDM, Galiano-Castillo N, Ocón-Hernández O, Peinado FM, Mundo-López A, et al. Effect of a Multimodal Supervised Therapeutic Exercise Program on Quality of Life, Pain, and Lumbopelvic Impairments in Women With Endometriosis Unresponsive to Conventional Therapy: a Randomized Controlled Trial. ARCHIVES OF PHYSICAL MEDICINE AND REHABILITATION. 2023;104(11):1785‐95. doi: 10.1016/j.apmr.2023.06.020. PubMed PMID: CN-02585090.

305. Gonçalves AV, Barros NF, Bahamondes L. The Practice of Hatha Yoga for the Treatment of Pain Associated with Endometriosis. Journal of alternative and complementary medicine (New York, NY). 2017;23(1):45‐52. doi: 10.1089/acm.2015.0343. PubMed PMID: CN-01298707.

306. Lutfi M, Dalleck LC, Drummond C, Drummond M, Paparella L, Keith CE, et al. A Single Session of a Digital Health Tool-Delivered Exercise Intervention May Provide Immediate Relief from Pelvic Pain in Women with Endometriosis: A Pilot Randomized Controlled Study. International Journal of Environmental Research and Public Health. 2023;20(3). doi: 10.3390/ijerph20031665.

307. Carpenter SE, Tjaden B, Rock JA, Kimball A. The effect of regular exercise on women receiving danazol for treatment of endometriosis. International journal of gynaecology and obstetrics. 1995;49(3):299‐304. doi: 10.1016/0020-7292(95)02359-k. PubMed PMID: CN-00155419.

308. Bergström I, Freyschuss B, Jacobsson H, Landgren BM. The effect of physical training on bone mineral density in women with endometriosis treated with GnRH analogs: a pilot study. ACTA OBSTETRICIA ET GYNECOLOGICA SCANDINAVICA. 2005;84(4):380‐3. doi: 10.1111/j.0001-6349.2005.00558.x. PubMed PMID: CN-00514975.

309. Zhao L, Wu H, Zhou X, Wang Q, Zhu W, Chen J. Effects of progressive muscular relaxation training on anxiety, depression and quality of life of endometriosis patients under gonadotrophin-releasing hormone agonist therapy. European journal of obstetrics, gynecology, and reproductive biology. 2012;162(2):211‐5. doi: 10.1016/j.ejogrb.2012.02.029. PubMed PMID: CN-00835308.
